# Hardware implementation of memristor-based artificial neural networks

**DOI:** 10.1038/s41467-024-45670-9

**Published:** 2024-03-04

**Authors:** Fernando Aguirre, Abu Sebastian, Manuel Le Gallo, Wenhao Song, Tong Wang, J. Joshua Yang, Wei Lu, Meng-Fan Chang, Daniele Ielmini, Yuchao Yang, Adnan Mehonic, Anthony Kenyon, Marco A. Villena, Juan B. Roldán, Yuting Wu, Hung-Hsi Hsu, Nagarajan Raghavan, Jordi Suñé, Enrique Miranda, Ahmed Eltawil, Gianluca Setti, Kamilya Smagulova, Khaled N. Salama, Olga Krestinskaya, Xiaobing Yan, Kah-Wee Ang, Samarth Jain, Sifan Li, Osamah Alharbi, Sebastian Pazos, Mario Lanza

**Affiliations:** 1 Physical Science and Engineering Division, King Abdullah University of Science and Technology (KAUST), Thuwal, 23955-6900 Saudi Arabia; 2 Departament d’Enginyeria Electrònica, Universitat Autònoma de Barcelona (UAB), 08193 Barcelona, Spain; 3 IBM Research – Zurich, Rüschlikon, Switzerland; 4 Department of Electrical and Computer Engineering, University of Southern California (USC), Los Angeles, CA 90089 USA; 5 Department of Electrical Engineering and Computer Science, University of Michigan, Ann Arbor, MI 48109 USA; 6 Department of Electrical Engineering, National Tsing Hua University, Hsinchu, 30013 Taiwan; 7 Dipartimento di Elettronica, Informazione e Bioingegneria, Politecnico di Milano and IUNET, Piazza L. da Vinci 32, 20133 Milano, Italy; 8 School of Electronic and Computer Engineering, Peking University, Shenzhen, China; 9 Department of Electronic and Electrical Engineering, University College London (UCL), Torrington Place, WC1E 7JE, London, UK; 10 Departamento de Electrónica y Tecnología de Computadores, Facultad de Ciencias, Universidad de Granada, Avenida Fuentenueva s/n, 18071 Granada, Spain; 11 Engineering Product Development (EPD) Pillar, Singapore University of Technology & Design, 8 Somapah Road, 487372 Singapore, Singapore; 12 Computer, Electrical and Mathematical Sciences and Engineering Division, King Abdullah University of Science and Technology (KAUST), Thuwal, 23955-6900 Saudi Arabia; 13 Key Laboratory of Brain-Like Neuromorphic Devices and Systems of Hebei Province, Hebei University, Baoding, 071002 China; 14 Department of Electrical and Computer Engineering, College of Design and Engineering, National University of Singapore (NUS), Singapore, Singapore

**Keywords:** Electrical and electronic engineering, Electronic devices, Electronic devices

## Abstract

Artificial Intelligence (AI) is currently experiencing a bloom driven by deep learning (DL) techniques, which rely on networks of connected simple computing units operating in parallel. The low communication bandwidth between memory and processing units in conventional von Neumann machines does not support the requirements of emerging applications that rely extensively on large sets of data. More recent computing paradigms, such as high parallelization and near-memory computing, help alleviate the data communication bottleneck to some extent, but paradigm- shifting concepts are required. Memristors, a novel beyond-complementary metal-oxide-semiconductor (CMOS) technology, are a promising choice for memory devices due to their unique intrinsic device-level properties, enabling both storing and computing with a small, massively-parallel footprint at low power. Theoretically, this directly translates to a major boost in energy efficiency and computational throughput, but various practical challenges remain. In this work we review the latest efforts for achieving hardware-based memristive artificial neural networks (ANNs), describing with detail the working principia of each block and the different design alternatives with their own advantages and disadvantages, as well as the tools required for accurate estimation of performance metrics. Ultimately, we aim to provide a comprehensive protocol of the materials and methods involved in memristive neural networks to those aiming to start working in this field and the experts looking for a holistic approach.

## Abstract (web summary)

Memristors hold promise for massively-parallel computing at low power. Aguirre et al. provide a comprehensive protocol of the materials and methods for designing memristive artificial neural networks with the detailed working principles of each building block and the tools for performance evaluation.

## Introduction

The development of sophisticated artificial neural networks (ANNs) has become one of the highest priorities of technological companies and governments of wealthy countries, as they can boost the fabrication of artificial intelligence (AI) systems that generate economic and social benefits in multiple fields (e.g., logistics, commerce, health care, national security, etc.)^1^. ANNs are able to compute and store the huge amount of electronic data produced (either by humans or machines), and to execute complex operations with them. Examples of electronic products that contain ANNs with which we interact in our daily lives are those that identify biometric patterns (e.g., face, fingerprint) for access control in smartphones^2^ or online banking apps^3^, and those that identify objects in images from social networks^4^ and security/traffic cameras^5^. Beyond image recognition, other examples are the engines that convert speech to text in computers and smartphones^6^, natural language processing as for example the novel automated chat system chat-GPT^7^, and those that provide accurate recommendations for online shopping based on previous behaviours from ourselves and/or people in our network^8^.

**Fig. 1:**
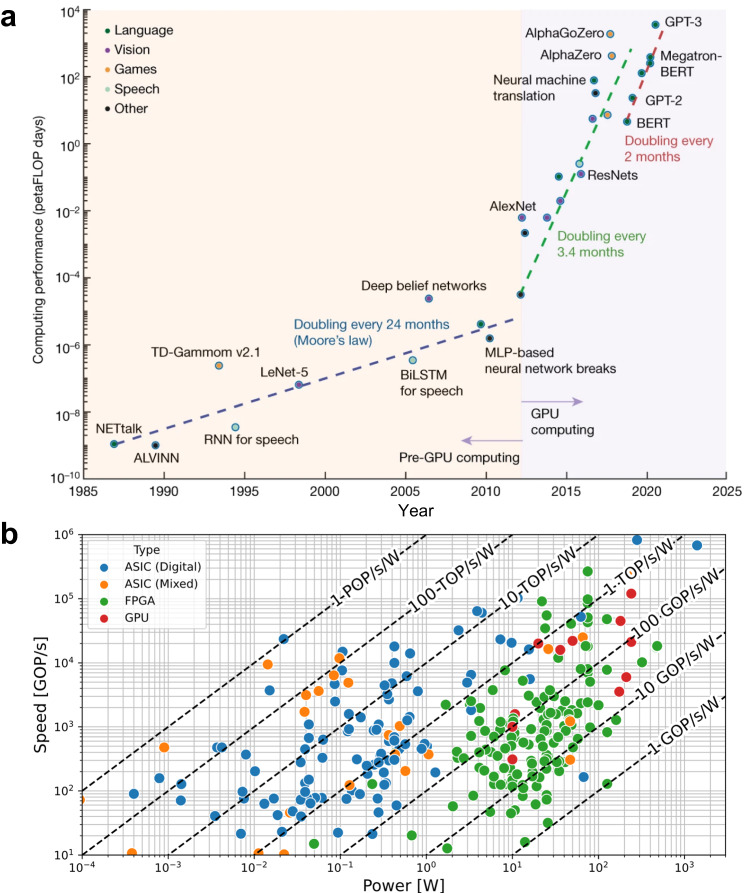
Computing power demand increase and platform transition from Von-Neumann towards highly parallelized architectures. **a** The increase in computing power demands over the past four decades expressed in petaFLOPS per days. Until 2012, computing power demand doubled every 24 months; recently this has shortened to approximately every 2 months. The colour legend indicates different application domains^10^. Mehonic, A., Kenyon, A.J. Brain-inspired computing needs a master plan. Nature 604, 255–260 (2022), reproduced with permission from SNCSC. **b** A comparison of neural network accelerators for FPGA, ASIC, and GPU devices in terms of speed and power consumption. *GOP/s* giga operations per second, *TOP/s* tera operations per second.

ANNs can be understood as the implementation of a sequence of mathematical operations. The structure of ANNs consists of multiple nodes (called neurons) interconnected to each other (by synapses), and the learning is implemented by adjusting the strength (weight) of such connections. Modern ANNs are implemented via software in general-purpose computing systems based on a central processing unit (CPU) and a memory —the so-called Von Neumann architecture^9^. However, in this architecture a large amount of the energy consumption and computing time is related to continuous data exchange between both units, which is not efficient. The computing time can be accelerated by using graphics processing units (GPUs) to implement the ANNs (see Fig. 1a), as these can perform multiple operations in parallel^10–12^. However, this approach consumes even more energy, which requires large computing systems and thereby cannot be integrated in mobile devices. Another option is to use field programable gate arrays (FPGAs), which consume much less energy than GPUs while providing an intermediate computing efficiency between CPUs and GPUs^13–17^. A survey carried out by Guo et al.^18^ on the existing hardware solutions for ANN implementation and their performance is condensed in Fig. 1b.

In the past few years, some companies and universities have presented application specific integrated circuits (ASICs) based on the complementary metal oxide semiconductor (CMOS) technology that are capable to compute and store information in the same unit. This allow such ASICs to perform multiple operations in parallel very fast, making them capable of mimicking, directly in the hardware, the behaviour of the neurons and synapses in the ANN. A comprehensive list of these ASICs comprising those such as the Google TPU^19^, Amazon inferentia^20^, Tesla NPU^21^, etc., are summarized in ref. ^22^. Such integrated circuits can be grouped in two categories. On one hand, dataflow processors are custom-designed processors for neural network inference and training. Since neural network training and inference computations can be entirely deterministically laid out, they are amenable to dataflow processing in which computations, memory accesses, and inter-ALU communications actions are explicitly/statically programmed or placed-and-routed onto the computational hardware. On the other hand, processor in memory (PIM) accelerators integrate processing elements with memory technology. Among such PIM accelerators are those based on an analogue computing technology that augments flash memory circuits with in-place analogue multiply-add capabilities. Please refer to the references for the Mythic^23^ and Gyrfalcon^24^ accelerators for more details on this innovative technology.

Previously mentioned ANNs and those reported in detail in the survey presented in ref. ^22^ belongs to the subgroup of so-called deep neural networks (DNNs). In a DNN the information is represented with values that are continuous in time and can achieve high data recognition accuracy by using at least two layers of nonlinear neurons interconnected by adjustable synaptic weights^25^. Conversely, there is an alternative information codification which gave birth to another type of ANNs, the Spiking Neural Networks (SNN). In SNNs the information is coded with time-dependent spikes, which remarkably reduces the power consumption compared to DNNs^26^. Moreover, the functioning of SNNs is more similar to the actual functioning of biological neural networks, and it can help to understand complex mammal’s neural systems. Intel probably has the most extensive research program for evaluating the commercial viability of SNN accelerators with their Loihi technology^27,28^, and Intel Neuromorphic Development Community^29^. Among the applications that have been explored with Loihi are target classification in synthetic aperture radar and optical imagery^30^, automotive scene analysis^31^, and spectrogram encoder^27^. Further, one company, Innatera, has announced a commercial SNN processor^32^. Also, the platforms developed by IBM (TrueNorth^33^), and Tsingshua^34^ are well known examples of the research effort of both the industry and the academia in this field.

However, fully-CMOS implementations of ANNs require tens of devices to simulate each synapse, which threatens energy and area efficiency, and thereby renders large-scale systems impractical. As a result, the performance of CMOS-based ANNs is still very far from that of biological neural networks. To emulate the complexity and ultra-low power consumption of biological neural networks, hardware platforms for ANNs must achieve an ultra-high integration density (>1 Terabyte per cm^2^) and low energy consumption (<10 fJ per operation)^35^.

Recent studies have proposed that the use of memristive devices to emulate the synapses may accelerate ANN computational tasks while reducing the overall power consumption and footprint^36–42^. Memristive devices are materials systems whose electrical resistance can be adjusted to two or more stable (i.e., non-volatile) states by applying electrical stresses^43^. Memristive devices that exhibit two non-volatile states are already being commercialized as standalone memory^44,45^, although their global market is still small (~621 million USD by 2020, i.e., ~0.5% of the 127-billion-worth standalone memory market^46^). However, memristive devices can also exhibit three disruptive attributes particularly suitable for the hardware implementation of ANNs: i) the possibility to program multiple non-volatile states (up to ~100^47,48^, and even ~1000^49^), ii) a low-energy consumption for switching (~10fJ per state transition with zero-static consumption when idle^50^), and iii) a scalable structure appropriate for matrix integration (often referred to as crossbar^51^) and even 3D stacking^52^. Moreover, the switching time can be as short as 85 ps^42^.

So far, several groups and companies have claimed the realization of hybrid CMOS/memristor implementations of ANNs^53–61^, —from now on, memristive ANNs— with performance that is superior to that of fully-CMOS counterparts. However, most of those studies in fact only measured the figures-of-merit of one/few devices and simulated the accuracy of an ANN via software^62–67^ in such type of studies the connection between the memristors fabricated and the ANN is relatively weak. Few studies went beyond that and built/characterized crossbar arrays of memristive devices^48,68–70^, but that are still very far from real full-hardware implementations of all the mathematical operations required by the ANN. The most advanced studies in this field have reported fully integrated memristor-based compute-in-memory systems^48,53–55,58,59,61,71–73^, but a systematic description of essential details on the device structure or circuit architecture are generally lacking in these reports.

In this article we provide a comprehensive step-by-step description of the hardware implementation of memristive ANNs for image classification —the most studied application often used to benchmark performance, describing all the necessary building blocks and the information processing flow. For clarity, we consider relatively simple networks, being the multilayer perceptron the most complex case. We take into account the challenges arising at both the device and circuit levels and discuss a SPICE-based approach for their study in the design stage, as well as the required circuital topologies for the fabrication of a memristive ANN.

## Structure of memristor-based ANNs

**Fig. 2:**
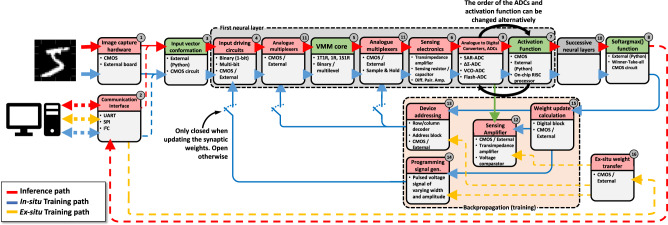
Generalized block diagram indicating the required circuital blocks to implement a memristive ANN for pattern classification. Green blocks (3, 5, 7 and 8) indicate the required mathematical operations (such as the VMM or activation functions). Red blocks (1, 2, 4, 6, 9, 11, 12, 13, 14, 15, 16) identify the required circuits for signal adaptation and/or conversion. The data path followed during the inference (or forward pass) is indicated by the red arrows/lines. The data path followed for in-situ training is indicated by the blue arrows/lines. The data path followed under ex-situ training is shown by the yellow arrows/lines. For each box, the upper (colored) part indicates the name of the function to realize by the circuital block, and the bottom part indicates the type of hardware required. The box titled successive neural layers would encompass multiple sub-blocks with a structure similar to the group titled First neural layer. 1S1R stands for 1Selector 1 Resistor while 1R stands for 1 Resistor. UART, SPI and I^2^C are well known communication standards. RISC stands for Reduced Instruction Set Computer.

Figure 2 shows a flowgraph depicting the generalized structure of an ANN; it has multiple inputs (for single channel images like indexed color, grayscale and bitmap images, there are as many inputs as pixels the image to classify has) and several outputs (as many as types/classes of images the ANN will recognize). As it can be seen, the ANN consists of multiple mathematical operations (green boxes), such as vector matrix multiplication (VMM), activation function, and softargmax function. Among all the critical operations in the ANN, the VMM is the most complex and demanding, and it is carried out multiple times both during the training process and inference. Hence, the development of new hardware for ANN implementation is strongly oriented to realize VMM operations in a more efficient way. Interestingly, the VMM operation —often understood as multiply and accumulate (MAC) routine—can be implemented using a crossbar array of memory elements. Those memory devices could be either charge-based memories as well as resistance-based memories^25,74^.

**Fig. 3:**
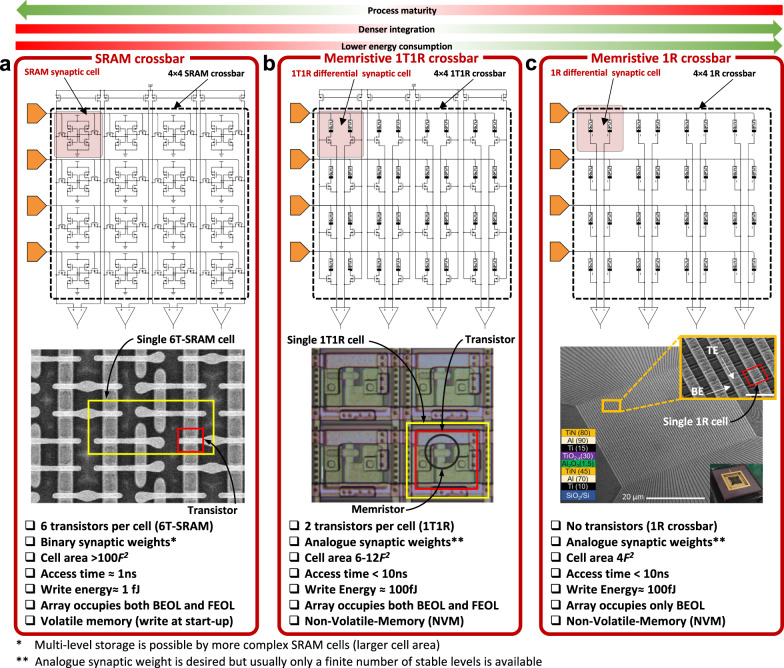
Non-Von Neumann vector-matrix-multiplication (VMM) cores reported in the literature. **a** Full-CMOS SRAM (Static Random Access Memory) crossbar array, **b** Hybrid memristor/CMOS 1T1R crossbar array and **c** Full-memristive passive crossbar array. All cases assume a crossbar array integration structure which performs the Multiply-and-Accumulate (MAC) by exploiting the Kirchhoff’s law of currents. The use of memristors allows a smaller footprint per synapse as a lower number of smaller devices is employed. Passive crossbar arrays of memristors allow the highest possible integration density, yet they are still an immature technology with plenty of room for optimization. **a**^290^ Yamaoka, M. Low-Power SRAM. In: Kawahara, T., Mizuno, H. (eds) Green Computing with Emerging Memory. Springer, New York, NY (2013), reproduced with permission from SNCSC. **b** is adapted with permission under CC BY 4.0 license from ref. ^54^. **c** is adapted with permission under CC BY 4.0 license from ref. ^93^. *F* is the feature size of the litography and the energy estimation is on the cell-level. FEOL and BEOL stands for Front End Of Line and Back End Of Line, respectively.

Before explaining memristive hardware for ANN, in this paragraph we describe the state of the art of CMOS hardware for ANNs, to provide the author with a comprehensive picture of the different technologies available for hardware based ANNs. Among charge-based memories, SRAM cells (a bi-stable transistor structure typically made of two CMOS inverters connected back-to-back which retains a charge concentration, see Fig. 3a for an example of the structure of a crossbar array of 6T SRAM) have been widely used for VMM^75–77^. If the elements of the input vector and the weight matrix are limited to signed binary values, the multiply operation is simplified to a combination of XNOR and ADD functions carried out directly through SRAM cells. An example of this is the work by Khwa et al., which reports a compute in memory system based on a crossbar array of 6T SRAM memory cells as binary synaptic connections that uses binary inputs/outputs^78^. The proposed circuit comprises 4 kb synapses fabricated in a 65 nm CMOS process and reported an energy efficiency of 55.8 TOPS per W. In cases where x is non-binary, one approach is to employ capacitors in addition to the SRAM cells^76,77,79^, involving a three-step process. However, a major drawback of SRAM memories is their volatile nature. Due to the low field-effect transistor barrier height (0.5 eV), the charge constantly needs to be replenished from an external source and hence SRAM always needs to be connected to a power supply. An alternative memory element for VMM operation is the flash memory cell^80,81^, in which the charge storage node is coupled to the gate of a FET with charge stored either on a conductive electrode surrounded by insulators (floating gate) or in discrete traps within a defective insulator layer (charge trapping layer). Unlike in SRAM, the barrier height of the storage node is sufficiently high for long-term data retention. Also, flash-based VMM operates in a slightly different manner than SRAM-based VMM. In Flash-based VMM, each memory element contribute a different amount to the current in each column of the crossbar depending on the voltage applied to the input or crossbar row and matrix element are stored as charge on the floating gate^81^ (i.e., multiplication) and all the currents in a column are instantaneously summed (i.e., accumulation) by Kirchhoff’s currents law. Because the devices can be accessed in parallel along a BL, NOR Flash has generally been preferred over NAND Flash for in-memory computing. This is the case of the work by Fick et al from the company Mythic^23^, which relies on a 1024×1024 NOR Flash array to develop an analogue matrix processor for human pose detection in real time video processing. However, there is recent work describing the use of 3D NAND, consisting of vertically stacked layers of serially connected FLASH devices, whereby each layer of the array encodes a unique matrix^82^. This approach could help to overcome the scalability issue of NOR Flash, which is difficult to scale beyond the 28 nm technology node. The proposed 3D-aCortex accelerator^83^ is a fully CMOS implementation that relies on a commercial 3D-NAND flash crossbar array as synaptic element. Partial outputs from multiple crossbars are temporally aggregated and digitized using digital counters, shared by all the crossbars along a row of the grid, avoiding the communication overhead of performing these reductions across multiple levels of hierarchy. The entire 3D array shares a global memory and a column of peripheral circuits, increasing its storage efficiency. This is however still theoretical and is yet to be fabricated. Nonetheless, the write operation on flash memories requires high voltages (typically >10 V) and entails significant latency (>10 µs) due to the need to overcome the storage node barriers. These problems can be potentially solved using resistance-based memories, or memristors as memory element at the intersections of the crossbar, as they can realize the multiplication operation by Ohm’s Law (*I=V·G*, where *I* is current, *V* is the input voltage and *G* is the conductance of each memristor), while reducing the energy consumption and area footprint as well as providing CMOS compatible operation voltages. The structure of memristive crossbar arrays for VMM is depicted in Fig. 3b, c: a common integration option is to place a CMOS transistor in series with the memristor to control the current through it (Fig. 3b) in a so called 1 transistor 1 resistor (1T1R) structure, while the highest integration density would be achieved by a crossbar comprising no transistors, i.e., considering cells usually referred to as 1 resistor/memristor (1R or 1M) structures or passive crossbar (Fig. 3c). When using crossbar arrays of memristors to perform VMM operations, additional circuitry might be needed at the input and output to sense and/or convert electrical signals (see red boxes in Fig. 2). Examples of such circuits are digital-to-analogue (DAC), analogue-to-digital (ADC) converters and transimpedance amplifiers (TIA). Note that other studies employed implementations slightly different from this scheme, i.e., combining or avoiding certain blocks to save area and/or reduce power consumption (see Table 1).

**Table 1:** List of reported prototypes in the literature and the detail of how was implemented each block (Software/Hardware Off-chip/Hardware On-chip, etc)

Work(s)	Device	NN Type/ Dataset	Crossbar size	CMOS Node	ADC	Cell Structure	Input circuit (DAC)	Sensing Electronics	Activation function	Row/Col. Selectors	Softmax Activation Func.	Inference/training	Weight Prog. circuitry
^57^	Au/Pd/WO_X_/Au	SLP, Sparse coding, MLP/Greek letters	54 × 108	180 nm	On-chip (13-bit)	1R	On-chip (6-bit)	Charge integration	On-chip digital (Sigmoid)	On-chip	Off-chip (Software)	Inference & training	On-chip
^55^	TiN/TaOx/HfOx /TiN	CNN/MNIST	128 × 16	130 nm	Off-chip (8-bit)	1T1R	On-chip (1-bit)	Charge integration	Off-chip (software: ReLU and max. Pooling)	On-chip	Off-chip (Software)	Inference & training	Off-chip
^102^	Pt/Ta/Ta2O5/Pt/Ti	MLP/MNIST	128 × 64	2 µm	N/A	1T1R	N/A	N/A	Off-chip hardware: ReLU)	Off-chip	Off-chip (Software)	Learning & training	Off-chip
^61^	No data (propietary dev.)	BNN, MNIST, CIFAR-10	128 × 64	90 nm	On-chip (3-bit)	1T1R	Not implemented	On-chip (VSA)	On-chip (Binary)	On-chip	Off-chip (software)*	Inference only	Off-chip
^113^	Ta/TaOx/Pt	CNN/MNIST	64 × 64	180 nm	On-chip	1T1R	On-chip	On-chip (TIA)	Off-chip (software)*	On-chip	Off-chip (software)*	Inference only	Off-chip
^114,115^	TaOx	CNN/MNIST	64 × 64	180 nm	On-chip (10-bit)	1T1R	On-chip	On-chip (TIA)	Off-chip (software)*	On-chip	Off-chip (software)*	Inference only	Off-chip
^219^,	W/TiN/TiON	BNN/MNIST	100 × 100	65 nm	On-chip (3-bit)	1T1R	N/A	On-chip (CSA)	Off-chip (FPGA: max. Pooling)	On-chip	Off-chip (FPGA)	Inference only	Off-chip
^116^	Pt/SiOxAg/Pt/Ti, Ta/Pd/HfO2/Pt/Ti	CNN/ ‘U’, ‘M, ‘A’, ‘S’	8 × 8	No data	Off-chip	1T1R	On-chip	Off-chip (TIA)	On-chip (ReLU), Off-chip (software: max. Pooling)	Off-chip	Off-chip (MCU)	Inference & training	Off-chip
^272^	TiN/HfO2/Ti/TiN	BNN/MNIST, CIFAR-10	1 Kb	130 nm	On-chip	2T2R	Not implemented	On-chip (PCSA)	On-chip (Binary)	On-chip	On-chip (Binary)	Inference only	Off-chip
^99^	W/Ta_2_O_5_/TaO_x_/W	MLP/MNIST	2 Mb	180 nm	On-chip (1-bit)	1T1R	On-chip (1-bit)	On-chip	No data	On-chip	No data	Inference only	Off-chip
^100^	AlCu/TiN/Ti/HfO_2_/TiN	MLP/	32 × 32	150 nm	On-chip (1 or 3-bit)	1T1R	On-chip (1-bit)	On-chip	Off-chip (software)*	On-chip	Off-chip (software)*	Inference only	On-chip (SRAM)
^122^	PCM (no more data)	MLP/MNIST	512 × 1024	180 nm	No data	3T1C+ 2PCM	No data	Off-chip (software)	Off-chip (Software: ReLU)	Off-chip	Off-chip (Software)	Inference only	Off-chip
^71,73^	PCM (no more data)	MLP/MNIST, ResNET-9/CIFAR-10	256 × 256	14 nm	On-chip	4T4R	On-chip (8-bit)	On-chip (CCO-based)	On-chip (ReLU)	On-chip	Off-chip (Software)	Inference only	On-chip
^72^,	PCM (no more data)	MLP/MNIST	512 × 512	14 nm	Off-chip	4T4R	On-chip (8-bit)	On-chip	Off-chip (Sigmoid)	On-chip	Off-chip (FPGA)	Inference only	On-chip
^273^	No data	CNN/ CIFAR-10	256 × 512	55 nm	On-chip	1T1R	No data	On-chip	Off-chip (FPGA)	On-chip	Off-chip (FPGA)	Inference only	Off-chip
^274^	TiN/HfO2/Ti/TiN	CNN/MNIST	18 kB	130 nm	Off-chip*	1T1R	Off-chip*	Off-chip*	Off-chip (FPGA)	Off-chip*	Off-chip (FPGA)	Inference only	On-chip
^123^	TiN/HfO2/Ti/TiN	BNN/MNIST	1 Kb	130 nm	N/A	2T2R	N/A	On-chip	Off-chip (software)*	On-chip	Off-chip (software)*	Inference only	Off-chip
^275^	-/HfO_2_/TaO_X_/-	MLP/MNIST	158.8 Kb	130 nm	On-chip (8-bit)	2T2R	On-chip (8-bit)	Charge integration	Off-chip	On-chip	Off-chip	Inference only	Off-chip (FPGA)
^60^	TiN/HfO_2_/TaO_X_/TiN	CNN/MNIST, CIFAR-10	256×256	130 nm	On-chip (8-bit)	1T1R	On-chip	Charge integration	On-chip (analog: ReLU), Off-chip (FPGA: max. Pooling)	On-chip	Off-chip (FPGA)	Off-chip (Software)	On-chip

*Assumed as no information is provided.

In the following subsections we describe in detail all the circuital blocks required for a truly full-hardware implementation of a memristive ANN. To provide both a clear global picture and detailed explanations, the titles of the sub-sections correspond to the names of the blocks in Fig. 2.

### Image capture hardware (block 1) and input vector conformation (block 3)

An image (or pattern) is a collection of pixels with different colours arranged in a matrix form (referred as *p×p* in this article). In this work, we will consider grayscale images, in which the colour of those pixels can be codified by one single value. However, in coloured images, each pixel is represented by 3 (in RGB encoding) or 4 (in CMYK encoding) values, this arranged in a tensor fashion, i.e., *p×p×3* or *p×p×4*. Both the training and testing of an ANN for image classification are conducted by presenting large datasets of images to its inputs. In a real ANN each image could come directly from an embedded camera (block 1), or it could be provided as a file by the user (block 2). Depending on the format of the image (e.g., black/white, 8-bit *.bmp, 24-bit *.bmp, *.jpg, *.png, among many others) the range of possible colours (encoded as numerical values) for each pixel will be different. Each of the above mentioned approaches to feed images to the neural network implies different hardware overhead. For the case of on-the-fly image classification, a CMOS imager is necessary to capture the input images^82,83^. For instance, ref. ^84^ uses a 480×330 pixel image sensor, with each pixel consisting of a photo diode and four transistors that generates an analogue signal whose amplitude is proportional to the light intensity. Then a 5×6 pixel binary image is generated by mapping 96×55 neighbourhood pixels into one pixel in the binary image. A similar approach is considered in ref. ^85^ where a 640×480 pixels image is captured by an image sensor and then resized to a 16×16 image. The resizing procedure and the need of such a procedure will be covered later in this Sub-section. Both cases consider an FPGA in order interface the image acquisition system (i.e. CMOS image sensor and the resizing algorithm) with the memristor crossbar and its peripheral circuitry. On the other hand, some studies exclusively focused on the memristor crossbar use an on-chip communication interface to acquire the image from a computer (e.g. ref. ^54^ uses a serial communication port) already shaped in the required input format.

**Fig. 4:**
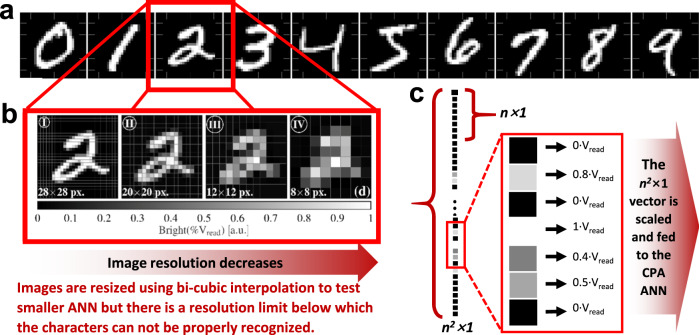
Example of a widely popular image database used for ANNs training and test, and how they are feed to the network. **a** Samples of the MNIST dataset of handwritten numeric digits considered in this article. In all cases images are represented in 28 × 28 px. Pixel brightness (or intensity) is codified in 256 levels ranging from 0 (fully OFF, black) to 1 (fully ON, white). **b** Readability loss as the resolution decreases from 28 × 28 pixels (case I) to 8 × 8 (case IV). **c** Schematic representation of the unrolling of the image pixels. Note that each of the *n* image columns of pixels are vertically concatenated to reach a n^2^ × 1 column vector. It is then scaled by *V*_READ_ to produce a vector of analogue voltages that is fed to the ANN.

Regarding the input images, there are multiple datasets of images online available for ANN training and testing. Some of the most commonly used ones are: 1) MNIST (Modified National Institute of Standards and Technology), which is basically a dataset containing 70,000 greyscale images showing handwritten numbers from 0 to 9 (i.e., around 7,000 for each number); 60,000 of them used for training and 10,000 for testing^86^; 2) CIFAR (Canadian Institute for Advanced Research), which contains 60, 000 color images divided into 10 classes for CIFAR-10 and 100 classes for CIFAR-100^87^; 3) ImageNet, one of the largest image datasets, which consists of over 1.2 million labelled from 1000 classes for the ImageNet competition^88^. MNIST is a good starting point, since this simple dataset can be classified with even small neural networks. For benchmarking a device or a chip, it is essential to evaluate the accuracy of standard deep neural network models like VGG^89^ and ResNet^90^ on CIFAR and ImageNet dataset by utilizing architecture-level simulation and realistic hardware statistics^91,92^. For clarity, here we illustrate with MNIST dataset. The number of types/classes of images (referred to as *m* in this article) in the MNIST dataset is 10. The images are compressed in a *.idx3-ubyte file that can be opened with MATLAB; each of them comes in grayscale and with a resolution of 28×28 pixels. In Python, the MNIST images can be found embedded in a library named Keras. The training images are used to let the ANN understand the characteristic features of each pattern (i.e., the numbers), and the testing images are presented to the ANN (after training) to be classified. A few examples of these images can be seen in Fig. 4a, where the X and Y axis stand for the pixel index. Pixel’s brightness is codified in 256 grey levels between 0 (fully OFF, black) and 255 (fully ON, white). In the MNIST dataset, each of the 60,000 *p×p* training images is represented as a *p*^2^*×*1 column vector, and all these vectors are horizontally concatenated to render a *p*^2^*×*60,000 matrix. Similarly, the test dataset consists of a *p*^2^*×*10,000 matrix. In both cases, each of the *p*^2^ pixels must be fed to the crossbar array for further processing.

As previously mentioned, the simplest ANN architectures (multi-layer perceptrons) should have as many inputs as pixels there are in the images to be classified. In software based ANNs, this is not a challenge. However, the available inputs in hardware ANNs are limited by the maximal size of the memristor crossbar. In the literature, such a challenge has been tackled considering different approaches: For instance, given the MNIST dataset in which images have a resolution of 28 × 28 pixels one option is to implement the synaptic layer using multiple crossbars to fit the 784 inputs (e.g., 13 64 × 64 or 4 256 × 256 crossbars would be needed^93^). However, for research efforts focused on the device level, this is usually out of reach as requires a non-straightforward CMOS – memristor integration. Another option is to consider more complex neural networks, such as the convolutional neural networks (CNN)^55^. LeNet-5 (a kind of CNN) first layer is 25 × 6, which can be implemented with a 64×64 crossbar. In fact, image classification tasks in modern deep learning usually rely on a convolutional layer. As for the previous case, this is not easy to implement for research projects centred on the device level as it also requires complex hybrid CMOS – memristor integration. Nonetheless, in some cases, the first convolutional layers are implemented on software and off-chip to reduce the image dimensionality and then the resulting feature vector is feed to the memristive part of the ANN. Note that in this case, device non-idealities are not equally represented through-out the network, and their influence is only assessed for the fully-connected part^55^. Finally, other option is to rescale each of the images of the original MNIST dataset (in this work, represented by block 3). For example, if our crossbar has 64 inputs, then the image would have to be rescaled from 28 × 28 to 8 × 8 (i.e., 64 pixels); the size of the rescaled image will be referred as *n×n*. The rescaling can be easily done via software, using for example MATLAB and its Deep Learning Toolbox as language/platform to carry out this type of computational operations, or Python altogether with the TensorFlow, Keras or Pytorch libraries. However, and as shown in Fig. 4b, the aggressively rescaled images becomes barely readable and therefore the entire dataset is changed and so it is the benchmark, i.e. inference results obtained for the 8×8 MNIST rescaled images should only be compared with 8×8 MNIST results and not with the original MNIST benchmark results. This is similar to using a custom-made dataset. With this in mind, and provided the frequent use of this methodology in the literature, we will consider its usage yet stressing the aforementioned considerations, and we encourage authors not to rescale the image dataset if aiming to compare their results against the original datasets.

As an example, Supplementary Algorithm 1 shows the MATLAB code used for image dataset rescaling from 28 × 28 to 8 × 8 pixels. Before downscaling the images, each of them needs to be reshaped from a *p*^2^*×1* column vector to a *p×p* matrix, using the MATLAB function reshape(). Then, the image is resized to the desired *n×n* size in pixels by the MATLAB function imresize()^94^. This function receives as argument the desired down-sampling method, which in this example was selected to be the bi-cubic interpolation (as in other articles in the field of memristive ANNs^54^). The results of the rescaling for a single image are shown in Fig. 4b. Note that using this method, values outside the [0, 1] range are expected. Thereby, the downscaled image is processed and any output value exceeding such range is truncated to 0 or 1. The re-scaled images are then reshaped back to the *n*^2^×1 column vector representation format and stored in a new matrix. Now this image can be used as input in the crossbar array of memristors.

### Input driving circuits (Block 4)

**Fig. 5:**
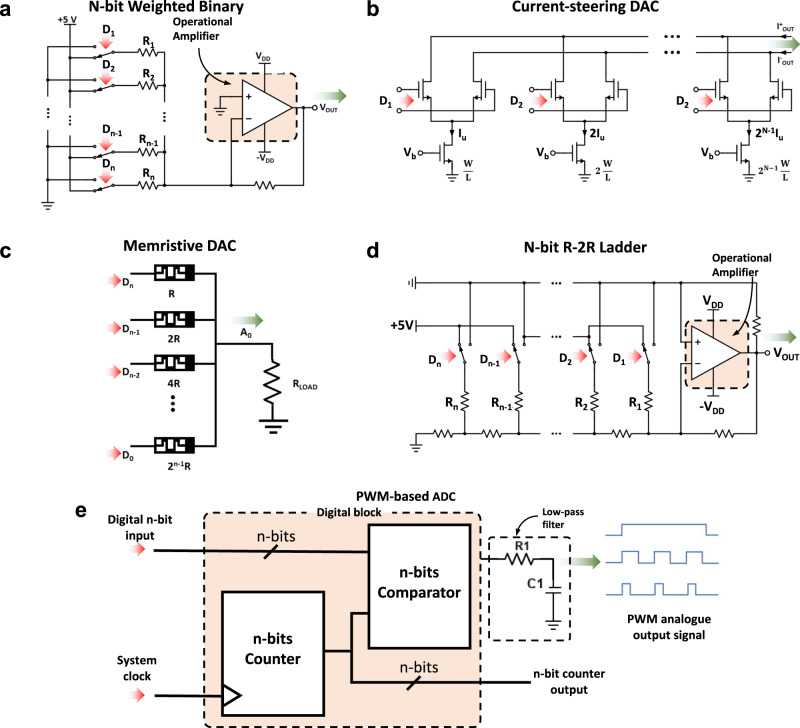
Schematic diagrams of DAC circuits conventionally used in the literature to bias the rows of the memristive crossbar. **a** N-bit weighted Binary, **b** Current-steering DAC, **c** Memristive-DAC **d** N-bit R-2R DAC and **e** Pulse Width Modulation (PWM)-based DAC.

The colour of each pixel in the image (represented as *n*^2^ *× 1* column) is codified as a voltage that is applied to a row in the crossbar (i.e., word-line), as depicted in Fig. 4c, resulting in a vector **V** of analogue voltages *V*_i_. If the image is black-white (i.e., 2 possible values), the values of the voltage *V*_i_ of each pixel will be 0 and *V*_READ_ (*V*_READ_ being a reference voltage defined by the application); however, the colour of each pixel can also range within a greyscale, which leads to a range of analogue voltages. For instance, the colour of each pixel in the 8-bits *p×p* images of the MNIST dataset (and hence, the colour of each pixel in the resized *n×n* image to be input to the crossbar) varies within a greyscale of 2^8^ = 256 possible values (codified in binary representation from 00000000 to 11111111), meaning that the voltages to be applied to each input of the crossbar may take values such as 0V, *V*_READ_/256, 2·*V*_READ_/256, etcetera until *V*_READ_. Hence, an 8-bit digital-to-analogue converter (DACs) is necessary for each input to convert the 8-bits-code into a single voltage. When the ANN is employed to recognize other types of images codified with a different format (e.g., 24-bit), DACs of different resolution are needed. The format in which the images are presented depends on the ultimate application of the network, i.e., ANNs for plate number identification may work well with black/white (i.e., 1-bit) images, and ANNs for object identification may need to consider 24 bits (16.7 million) colours. Examples of DACs often employed in memristive ANNs are displayed in Fig. 5: N-bit weighted Binary (Fig. 5a), Current-steering DAC Fig. 5b, Memristive-DAC (Fig. 5c), N-bit R-2R DAC (Fig. 5d) and Pulse Width Modulation (PWM)-based DAC (Fig. 5e).

Deciding the resolution of the DACs at the input of each row of the crossbar is a critical factor affecting power consumption, area, and output impedance of the ANN —lowering impedance is important to realize large crossbars. Conventional high-resolution DACs with a low output impedance comprise a DAC core with an operational amplifier (in a buffer configuration) as output stage in order to lower the output resistance. As such, the power dissipation of the DAC can be divided into the switching/leakage power of the digital DAC core and the static/dynamic power of the operational amplifier. On one hand, the power dissipation of the digital DAC core can be estimated as *P*_D_ *= f*_D_*C*_D_*V*^2^*+P*_leakage_, where *f*_D_ is the output frequency*, C*_D_ is the parasitic capacitance*, V* is the supply voltage, and *P*_leakage_ is the leakage power that depends on the technology node, and for a 65 nm technology with a 1 V power supply is of several pico-Watts in an inverter. On the other hand, the power dissipation of the analogue block can be estimated by assuming a class-AB follower stage, with an efficiency of 50%. In this scenario the static power of this block equals its dynamic power and the addition of them can be computed as *P*_A_*=nV/R*^2^, where *n* is the number of memristors to drive and *R* is their minimum resistance. Below frequencies of roughly 100 MHz, *P*_A_ is dominant, whereas above this threshold, the dissipated power during the switching makes *P*_D_ bigger than *P*_A_.

Regarding the silicon area required for the DACs, this is mainly defined by the DAC resolution, which in turn is limited by device noise element matching. For DAC relying in resistors, the major noise source is from the CMOS operational amplifier in the output stage^95^, and it can be minimized using larger transistors (both in width and length) for the differential input pair. Similarly, to maximize the matching between the reference resistors, wider devices are encouraged, ultimately contributing to the increase in the silicon area required per DAC.

To minimize silicon area and power consumption, the lower the DAC resolution the better. As a result, apart from amplitude-based encoding for crossbar inputs, time-encoding schemes are also considered^96^. For instance, in pulse-width modulation (PWM) schemes, inputs are codified in different pulse widths (0 s, *T*_READ_/256 s, 2·*T*_READ_/256 s, etc. until *T*_READ_). This allows overcoming device non-linearity but suffers from low throughput^57^. Alternatively, in the so-called bit-serial encoding^97^ approaches, high-resolution crossbar inputs are presented as a stream of voltage pulses with constant amplitude and width^48,56^. For example, to represent 16-bit crossbar inputs, *m*-bit voltage signals are streamed to the crossbar row over 16/*m* time cycles^98^. After VMM calculation, the partial products (the outputs of each time step) are accumulated together to form the final output value. Also, many papers^55,60,99,100^, have explored the case of ANNs with binarized inputs, as they employ the simplest DACs (1-bit). In the case of the 1-bit input stream, DACs can also be replaced by inverters followed by an output amplifier to allow the inverter to drive all the devices connected to it^98^. In addition, the computation with time-encoded inputs is less affected by the noise variations, which mostly affect the amplitude of the input signals rather than the pulse width. However, the disadvantage of time-encoding schemes is the reduction of computation speed and hardware overhead required for partial sums computation^96^.

An alternative to keep a high throughput and still employ a low-resolution DAC is using approximate computing^101^. When using low-resolution DACs (1-, 2- or 3-bit) there is a higher chance of multiple inputs requiring the same driving voltage, which allows sharing DACs among several lines, and thereby saving both power and area. However, one has to keep in mind that the output resistance of the DAC limits the number wordlines that can be biased. Also, this approach requires the use of analogue multiplexers (block 11) in between the input driving circuits and the memristor crossbar which leads to additional control circuit overhead. The problem of using low-resolution DACs at the input of the crossbar is a loss in the accuracy of the VMM operation. Hence, there is an inherent trade-off between all these variables. The accuracy loss can also be reduced by exploiting software-based training techniques for quantized neural networks.

### VMM core (Block 5)

**Fig. 6:**
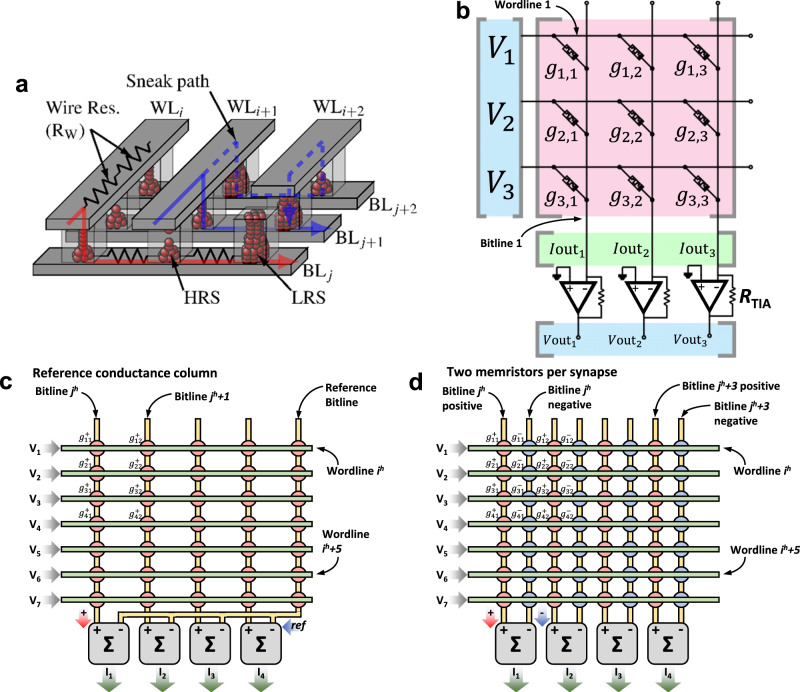
Memristor crossbar structure and electrical connection diagram for signed weights representation. **a** Sketch of the crossbar array structure. Red and blue arrows exemplify the electron flow through the memristors connecting the top (Word lines -WL-) and bottom lines (Bit lines -BL-). Different memristor resistance states are schematically represented (High Resistance State -HRS- to Low Resistance State -LRS-). The dashed blue line depicts the so-called sneak path problem. The parasitic wire resistance is indicated for WL_i_ and BL_i_. Reproduced with permission under CCBY 4.0 license from ref. ^253^. **b** Equivalent circuit representation of the CPA sketched in **a**, showing the input voltages, output currents and TIA blocks that translates the output CPA current to a vector of analogue voltages. In this case the circuit was simplified by ignoring the line resistances. Finally, two different realizations of the memristive-based ANN synaptic layer are shown in **c** – unbalanced – and **d** – balanced –.

The voltages generated by each DAC (which represent the colour of each pixel of the rescaled *n*^2^ *×* 1 image) are applied at the inputs (rows) of the *n*^2^ *× m* crossbar array of memristors. The conductance of each memristor within the crossbar describes the synaptic connection between each input neuron (*i*th) and each output neuron (*j*th). This scheme is used in various papers^54,102^. However, some others consider also a bias term added to the weighted sum fed to the neuron^57^. This can be done digitally and off-chip, or in the analogue domain. If done analogue, an additionally row in the crossbar is needed, thereby requiring a crossbar of (*n*^2^*+*1*) × m*. This operation produces a row vector of size 1 *×* m (see Eq. 1). In a conventional Von Neumann computing system, VMM is performed by doing each sub-operation (multiplications and sums) sequentially, which is time consuming; moreover the calculation time increases quadratically with the dimensionality of the input arrays^103^, or in the case of using the so-called Big-O notation, the VMM algorithm has a time complexity of ~*O(n*^2^*)*. Memristor crossbars (such as the one shown in Fig. 6a) allow performing VMM much more easily and faster because all the sub-operations are carried out in parallel. In the crossbar, the brightness (colour) of each pixel in each image is codified in terms of analogue voltages and applied to the input rows (also called wordlines and connected to the memristor’s top electrodes), while the output columns (also called bitlines and connected to the memristor’s bottom electrodes) are grounded through a transimpedance amplifier (see Fig. 6b for an idealized representation). Then, the VMM is performed in an analogue fashion, as the current flowing through each memristor will be given by the voltage applied to the line and the conductance of each memristor (*I*_ij_* = g*_ij_*·V*_i_). Note that in a pair {i,j} i stands for the crossbar row, and j for the crossbar column. Then, the currents flowing through the memristors connected to a given bitline are summed and sensed to form the output vector. Let us consider the following notation to better explain this idea:

1 $$\left[\begin{array}{cccc}{V}_{1} & {V}_{2} & \cdots & {V}_{{{{{{{\rm{n}}}}}}}^{2}}\end{array}\right]\times 	\left[\begin{array}{cccc}{g}_{1,1} & {g}_{1,2} & \cdots & {g}_{1,{{{{{\rm{m}}}}}}}\\ {g}_{2,1} & {g}_{2,2} & \cdots & {g}_{2,{{{{{\rm{m}}}}}}}\\ \vdots & \vdots & \ddots & \vdots \\ {g}_{{{{{{{\rm{n}}}}}}}^{2},1} & {g}_{{{{{{{\rm{n}}}}}}}^{2},2} & \cdots & {g}_{{{{{{{\rm{n}}}}}}}^{2},{{{{{\rm{m}}}}}}}\end{array}\right] \\ 	=\left[\begin{array}{cccc}\mathop{\sum }\limits_{i=1}^{{n}^{2}}{V}_{{{{{{\rm{i}}}}}}}{{\cdot }}{g}_{{{{{{\rm{i}}}}}},1} & \mathop{\sum }\limits_{i=1}^{{n}^{2}}{V}_{{{{{{\rm{i}}}}}}}{{\cdot }}{g}_{{{{{{\rm{i}}}}}},2} & \cdots & \mathop{\sum }\limits_{i=1}^{{n}^{2}}{V}_{{{{{{\rm{i}}}}}}}{{\cdot }}{g}_{{{{{{\rm{i}}}}}},{{{{{\rm{m}}}}}}}\end{array}\right]$$

For the classification of the MNIST images with a *n×n* pixel resolution with an ANN, multiple VMM operations are required, in which the matrix of conductances *g*_ij_ in Eq. 1 is defined based on the matrix ${{{{{{\bf{W}}}}}}}_{{{{{{\bf{M}}}}}}}$ of synaptic weights, which has a size of *n*^2^ × 10, and all the numbers that form it are real numbers (${{{{{{\bf{W}}}}}}}_{{{{{{\bf{M}}}}}}}\in {{\mathbb{R}}}^{{n}^{2}\times 10}$) with both positive and negative values being possible —the way in which ${{{{{{\bf{W}}}}}}}_{{{{{{\bf{M}}}}}}}$ is calculated is described in detail in section ANN training and synaptic weight update (Blocks 2, 11-15): Learning algorithm. As the negative values cannot be represented directly with memristors, some strategies have been adopted. Reference ^104^ added an extra column in the crossbar (named reference column, see blue arrow in Fig. 6c) with all its memristors set to 0.5·*G*_LRS_, so totalling *n*^2^ × (*m* + 1) memristors in the crossbar. Then, the total current at the {j} output of the crossbar is obtained by subtracting the current generated by the reference column {*ref*} to the current generated from a {j} column (see Fig. 6c). This concept is mathematically represented in Eq. 2.

2 $$	\left[\mathop{\sum }\limits_{i=1}^{{n}^{2}}{V}_{{{{{{\rm{i}}}}}}}{{\cdot }}{g}_{{{{{{\rm{i}}}}}},1} \mathop{\sum }\limits_{i=1}^{{n}^{2}}{V}_{{{{{{\rm{i}}}}}}}{{\cdot }}{g}_{{{{{{\rm{i}}}}}},2} \cdots \mathop{\sum }\limits_{i=1}^{{n}^{2}}{V}_{{{{{{\rm{i}}}}}}}{{\cdot }}{g}_{{{{{{\rm{i}}}}}},{{{{{\rm{m}}}}}}}\right]\to \\ 	 \left[\mathop{\sum }\limits_{i=1}^{{n}^{2}}{V}_{i}{{\cdot }}\left({{g}^{{\prime} }}_{{{{{{\rm{i}}}}}},1}-{g}_{{{{{{\rm{ref}}}}}}}\right) \mathop{\sum }\limits_{i=1}^{{n}^{2}}{V}_{i}{{\cdot }}\left({{g}^{{\prime} }}_{{{{{{\rm{i}}}}}},2}-{g}_{{{{{{\rm{ref}}}}}}}\right) \cdots \mathop{\sum }\limits_{i=1}^{{n}^{2}}{V}_{i}{{\cdot }}\left({{g}^{{\prime} }}_{{{{{{\rm{i}}}}}},{{{{{\rm{m}}}}}}}-{g}_{{{{{{\rm{ref}}}}}}}\right)\right]$$

where *g*_ref_ stands for the 0.5·*G*_LRS_ conductances of the reference column and ${{g}^{{\prime} }}_{{{{{{\rm{i}}}}}},{{{{{\rm{j}}}}}}}$ is calculated in such a way that devices with a conductance above 0.5·*G*_LRS_ produce positive synaptic weights, and those with a conductance below 0.5·*G*_LRS_ produce negative synaptic weights^104^. This strategy has two disadvantages: on one hand, one can only employ half of the states exhibited by the memristor for the positive weights and the other half for the negative weights, thus reducing the range between the maximum and minimum weight. On the other hand, routing the reference column to the rest of the crossbar columns to make the corresponding subtraction operation, is not trivial. Another strategy is to use two memristors per synaptic weight, resulting in two crossbars of *n*^2^ *×* 10 (20*n*^2^ synapses)^105,106^. Within this approach, Eq. 2 could be re-written as

3 $$	\left[\mathop{\sum }\limits_{i=1}^{{n}^{2}}{V}_{{{{{{\rm{i}}}}}}}{{\cdot }}{g}_{{{{{{\rm{i}}}}}},1} \mathop{\sum }\limits_{i=1}^{{n}^{2}}{V}_{{{{{{\rm{i}}}}}}}{{\cdot }}{g}_{{{{{{\rm{i}}}}}},2} \cdots \mathop{\sum }\limits_{i=1}^{{n}^{2}}{V}_{{{{{{\rm{i}}}}}}}{{\cdot }}{g}_{{{{{{\rm{i}}}}}},{{{{{\rm{m}}}}}}}\right]\\ 	 \to \left[\mathop{\sum }\limits_{i=1}^{{n}^{2}}{V}_{{{{{{\rm{i}}}}}}}{{\cdot }}\left({{g}^{+}}_{{{{{{\rm{i}}}}}},1}-{{g}^{-}}_{{{{{{\rm{i}}}}}},1}\right) \mathop{\sum }\limits_{i=1}^{{n}^{2}}{V}_{{{{{{\rm{i}}}}}}}{{\cdot }}\left({{g}^{+}}_{{{{{{\rm{i}}}}}},2}-{{g}^{-}}_{{{{{{\rm{i}}}}}},2}\right) \cdots \mathop{\sum }\limits_{i=1}^{{n}^{2}}{V}_{i}{{\cdot }}\left({{g}^{+}}_{{{{{{\rm{i}}}}}},{{{{{\rm{m}}}}}}}-{{g}^{-}}_{{{{{{\rm{i}}}}}},{{{{{\rm{m}}}}}}}\right)\right]$$

Where the positive and negative conductances are codified by a pair of two adjacent memristors (${{g}^{+}}_{{{{{{\rm{i}}}}}},{{{{{\rm{j}}}}}}}$ and ${{g}^{-}}_{{{{{{\rm{i}}}}}},{{{{{\rm{j}}}}}}}$), each of them set to a positive value of conductance. This representation method, shown in Fig. 6d, has been chosen in this study because it doubles the range of conductance levels of the crossbar, making it less susceptible to noise and variability^104^.

To calculate the required conductance value for each of the memristors in the pair, we begin by splitting **W**_**M**_ into two matrices ${{{{{{\bf{W}}}}}}}_{{{{{{\bf{M}}}}}}}^{{{{{{\boldsymbol{+}}}}}}}$ and ${{{{{{\bf{W}}}}}}}_{{{{{{\bf{M}}}}}}}^{{{{{{\boldsymbol{-}}}}}}}$ as:

4 $$\begin{array}{c}{w}_{{M}_{{{{{{\rm{i}}}}}},{{{{{\rm{j}}}}}}}}^{+}\left\{\begin{array}{cc}{w}_{{M}_{i,j}},& {w}_{{M}_{{{{{{\rm{i}}}}}},{{{{{\rm{j}}}}}}}} > \,0\\ 0,& {w}_{{M}_{{{{{{\rm{i}}}}}},{{{{{\rm{j}}}}}}}}\le 0\end{array}\right.\\ {w}_{{M}_{{{{{{\rm{i}}}}}},{{{{{\rm{j}}}}}}}}^{-}\left\{\begin{array}{cc}0,& {w}_{{M}_{{{{{{\rm{i}}}}}},{{{{{\rm{j}}}}}}}}\ge 0\\ -{w}_{{M}_{i,j}},& {w}_{{M}_{{{{{{\rm{i}}}}}},{{{{{\rm{j}}}}}}}} < \,0\end{array}\right.\end{array}$$

each of them containing only positive weights, so that ${{{{{{\bf{W}}}}}}}_{{{{{{\bf{M}}}}}}}{{{{{\boldsymbol{=}}}}}}{{{{{{\bf{W}}}}}}}_{{{{{{\bf{M}}}}}}}^{{{{{{\boldsymbol{+}}}}}}}{{{{{\boldsymbol{-}}}}}}{{{{{{\bf{W}}}}}}}_{{{{{{\bf{M}}}}}}}^{{{{{{\boldsymbol{-}}}}}}}$. The matrix in the left side (${{{{{{\bf{W}}}}}}}_{{{{{{\bf{M}}}}}}}$, containing both positive and negative values) can be represented as a difference between the two matrices in the right side (${{{{{{\bf{W}}}}}}}_{{{{{{\bf{M}}}}}}}^{{{{{{\boldsymbol{+}}}}}}}$ and ${{{{{{\bf{W}}}}}}}_{{{{{{\bf{M}}}}}}}^{{{{{{\boldsymbol{-}}}}}}}$, both containing only positive numbers). Thereby, by applying Eq. 4, we obtain ${{{{{{\bf{W}}}}}}}_{{{{{{\bf{M}}}}}}}^{{{{{{\boldsymbol{+}}}}}}}$ by replacing all the negative elements from ${{{{{{\bf{W}}}}}}}_{{{{{{\bf{M}}}}}}}$ by 0, while ${{{{{{\bf{W}}}}}}}_{{{{{{\bf{M}}}}}}}^{{{{{{\boldsymbol{-}}}}}}}$ was obtained by first multiplying matrix ${{{{{{\bf{W}}}}}}}_{{{{{{\bf{M}}}}}}}$ by -1 and then replacing al the negative values by 0.

In the next step, the conductance matrices ${{{{{{\bf{G}}}}}}}_{{{{{{\bf{M}}}}}}}^{{{{{{\boldsymbol{+}}}}}}}$ and ${{{{{{\bf{G}}}}}}}_{{{{{{\bf{M}}}}}}}^{{{{{{\boldsymbol{-}}}}}}}$ (Equation 5) to be mapped into the crossbars are calculated by employing a linear transformation,^107,108^:

5 $$\begin{array}{c}{{{{{{\boldsymbol{G}}}}}}}_{{{{{{\boldsymbol{M}}}}}}}^{{{{{{\boldsymbol{+}}}}}}}={a\cdot }{{{{{{\bf{W}}}}}}}_{{{{{{\bf{M}}}}}}}^{{{{{{\boldsymbol{+}}}}}}}+b=\frac{{G}_{\max }-{G}_{\min }}{\max \left\{{{{{{{\bf{W}}}}}}}_{{{{{{\bf{M}}}}}}}\right\}-\min \left\{{{{{{{\bf{W}}}}}}}_{{{{{{\bf{M}}}}}}}\right\}}{{{{{{\bf{W}}}}}}}_{{{{{{\bf{M}}}}}}}^{{{{{{\boldsymbol{+}}}}}}}+\left[{G}_{\max }-\frac{\left({G}_{\max }-{G}_{\min }\right)\max \left\{{{{{{{\bf{W}}}}}}}_{{{{{{\bf{M}}}}}}}\right\}}{\max \left\{{{{{{{\bf{W}}}}}}}_{{{{{{\bf{M}}}}}}}\right\}-\min \left\{{{{{{{\bf{W}}}}}}}_{{{{{{\bf{M}}}}}}}\right\}}\right]\\ {{{{{{\boldsymbol{G}}}}}}}_{{{{{{\boldsymbol{M}}}}}}}^{{{{{{\boldsymbol{-}}}}}}}={a\cdot }{{{{{{\bf{W}}}}}}}_{{{{{{\bf{M}}}}}}}^{{{{{{\boldsymbol{-}}}}}}}+b=\frac{{G}_{\max }-{G}_{\min }}{\max \left\{{{{{{{\bf{W}}}}}}}_{{{{{{\bf{M}}}}}}}\right\}-\min \left\{{{{{{{\bf{W}}}}}}}_{{{{{{\bf{M}}}}}}}\right\}}{{{{{{\bf{W}}}}}}}_{{{{{{\bf{M}}}}}}}^{{{{{{\boldsymbol{-}}}}}}}+\left[{G}_{\max }-\frac{\left({G}_{\max }-{G}_{\min }\right)\max \left\{{{{{{{\bf{W}}}}}}}_{{{{{{\bf{M}}}}}}}\right\}}{\max \left\{{{{{{{\bf{W}}}}}}}_{{{{{{\bf{M}}}}}}}\right\}-\min \left\{{{{{{{\bf{W}}}}}}}_{{{{{{\bf{M}}}}}}}\right\}}\right]\end{array}$$

here ${G}_{\min }$ and ${G}_{\max }$ are the minimal and maximal conductance values of the memristors in the crossbar, and $\max \left\{\right.{{{{{\bf{W}}}}}}_{{{{{{\bf{M}}}}}}}$} and $\min \left\{\right.{{{{{\bf{W}}}}}}_{{{{{{\bf{M}}}}}}}$} are the maximum and minimum values in **W**_**M**_. At this point, it is critical to note that this mapping strategy presents the synaptic weights from **W**_**M**_ to a continuum of conductance values in the range $\left[{G}_{\min },{G}_{\max }\right]$. However, it has been widely reported^109–111^, that the more states one memristor has, the more difficult to identify them, due to the inherent variability. Moreover, depending on the material and fabrication methods, some memristor devices can have only a limited number of stable conductance states. To deal with these non idealities, advanced mapping techniques have been proposed in the literature and they are summarized in Supplementary Note 1 and Supplementary Note 2, the latter focused on mitigating the heat-induced drift of synaptic weights. Thereby, when considering a device with a number *x* of states, each position of the resulting conductance matrices should have only *x* possible values. In order to exploit the entire dynamic range of the memristors (which would make easier to identify each conductance value), we consider ${G}_{\max }={G}_{{{{{{\rm{LRS}}}}}}}$ and ${G}_{\min }={G}_{{{{{{\rm{HRS}}}}}}}$, being *G*_LRS_ and *G*_HRS_ the conductance of the most and least conductive states (respectively). In this way, the synaptic weights in the ${{{{{{\bf{W}}}}}}}_{{{{{{\bf{M}}}}}}}^{{{{{{\boldsymbol{+}}}}}}}$ and ${{{{{{\bf{W}}}}}}}_{{{{{{\bf{M}}}}}}}^{{{{{{\boldsymbol{-}}}}}}}$ matrices are converted to conductance values within the range $\left[{G}_{{{{{{\rm{HRS}}}}}}},{G}_{{{{{{\rm{LRS}}}}}}}\right].$The following example illustrates the procedure to convert the **W**_**M**_ matrix returned by the MATLAB training phase (i.e., a matrix of real values in the range [−5, 5]) into two crossbar arrays of memristors (considering that each memristor can have 6 linearly distributed resistive states at *G*_HRS_, 0.2·*G*_LRS_, 0.4·*G*_LRS_, 0.6·*G*_LRS_, 0.8·*G*_LRS_ and *G*_LRS_):

First, the ex-situ training produces a matrix of *n*^*2*^*×m* synaptic weights:

6 $${{{{{{\bf{W}}}}}}}_{{{{{{\bf{M}}}}}}}=\left[\begin{array}{ccccc}1.1 & 4.7 & -3.9 & \ldots & 4.9\\ 1.8 & -3 & -1.2 & \ldots & 0.2\\ 4.6 & -4.9 & 0.3 & \ldots & 1.3\\ \vdots & \vdots & \vdots & \ddots & \vdots \\ -0.9 & 2.7 & -2.2 & \ldots & -4.8\end{array}\right]$$

Second, the synaptic weights are represented as the difference between two matrices:

7 $${{{{{{\bf{W}}}}}}}_{{{{{{\bf{M}}}}}}}^{{{{{{\boldsymbol{+}}}}}}}{{{{{\boldsymbol{-}}}}}}{{{{{{\bf{W}}}}}}}_{{{{{{\bf{M}}}}}}}^{{{{{{\boldsymbol{-}}}}}}}=\left[\begin{array}{ccccc}1.1 & 4.7 & 0 & \ldots & 4.9\\ 1.8 & 0 & 0 & \ldots & 0.2\\ 4.6 & 0 & 0.3 & \ldots & 1.3\\ \vdots & \vdots & \vdots & \ddots & \vdots \\ 0 & 2.7 & 0 & \ldots & 0\end{array}\right]-\left[\begin{array}{ccccc}0 & 0 & 3.9 & \ldots & 0\\ 0 & 3 & 1.2 & \ldots & 0\\ 0 & 4.9 & 0 & \ldots & 0\\ \vdots & \vdots & \vdots & \ddots & \vdots \\ 0.9 & 0 & 2.2 & \ldots & 4.8\end{array}\right]$$

Third, the weights are rounded to the closest state among the *x* available states:

8 $${{{{{{{\bf{W}}}}}}}_{{{{{{\bf{M}}}}}}}^{{{{{{\boldsymbol{+}}}}}}}}_{{{{{{\bf{q}}}}}}}{{{{{\boldsymbol{-}}}}}}{{{{{{{\bf{W}}}}}}}_{{{{{{\bf{M}}}}}}}^{{{{{{\boldsymbol{-}}}}}}}}_{{{{{{\bf{q}}}}}}}=\left[\begin{array}{ccccc}1 & 5 & 0 & \ldots & 5\\ 2 & 0 & 0 & \ldots & 0\\ 5 & 0 & 0 & \ldots & 1\\ \vdots & \vdots & \vdots & \ddots & \vdots \\ 0 & 3 & 0 & \ldots & 0\end{array}\right]-\left[\begin{array}{ccccc}0 & 0 & 4 & \ldots & 0\\ 0 & 3 & 1 & \ldots & 0\\ 0 & 5 & 0 & \ldots & 0\\ \vdots & \vdots & \vdots & \ddots & \vdots \\ 1 & 0 & 2 & \ldots & 5\end{array}\right]$$

Finally, the quantized weights are mapped to a conductance value:

9 $${{{{{{\bf{G}}}}}}}_{{{{{{\bf{M}}}}}}}^{{{{{{\boldsymbol{+}}}}}}}{{{{{\boldsymbol{-}}}}}}{{{{{{\bf{G}}}}}}}_{{{{{{\bf{M}}}}}}}^{{{{{{\boldsymbol{-}}}}}}}=\left[\begin{array}{ccccc}\frac{{G}_{{{{{{\rm{LRS}}}}}}}}{5} & {G}_{{{{{{\rm{LRS}}}}}}} & {G}_{{{{{{\rm{HRS}}}}}}} & \ldots & {G}_{{{{{{\rm{LRS}}}}}}}\\ \frac{2{G}_{{{{{{\rm{LRS}}}}}}}}{5} & {G}_{{{{{{\rm{HRS}}}}}}} & {G}_{{{{{{\rm{HRS}}}}}}} & \ldots & {G}_{{{{{{\rm{HRS}}}}}}}\\ {G}_{{{{{{\rm{LRS}}}}}}} & {G}_{{{{{{\rm{HRS}}}}}}} & {G}_{{{{{{\rm{HRS}}}}}}} & \ldots & \frac{{G}_{{{{{{\rm{LRS}}}}}}}}{5}\\ \vdots & \vdots & \vdots & \ddots & \vdots \\ {G}_{{{{{{\rm{HRS}}}}}}} & \frac{3{G}_{{{{{{\rm{LRS}}}}}}}}{5} & {G}_{{{{{{\rm{HRS}}}}}}} & \ldots & {G}_{{{{{{\rm{HRS}}}}}}}\end{array}\right]-\left[\begin{array}{ccccc}{G}_{{{{{{\rm{HRS}}}}}}} & {G}_{{{{{{\rm{LRS}}}}}}} & \frac{4{G}_{{{{{{\rm{LRS}}}}}}}}{5} & \ldots & {G}_{{{{{{\rm{LRS}}}}}}}\\ {G}_{{{{{{\rm{HRS}}}}}}} & \frac{3{G}_{{{{{{\rm{LRS}}}}}}}}{5} & \frac{{G}_{{{{{{\rm{LRS}}}}}}}}{5} & \ldots & {G}_{{{{{{\rm{HRS}}}}}}}\\ {G}_{{{{{{\rm{HRS}}}}}}} & {G}_{{{{{{\rm{LRS}}}}}}} & {G}_{{{{{{\rm{HRS}}}}}}} & \ldots & {G}_{{{{{{\rm{HRS}}}}}}}\\ \vdots & \vdots & \vdots & \ddots & \vdots \\ {G}_{{{{{{\rm{LRS}}}}}}} & {G}_{{{{{{\rm{HRS}}}}}}} & \frac{2{G}_{{{{{{\rm{LRS}}}}}}}}{5} & \ldots & {G}_{{{{{{\rm{LRS}}}}}}}\end{array}\right]$$

The output value caused by a negative synaptic weight is achieved by subtracting the current flowing through the memristors connected to bitline *i* in ${{{{{{\bf{G}}}}}}}_{{{{{{\bf{M}}}}}}}^{{{{{{\boldsymbol{-}}}}}}}$ matrix from that in the corresponding bitline *i* in ${{{{{{\bf{G}}}}}}}_{{{{{{\bf{M}}}}}}}^{{{{{{\boldsymbol{+}}}}}}}$ matrix.

### Sensing electronics (Block 6)

**Fig. 7:**
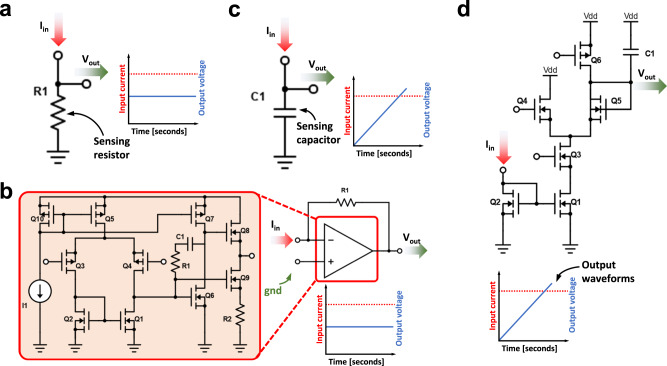
Circuit schematics for the sensing electronics placed in at the output of every column of the memristive crossbar. In all cases, the goal is to translate a current signal into a voltage signal. **a** The sensing resistor is the simplest case, as it translates current into voltage directly by the Ohm’s law. **b** The use of a TIA allows to connect the crossbar columns to 0 volts and operate with lower output currents. As well as in the resistor-based approach, the current voltage conversion is linear when operating the TIA within its linear range and the output voltage signal is immediately available as soon as the output of the TIA settles. **c** For currents below the nano-ampere regime, charge integration is the most suitable option for current-voltage conversion. This can be achieved by using a capacitor. As such, the measurement is not instantaneous as a constant, controllable integration time is required before the measurement. **d** To minimize the area requirements of the integration capacitor, the use of a current divider allows to further reduce the current and, with it, the size of the required capacitor. The tradeoff in this case is with precision (mainly due to transistor mismatch) and output voltage dynamic range.

Once the input voltages are applied to the inputs (rows) of the crossbar, currents at the outputs (columns) are almost instantaneously generated, which need to be sensed. There are three widely used sensing modes for the output voltages^112^. The simplest approach is the use of a sensing resistor (Fig. 7a). However, grounding the bitlines through a resistor might alter the potential applied to the bitline, which will no longer be 0 volts, adding variability and thus altering the read over the sensing resistor^100,112^. To sense low currents without this problem, one option is to use trans-impedance amplifiers (TIA, see Fig. 7b). In this case, the crossbar bitlines are grounded through a TIA implemented with an operational amplifier or an operational transconductance amplifier which ensures the bitline potential to remain at 0 V. Although very popular^102,113–116^, this approach might be limited for the case of the smallest technology nodes implementations as the gain and bandwidth of the amplifiers are limited by the intrinsic transistor gain^95,117^. An alternative is to replace the TIA block by a charge-based accumulation circuit. This strategy was used to cope with pulse width modulation encoding that excludes the utilization of one TIA. Note that the same approach could be used along with other encoding techniques such as digitization of inputs and pulse amplitude modulation. In its most basic implementation, it is very similar to the use of a sensing resistor but replacing the resistor by a capacitor (see Fig. 7c). The capacitor then develops a voltage which is proportional to the integrated current flowing through it. As such, this method adds the time-dimension to the process of sensing the outputs: the current must be integrated over a constant and well-defined period of time to generate an output voltage. Note that in many cases, to reduce the current to be integrated (and thus the size of the integration capacitors), current divider circuits^57^ or differential pair integrators^118^ are considered (see Fig. 7d).

**Fig. 8:**
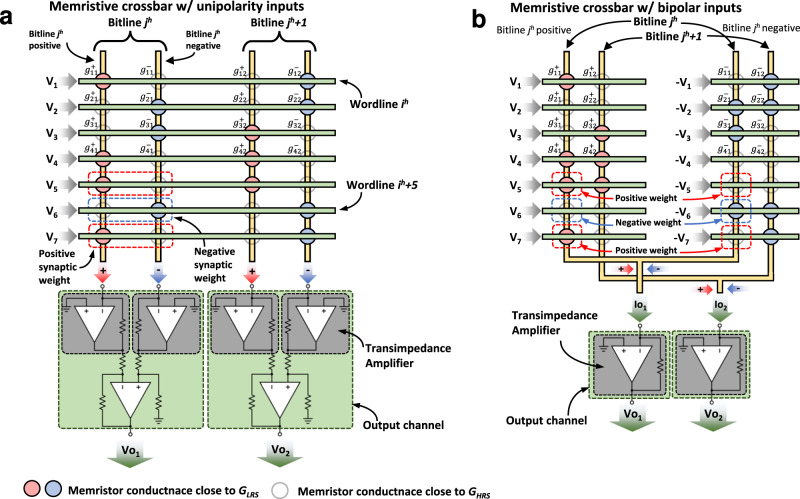
Equivalent electrical circuit of the topology used to implement the mathematical difference between two electrical signals. **a** Assuming that voltage inputs are unipolar (that is, only negative or positive), it is required to first transduce the current signals into voltage and then add an operational amplifier in a subtractor configuration. **b** If bipolar signals can be applied in the inputs, by biasing the negative synaptic weights with a voltage or opposite polarity, summing the resulting currents in a common node (Kirchhoff’s Law for Current) already solves the subtraction operation, and only one transimpedance amplifier is required per column.

Finally, note that the design choice of the sensing circuit will depend on the input signals to the memristor crossbar, as shown in Fig. 8. Assuming that the input signals of both positive and negative cells are of the same polarity, an independent sensing/transducing circuit is required for both the positive and negative bitline. Then a subtractor circuit (implemented for instance with an operational amplifier, as shown in Fig. 8a) generates an output voltage proportional to the current difference. On the contrary, when it is possible to apply input signals of different polarity to the ${{{{{{\bf{G}}}}}}}_{{{{{{\bf{M}}}}}}}^{{{{{{\boldsymbol{-}}}}}}}$ and ${{{{{{\bf{G}}}}}}}_{{{{{{\bf{M}}}}}}}^{{{{{{\boldsymbol{+}}}}}}}$ matrix, the sensing electronics can be simplified, as by connecting the *i* bitlines from the ${{{{{{\bf{G}}}}}}}_{{{{{{\bf{M}}}}}}}^{{{{{{\boldsymbol{-}}}}}}}$ and ${{{{{{\bf{G}}}}}}}_{{{{{{\bf{M}}}}}}}^{{{{{{\boldsymbol{+}}}}}}}$ directly performs the substraction in terms of currents, and thereby only one sensing amplifier is needed (as shown by the single transimpedance amplifier in Fig. 8b).

### Activation function (Block 7)

Ideally, the output current of each bitline (column) pair in a crossbar-based implementation of a VMM is a linear-weighted sum of all the wordlines (rows) connected to such column. Since a combination of linear functions results in a new linear function, complex nonlinear relationships could not be replicated by an ANN regardless of the number of the linear neural layers considered. This problem can be overcome by introducing a non-linearity transformation on the weighted sum output by each column. This is done by the so-called neuron activation functions, and the most common are: Sigmoid (also called Logistic)^119,120^, Hyperbolic Tangent^120^ and Rectified Linear Unit (ReLU)^120,121^. Also, for the particular case of pattern classification tasks, the output values of the VMM performed by the last neural layer have the added requirement of being mapped to the [−1, 1] or [0, 1] range as they indicate the probability of the input to belong to each class. To this end, the gap difference between the value of the most active output (column) and the rest needs to be compressed and the differences among the less active outputs, amplified. It must be noted, that although not necessary in the case of neural networks implemented in the software domain, in the case of neural networks based on memristor-VMM cores, the elements of the input vectors to each neural layer must be within a range of analogue voltages. For this reason, ReLU activation functions, which are by definition unbounded activation functions [0, ∞), needs to be slightly modified with an upper limit to prevent the alteration of the synaptic weights recorded in the neural layer memristors.

**Fig. 9:**
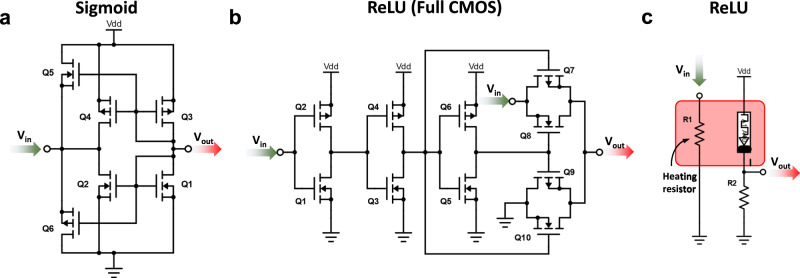
Circuital implementations of the analogue activation functions used in memristive neural networks. Full-CMOS implementations of the **a** sigmoid and **b** ReLU activation functions. Aiming to minimize the area footprint of the activation function, **c** presents a ReLU implementation based on a VO_2_ Mott insulator device.

All these activation functions could be realized either in software or hardware, and each implementation has its own virtues and drawbacks. In this study, software-based implementations refer to the designs, where the calculation of the activation functions and processing of intermediate outputs between ANN layers is performed in a separate hardware unit outside the crossbar. This hardware unit can be an CPU, FPGA, microcontroller, microprocessor or printed circuit board (PCB) depending on how crossbar architecture is integrated with the other processing units. Hardware-based implementations refer to the integration of the memristive crossbars and activation function units into the same chip. In software based implementations, the output of each crossbar column needs to be converted to the digital domain using an ADC (which remarkably increases the area and power consumption) and then sent for the further processing. This is the most commonly used approach on research prototypes developed as technology demonstrators due to its versatility, as the activation function can be implemented and changed by simply modifying the software code^55,100,113–115,122,123^. In the context of future product development, reconfigurable ASICs are proposed for post analogue-digital signal processing. Conversely, hardware ASIC-based implementations of activation functions integrated into the same chip as a crossbar cannot be changed once the circuit is fabricated. Such activation functions can be implemented in both digital and analogue domains. Digital domain processing leads to the ADC overhead (same as for software-based implementations) but is less affected by the noise and transistor mismatches. Digital domain implementation of a ReLU activation integrated into the sensing circuit is shown in^124,125^. In general, analogue CMOS implementations of the activation functions require a smaller number of transistors and help to avoid analogue to digital conversion at this stage. Analogue CMOS implementations of the activation functions are shown in Fig. 9 (see Fig. 9a for the Sigmoid activation function and Fig. 9b for the ReLU activation function). Even though such designs cannot be reconfigured when fabricated, this weakness is compensated by a much reduced power consumption (estimated in ref. ^102^ for a 65 nm CMOS node to be roughly 30 times lower). References ^119,126^, presented analogue CMOS implementations of Sigmoid, ReLU and Hyperbolic Tangent activation functions within ANNs and Generative Adversarial Networks (GAN), respectively.

Since ANNs need to have a very large number of activations to achieve high accuracy, the reduced power consumption of such custom-made analogue CMOS activation functions could still be excessive. Using a compact and energy-efficient nano device implementing the non-linear activation functions could further advance the performance and integration density of memristive ANNs. Reference ^121^ proposed the use of a vanadium dioxide (VO_2_) Mott insulator device (which is heated up by joule power dissipation) to achieve the desired ReLU function (see Fig. 9c), and reference ^127^ proposed the use of a periodically-poled thin-film lithium niobate nanophotonic waveguide to implement this function in optical ANNs. Even though such designs are promising as a small energy-efficient solution for implementing the activation functions, their efficient integration with the other peripheral circuits and CMOS components is still an open challenge.

### SoftArgMax function (Block 8)

Instead of the activation functions previously described, the final synaptic layer in an ANN as those here covered, uses a different block. In this case it is necessary to have a block that detects which is the most active output of the crossbar (i.e., which column drives the highest current). This block (often named SoftArgMax function or SoftArgMax activation function) with as many inputs as bitlines has the memristor crossbar, basically implements Eq. 10:

10 $${y}_{i}=\mathop{{{{{{\rm{arg}}}}}}\, {{{{{\rm{max}}}}}}}_{{z}_{{{{{{\rm{i}}}}}}}\in {{{{{\bf{Z}}}}}}}\left[{{{{{\rm{softmax}}}}}}({{{{{\rm{z}}}}}})_{{{{{{\rm{i}}}}}}}\right]$$

which indicates that the *i*^*th*^ element of the vector **Z** is the maximum among all the elements of **Z**, and thereby identifies the input pattern as a member of class *i*. The input vector **Z** represents the crossbar outputs. This behaviour is achieved by combining two functions, the argmax() and the softmax() functions, shown in Eqs. 11 and 12, respectively.

11 $$\mathop{{{{{{\rm{arg}}}}}}\,{{{{{\rm{max}}}}}}}_{{z}_{{{{{{\rm{i}}}}}}}\in {{{{{\bf{Z}}}}}}}({z}_{i})\,{{ :=}}\,\left\{i/{z}_{j}\le {z}_{i}\forall \,1 < \,j \, < \,K\right\}$$

12 $${{{{{\rm{softmax}}}}}}{\left(z\right)}_{{{{{{\rm{i}}}}}}}=\frac{{e}^{{z}_{{{{{{\rm{i}}}}}}}}}{\mathop{\sum }\limits_{j=1}^{K}{e}^{{z}_{{{{{{\rm{j}}}}}}}}}$$

**Fig. 10:**
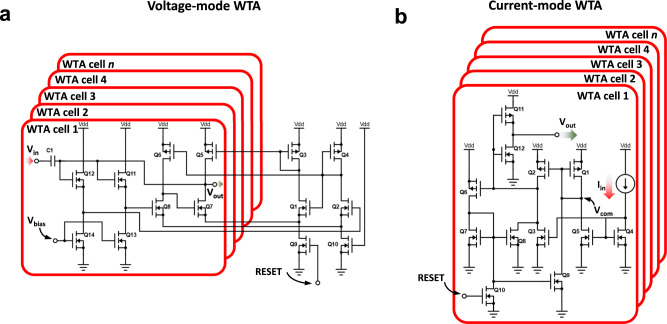
Analogue CMOS implementation of the Winner-Takes-All (WTA) function. **a** WTA CMOS block with voltage input^291^. The gate terminal of transistor Q5, and the source terminals of transistors Q6 and Q7 are common to all WTA cells. **b** WTA CMOS block with current input^148^. Node *V*_com_ is common to all WTA cells. In both cases, the output voltage of the WTA cell with the highest input voltage/current is driven to the positive reference voltage (*V*_DD_), while the output voltage of the remaining WTA cells is driven to ground. The number of cells in the WTA module is the same to the number of classes of images to identify by the ANN.

It could be argued that such a behaviour (i.e. identifying the largest output of the network) could be achieved directly by the argmax() function without the need of the softmax() operation. This is because as indicated in Eq. 11, argmax() is an operation that finds the argument that gives the maximum value from a target function. So, for inference-only accelerators it is acceptable to fed the output of the activation functions directly to the argmax() function, omitting the softmax() function. Some studies proposed to implement the argmax() function via hardware^128–148^, which could be beneficial to reduce the total transistor count and power consumption while at the same time increasing the throughput. In this regard, there are two possibilities: to use of a CMOS digital block^128–131^, or to use a CMOS analogue block^132–148^, which can either operate with a current or voltage input (see Fig. 10a, b, respectively). Note that this blocks in fact implement the so-called winner-takes-all function, widely used in SNNs and particularly in unsupervised competitive learning (this could be regarded as similar to the argmax() function but with the addition of lateral inhibition). The use of a digital block is simpler and more robust (it can be easily written in Verilog or VHDL), but it presents the big drawback of requiring an ADC at each output (i.e., column) of the crossbar.

Yet, it is recommended (even for inference-only) to consider the softmax() function as well, as it turns the vector formed by the output of the activation functions to a vector of probabilities, where the probabilities of each value are proportional to the relative scale of each value in the vector (the summatory of the probabilities of all elements is equal to 1). Note that the *i*th output of the softmax() function is determined not only by the value (*z*) *i*th input but also by the value of the other *j*^*th*^ inputs. Furthermore, for training-capable accelerators, it is usually not possible to omit the softmax() function, as it is required for calculating the loss function, which determines the way in which the synaptic connections are adjusted. This process is done by backpropagating the gradient of each mathematical function of the network, to the previous layer (the details of these procedure will be further described in section ANN training and synaptic weight update (Blocks 2, 11-15): Learning algorithm). Since the gradient of the argmax() function is always zero, its usage without the softmax() function would result in no update of the synaptic weights. Most studies implement this block via software^54,57,85^, which uses a digitalized representation of the voltage signal provided by the preceding activation function (discussed in section Activation function (Block 7)). This approach requires the use of an ADC at the output of the activation function for each column (analogue hardware). This digitized vector is read by a Python^57^ or MATLAB^54^ routine running on a PC or FPGA^85^ and the highest valued element is identified. Although these examples are essentially proofs-of-concept focusing on the hardware implementation of ANNs, it could be argued that future systems-on-chip including both in-memory-computing tiles and conventional Von Neumann cores could rely on the latter ones for implementing functions such as softargmax() function on the digitized vector provided by the in-memory-computing tiles^57^. Note that in some cases, the activation function is also implemented digitally and thereby the ADC block is placed right after the sensing electronics discussed in section Sensing electronics (Block 6).

### Analogue to digital converters (Block 9)

In the cases in which ADCs are needed (either between the output of the crossbar and the activation function block or between the activation function block and the softargmax() block), the most important metrics to consider are: (i) their resolution (as it affects the accuracy), (ii) sampling frequency (*f*_s_) (affects throughput or in other words, the number of operations per second), and iii) surface area on the die (limits the available silicon area to be destined to synaptic weights, that is the 1T1R structures, which thus affects cost).

The resolution of ADC required to represent all possible outputs of the VMM operation depends on input precision $K$ (DAC resolution), number of crossbar rows $N$, and precision of the weights cells $M$ (conductance resolution), and can be calculated as ${{{{{\rm{ceil}}}}}}({\log }_{2}(({2}^{K}-1)* ({2}^{M}-1)*N))$^96^. For example, 1-bit memristors (binary weights) and binary inputs (1-bit) in a 256 × *m* crossbar requires at least a resolution of 8-bit to discriminate all output levels. 5-bit memristors with the same vector dimension and binary inputs require a 13-bit ADC, which represents a serious design challenge to preserve energy consumption/area efficiency and thereby requires a careful cost and overhead analysis^149^ since all these metrics are strongly linked. For instance, based on refs. ^150–152^, increasing 1-bit resolution or increasing the throughput by doubling the sampling frequency results in a 4× increase in power consumption (particularly for highly scaled CMOS technology nodes, where the power consumption is usually bounded by the thermal noise^153^). Similarly, cutting the power consumption by half or adding 1-bit resolution comes at the expense of 25% more silicon area. Moreover, ADC can consume up to 70–90% of the on-chip area of the crossbar-based computation unit, including memristive crossbar and peripheral circuits, and up to 80-88% of energy^55,154,155^. In summary, ADCs are commonly the largest and most power-hungry circuit block in a memristive neural network^55,156^. For these reasons, many authors focusing on the optimization of the 1T1R memory cell structures have opted for using off-the-shelf integrated circuits, assembled in printed circuit boards^54,85^, as in this way they can avoid the limitations posed by the trade-offs between resolution, area and power of the ADCs. Nonetheless, for full on-chip integration of memristive neural network, the impact of ADC resolution on VMM accuracy needs to be carefully evaluated to identify the lowest ADC resolution (and thereby required Silicon area) while preserving the neural network accuracy^91,92^.

**Fig. 11:**
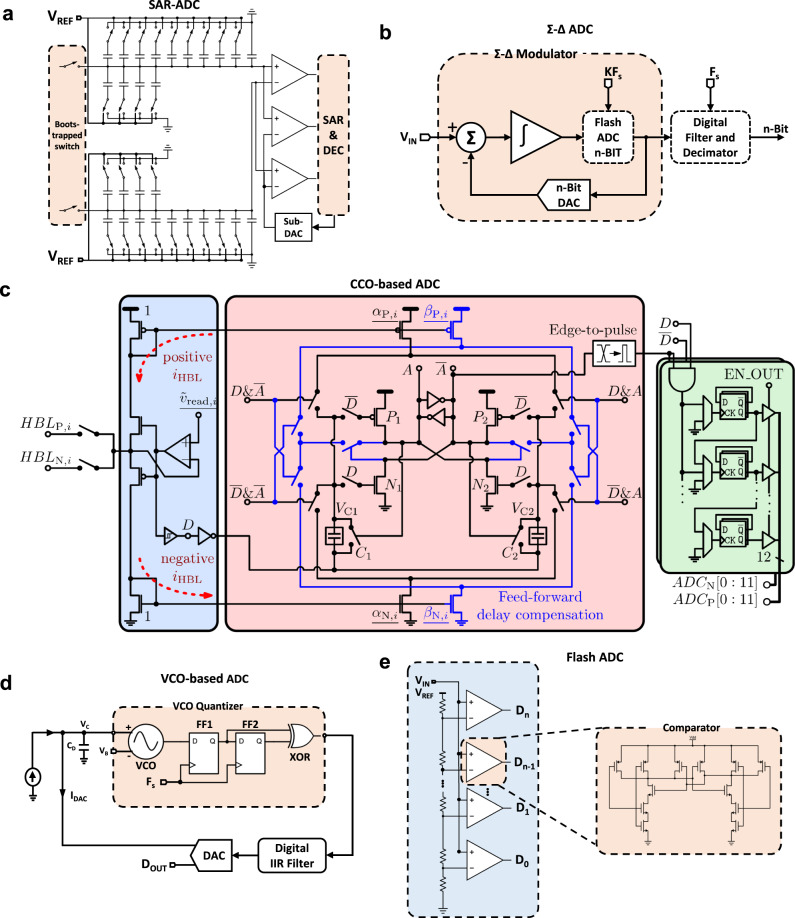
Schematic diagrams of ADC circuits conventionally used in the literature. **a** SAR-ADC, **b** ΔΣ-ADC, **c** CCO-ADC, **d** VCO-based ADC and **e** Flash ADC.

Overall, the choice of ADC architecture depends on the needs of the application and proper system-level design can be very helpful to identify the required ADC performance. As a rule of thumb, ADCs with higher resolutions are slower and less power efficient, whereas the ADCs with a higher sampling frequency have worse energy efficiency and lower resolution. Thereby, if the focus is set on achieving high-resolution (>10-bit) successive approximation register (SAR-ADC, Fig. 11a) or delta-sigma (ΔΣ-ADC, Fig. 11b) can be utilized as they have small form factors and the best signal-to-noise and distortion ratio (SNDR). Furthermore, SAR-ADC and controlled oscillator-based ADCs (Current-Controlled-Oscillators -CCO, see Fig. 11c- and Voltage-Controlled-Oscillators -VCO, Fig. 11d-) are more suitable to smaller technology node implementations^95,117^. In this regard, and unlike the more commonly used VCO-based ADCs, CCO-based ADCs such as the one proposed by Khaddam-Aljameh et al.^71^ (see Fig. 11c) eliminate the need for additional conversion cycles and are amenable to trading off precision with latency. As such, this approach facilitates having one converter per column of the crossbar, thus minimizing the overall latency as no resource sharing will be required. On the contrary, if the focus is set on the sampling frequency (with reading times in the order of 10 ns), low-resolution/high-speed-flash ADC (Fig. 11e) can be applied via time multiplexing to minimize die area as for instance ADCs with at least 8-bit resolution are necessary to achieve high (>90%) classification accuracy in a ResNET50-1.5 ANN used to classify the ImageNET^157^ database or in a multi-layer perceptron to classify the breast cancer screening database^57^. This approach requires the use of analogue multiplexers (block 11).

In general, the reduction of ADC overhead is one of the main challenges in memristor-based ANN hardware design. One way to address this problem is approximate computation or using lower precision ADCs than required^96,158^. The other method is sharing a single ADC across several columns or using a single ADC per crossbar tile^159,160^. However, ADC sharing requires additional multiplexers and sample-and-hold circuits and also increases latency^96^ (i.e. more time is required to process each input pattern, thus reducing the throughput of the ANN). In binarized networks, ADC can be replaced by a 1-bit comparator^96^ or ADC-like multi-level sense amplifier^158^.

Having introduced the interplay between crossbar size, input vector resolution, memristor’s available levels and ADC resolution, and how the ADC resolution impacts the Silicon area, it is worth discussing how these set a constraint for how the memristive ANN will handle input vectors with bipolar (positive and negative) elements. The obvious approach i) is to design the DAC circuits with the capability of providing both positive and negative voltages^161^. This means doubling the number of DAC output levels, and thereby increasing the DAC resolution in 1 bit (with the associated increase in the Silicon area cost as explained in Section Input driving circuits (Block 4)). Nourazar et al suggest in^162^ the use of an analogue inverter with low output impedance which is alternatively connected to the DAC output or bypassed based on the sign bit. Nonetheless, increasing the input DACs resolution by 1 bit, also means increasing the output ADCs by 1 bit, as the number of levels to be distinguished doubles. Therefore, not only the system becomes more sensitive and error-prone, but also its power consumption increases exponentially as the resolution of DACs and ADCs increase^163,164^. An alternative to avoid the Silicon area and power consumption is to apply the positive and negative inputs in two separate read phases with unipolar voltages and subtracting the resulting ADC outputs via digital post-processing. This is similar to what the platform ISAAC^160^ does, which provides 16-bit signed data to the crossbar in 16 cycles (one bit per cycle) in 2’s complement format. Despite being an appealing solution from the cost side, this approach comes with an inevitable reduction of throughput as at least two separate read phase must be employed to complete a single VMM product.

### ANN training and synaptic weight update (Blocks 2, 11-15)

Apart from driving the input and output signals, to perform a fruitful VMM operation, it is fundamental to set the conductance of the memristors in the crossbars to the required values. In the context of ANNs, the process of determining such values is called training or learning, and it can be classified based on i) the nature of the training algorithm, and on ii) how the selected algorithm is implemented. First, regarding the nature of the training algorithm, the typical method of choice for classification problems (as the example discussed here) is supervised learning. Supervised learning is a machine learning approach that is defined by the use of labelled datasets, i.e., the training and test data are paired with the correct label. For the MNIST dataset, this means that an image displaying the number ‘9’ is paired with a tag with the value ‘9’. By using labelled inputs and outputs, the model can measure its accuracy and learn over time. Other learning approaches include unsupervised learning^165^, semi-supervised learning, adversarial learning and reinforcement learning, but their hardware implementation is much more complex. Note that most of the literature claiming unsupervised learning with memristive devices used software^166^, and we are only aware of a few works^53,116,167^, that demonstrated hardware-based unsupervised learning. Second, and concerning how the learning algorithm is implemented, this could be done ex situ, that is, using an idealized model of the network written in software (blocks 2, 11-14) and writing the synaptic weights to the conductances once the training is finished or in situ, that is, using the memristive ANN to compute the VMM operations (blocks 12-15) and progressively updating the concuctance values during the training process. In the following sub-sections the basics of the supervised learning, the difference between ex-situ and in-situ training and the procedure to tune the memristor conductance will be further discussed.

#### Learning algorithm

**Fig. 12:**
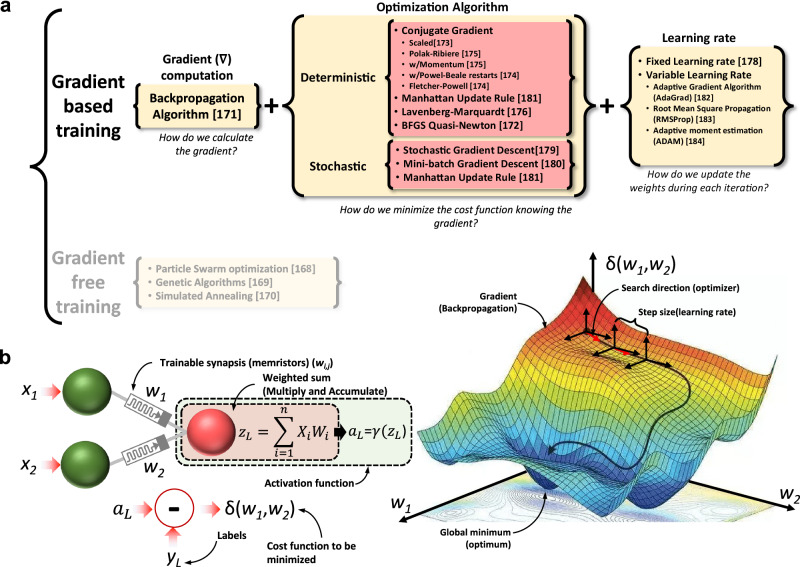
Basic concepts of neural network training. **a** Simplified organization of the most common terms reported in the literature, differentiating between gradient based and gradient free training tools. For the gradient-based tools, we propose an organization of the algorithms for (i) gradient computation, (ii) optimization and (iii) learning rate. **b** Illustration of the gradient descent method, for a trivial 2 × 1 neural network trained with supervised learning.

During the supervised learning, we compute the output of the ANN when presenting an input vector from the training dataset. Such output is then compared against the label associated to the input vector to determine the network’s error. For the case of ANN with *n*^2^ inputs, *m* outputs and no hidden layers, such error is a function of the *n*^2^*m* synaptic weights of the network (${{\mathbb{R}}}^{{n}^{2}m}{\mathbb{\to }}{\mathbb{R}}$), often called loss function. In order to reduce the error, the synaptic weights are updated periodically after a number *z* of input vectors (images) are presented to the network. Then, the learning procedure can be understood as a multivariate optimization problem, where the synaptic weights must be adjusted to values that minimize the loss function. To achieve this goal two families of algorithms could be employed: gradient-free and gradient-based algorithms (as shown in Fig. 12a). Gradient-free methods such as the Particle Swarm optimization^168^, Genetic Algorithms^169^ and Simulated Annealing^170^ algorithms are more demanding from a computational point of view, and hence, they are rarely employed for ANN training, by which they lie beyond the scope of this article.

To understand the basics of the gradient-based algorithms, let us consider an example in which the loss function is a convex bivariate-function, which describes the error of the output (against the labels) for a small network with only two inputs and one output (and thereby 2 synaptic weights, as presented in Fig. 12b), that is ${{\mathbb{R}}}^{2}{\mathbb{\to }}{\mathbb{R}}$. The gradient for such a function indicates, for a random point *x*_1_ *=* (*w*_1_*,w*_2_), the direction in which the loss increases. Using the information provided by the gradient, we can take a step by advancing contrary to the gradient to a new point *x*_2_ *=* (*w’*_1_*,w’*_2_) and expect a lower loss. We can then repeat the same action and make a further step in the direction opposite to the gradient for the point *x*_2_ and reach a new point *x*_3_ *=* (*w”*_1_*,w”*_2_). Such a process will continue iteratively until ideally finding that the gradient is 0, or at least lower than a termination criterion. Within the field of supervised training, each of these iterations is called Epoch. At this point (assuming that we managed to avoid the local minima) we would have found the values for *w*_1_ and *w*_2_ that minimizes the loss function. A frequently used loss function for training ANNs is the cross-entropy loss, which is calculated as follows:

13 $$H=-\mathop{\sum}\limits_{i}{y}_{{{{{{\rm{i}}}}}}}\log \left({p}_{{{{{{\rm{i}}}}}}}\right)$$

were *p*_i_ is the probability of each class for a certain input pattern (calculated with the softmax function), and *y*_i_ is 1 only for the class with the highest probability and 0 otherwise. However, when generalizing these concepts to ${{\mathbb{R}}}^{{n}^{2}m}{\mathbb{\to }}{\mathbb{R}}$, a plethora of challenges and varieties appear, depending on: i) how the required gradient of the loss function is computed, ii) how the loss function is evaluated, iii) how the direction in which to advance is determined, and iv) what is the size of the step in each iteration (among other factors).

In most ANNs, the gradient of the loss function is normally computed by the backpropagation algorithm^171^. Then the evaluation of the loss function could be done deterministically or stochastically. For a deterministic evaluation, all the samples in the train dataset are presented to the network and the loss is computed as the average loss over all the samples. For the stochastic evaluation, the loss is estimated by presenting one single input vector, which introduces a higher degree of variability but speeds up the training process. Alternatively, the use of batches has been also proposed to help reducing the variability, by computing the loss over a batch of input vectors. In other words, under deterministic evaluation of the loss function and considering the MNIST dataset, every Epoch supposes the presentation of 60,000 images. Instead, during stochastic evaluation, every Epoch may consist in presenting 1 image. Note that for the sake of comprehensiveness, and to provide the most complete overview as possible to potential readers who are not already familiar with the field of deep learning, we list both deterministic and stochastic optimization methods. However, deterministic methods are rarely (if ever) used in modern deep learning frameworks, with stochastic optimizers being the de facto standard for the entire community. The reason for this is the high computational burden involved in sending the entire dataset to compute the gradient.

For each case (deterministic/stochastic) there are different algorithms to determine the optimum direction in which search for the minima based on the information provided by the gradient. These are the so-called optimization algorithms. For the case of deterministic evaluation, common optimization algorithms are the following: (i) Gradient Descent^165^ (the simplest one and closest to the previous paragraph’s explanation) and its variants (Gradient Descent with Momentum^165^), (ii) Newton (analytically complex, as besides the gradient it also requires the Hessian matrix associated of the loss function) and Quasi-Newton methods (which operates over an approximation of the Hessian matrix to simplify the problem computation, as the Broyden–Fletcher–Goldfarb–Shanno Quasi-Newton^172^), (iii) Conjugate Gradient methods (an intermediate between the Gradient descent and the Newton methods which avoids the use of the Hessian matrix and instead makes use of the conjugated direction of the gradient, e.g. Scaled Conjugate Gradient^173^, Conjugate Gradient with Powell-Beale restarts^174^, Fletcher-Powell Conjugate Gradients^175^ and Polak-Ribiere Conjugate Gradient^165,175^). Alternatively, other methods are the Levenberg-Marquardt^176^ (uses the Jacobian matrix instead of the Hessian Matrix), Resilient Backpropagation^177^ and One Step Secant^178^, but these are more demanding from a computational point of view. For stochastic evaluation, the most common optimization algorithms are: the i) Stochastic Gradient Descent^179^ (the stochastic equivalent of the Gradient Descent^165^ method previously mentioned, assuming that one epoch consists of only 1 training input vector) and Mini-batch Gradient Descent^180^ (which is a generalization of the stochastic gradient descent method for Epoch sizes greater than 1 and smaller than the entire dataset) and ii) the Manhattan Update Rule^181^ (synaptic weights are updated by increasing or reducing them depending on the gradient direction, but the step is equal for all of them).

The size of the step made in each Epoch to update the synaptic weights is critical because it severely affects the probability of the algorithm to converge, as well as the convergence time, i.e., a large step value will cause the learning not to converge, while small values will result in a sometimes-unacceptable learning time. The simplest approach is to consider a fixed step, although the most advanced learning methods rely in a variable step that is auto-adjusted based on a variety of metrics. In particular, for the case of deterministic evaluation of the loss function the Variable Learning Rate Gradient Descent is often employed^165^, and for stochastic evaluation of the loss function using a mini-batch of images diverse methods have been employed, including Adaptive Gradient Algorithm (or AdaGrad)^182^, Root Mean Square Propagation (or RMSProp)^183^, Adaptive Moment Estimation (or Adam)^184^ and Adadelta^185^.

**Fig. 13:**
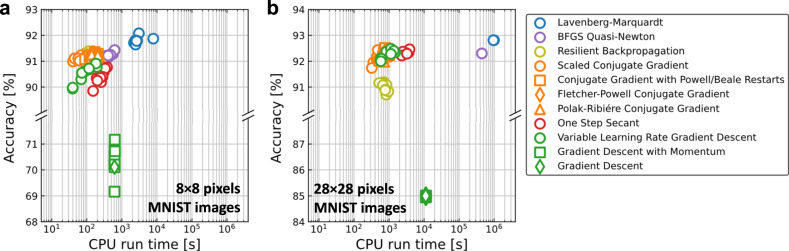
*k*-fold cross validation with 10 repeats considering 11 different learning algorithms. ^165,172–178^ The accuracy obtained in each repeat is plotted against the CPU run-time of the learning algorithm when trained for the MNIST dataset for two different resolutions: **a** 8 × 8 and **b** 28 × 28 px. images. Although the Levenberg-Marquardt algorithm shows the higher mean accuracy, it is also the slowest to converge in our implementation, especially when considering large-size networks, as those required for classifying the 28 × 28 px. images. As a trade-off between accuracy and learning time, we have considered for the example to be described in later in this article, the Scaled Conjugate Gradient, as the accuracy difference with the Levenberg-Marquardt method is not statistically relevant: i.e., the observed difference might be due to a data fluctuation in the test dataset.

Each training algorithm has different mathematical characteristics, which can severely change the accuracy and computing time. For this reason, before employing any of them to compute the 60,000 images of the MNIST dataset, we conduct a small test (called k-fold cross validation) in which a small number of training images and the accuracy depending on the training algorithm is recorded. As an example, Supplementary Algorithm 2 shows the detailed MATLAB code used for this k-fold cross validation using 100 images. The small number of training images is partitioned into *k* groups: *k*-1 groups are effectively used to train the network, while the remaining group is used to validate the training results. Then, this process is repeated *r* times, in each of them using a new set of *k* groups formed by the same small group of images (100 in this example) but shuffled in each repetition. The idea behind this approach is to check whether the trained accuracy depends on the set of data used for the training or not. In this example we divided the 100 images in 5 groups (*k*=5), leading to 80 images for training and 20 for validation (which are different in each repetition), and the accuracy of the ANN was recorded for every repetition (*r*=10 in this example) for each training algorithm. For brevity, we considered only the algorithms for the deterministic evaluation of the cost function provided in the MATLAB Deep Learning toolbox. This implied in total 110 trainings for the 100 images. The result of these tests are reported in Fig. 13a, b, which shows that the Scaled Conjugate Gradient and the Levenberg-Marquardt learning algorithms^176^ provide the highest accuracy; however, the first one is much faster, and for this reason it is the one selected for this example. It is also clear from Fig. 13a, that apart from a lower accuracy, the accuracy obtained with Gradient Descent with Momentum is highly dependent on the training and testing datasets. Further details concerning each training algorithm lie beyond the scope of this article, as we focus on the crossbar-based implementation of the ANN.

After the validation, the real training using the 60,000 training images and the 10,000 testing images is conducted using the Scaled Conjugate Gradient algorithm. The MATLAB code employed to train an ANN containing one 64 × 10 Single Layer Perceptron (SLP) ANN —using MNIST images downsized to 8 × 8— is shown in Supplementary Algorithm 3; the code depicts both the ANN creation and training. The quality of the training process can be evaluated through different figures-of-merit (see definitions in Table 2), which can also be used to define a stopping point for the training procedure. This is critical since if too few iterations are considered during the training phase, the ANN may underfit the training data, and do not properly recognize the input patterns (even during the training phase). On the contrary, excessively training the ANN results in an overfitting of the training data, which although accurately recognizing the training images, reduces the ability of the ANN to correctly recognize unseen input patterns (used during the testing phase).

**Table 2:** List of metrics used for the evaluation of ANNs used for pattern classification

Metric	Expression	Meaning	Applicability	Examples
Accuracy	$\frac{{TP}}{{Total}}$	The ratio of correctly classified patterns respect to the total number of patterns	To quantify the performance of the ANN	N/A
Sensitivity (also called recall)	$\frac{{TP}}{\left({FN}+{TP}\right)}$	Ratio between how much were correctly identified as positive to how much were actually positive	Places where classification of positives are high priority	Security checks in airports
Specificity	$\frac{{TN}}{\left({FP}+{TN}\right)}$	Ratio between how much were correctly classified as negative to how much was actually negative	Places where classification of negatives are high priority	Diagnosing for a health condition before treatment
Precision	$\frac{{TP}}{\left({TP}+{FP}\right)}$	How much were correctly classified as positive out of all positives	N/A	How many of those who we labeled as diabetic are actually diabetic?
F1-score	$2\frac{{precision}*{recall}}{{precision}+{recall}}$	*It is a measure of performance of the model’s classification ability*	N/A	F1 score is considered a better indicator of the classifier’s performance than the regular accuracy measure
Κ-coefficient	$\frac{{Acc}.-{random\; Acc}.}{100-{random\; Acc}.}$	*It shows the ratio between the Network accuracy and the random accuracy (in this case, with 10 output classes, the random accuracy would be 10%)*	N/A	N/A
Cross-Entropy	$\mathop{\sum }\limits_{i=1}^{n}\mathop{\sum }\limits_{j=1}^{m}{y}_{i,j}\log ({p}_{i,j})$ *where, y*_*i,j*_ *is 1 if sample i belongs to class j and 0 otherwise, and p*_*i,j*_ *is the probability predicted by the ANN of sample i belonging to class j*	*Difference between the predicted value by the ANN and the true value*	N/A	N/A

The main metric considered has been the Accuracy, and the others are added for completion. *TP* True Positive, *TN* True Negative, *FP* False Positive, *FN* False Negative.

**Fig. 14:**
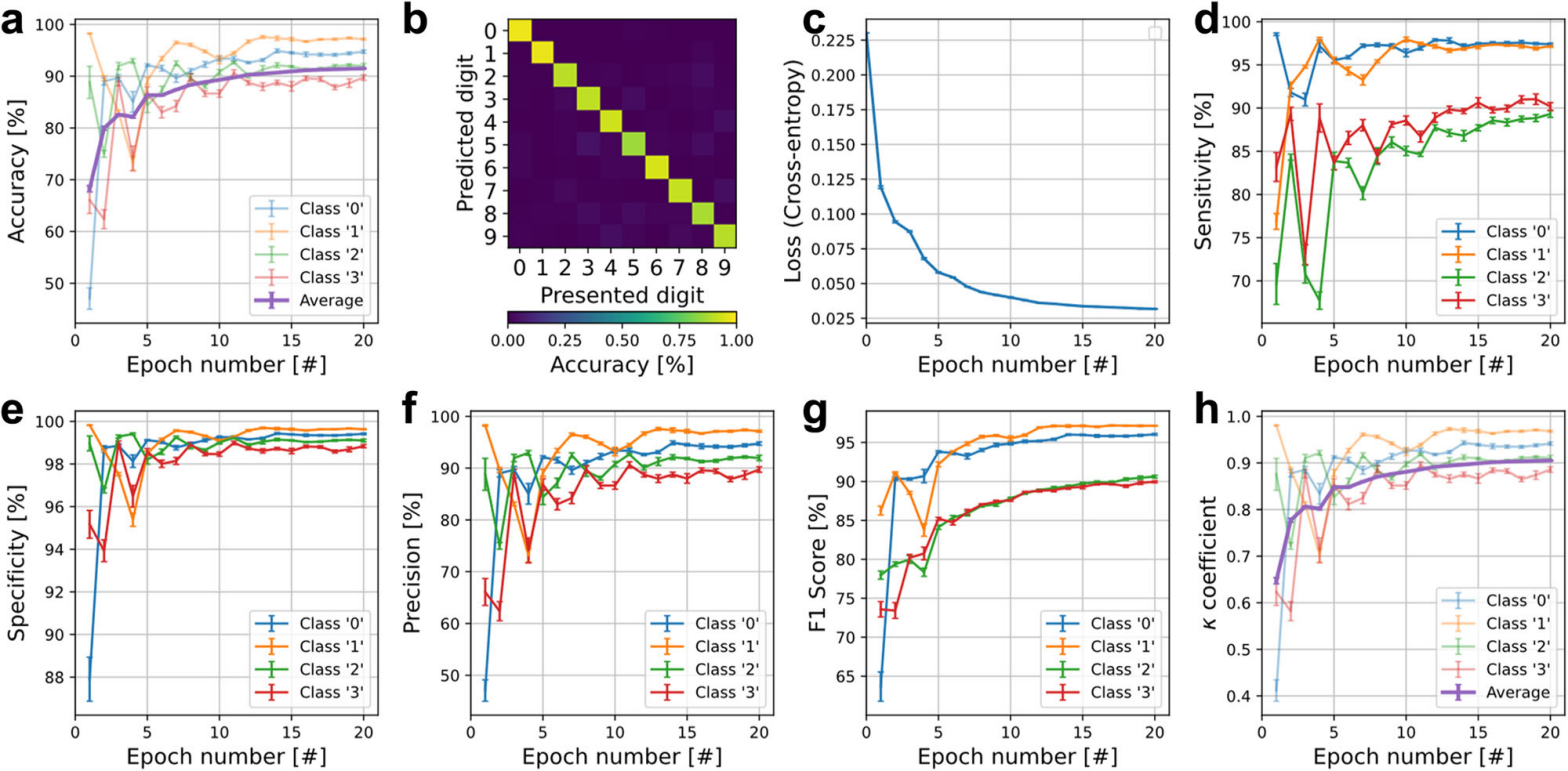
Typical figures-of-merit used to quantify the performance of ANNs intended for pattern recognition. In this case, they are plotted as a function of the training epochs. **a** Accuracy, **b** confusion matrix, **c** Loss function (cross-entropy), **d** Sensitivity, **e** Specificity, **f** Precision, **g** F1-score, **h** κ-coefficient.

As an example, Fig. 14 shows the metrics for the training obtained from Supplementary Algorithm 3. The most popular figure-of-merit is the inference accuracy (see Fig. 14a), that is the ratio between the number of correctly-classified images, respect to the total number of images presented to the ANN in each iteration (often called epoch). Another popular metric is the confusion matrix (see Fig. 14b), which displays the ability of an ANN to associate each input pattern with its corresponding class (in this example a digit from 0 to 9) and allows to graphically represent the inference accuracy for each possible input. Also, the loss function used for training is a critical metric. One of the most commonly employed loss functions is the Cross-Entropy (see Fig. 14c and Table 2), which can be computed as the difference between the predicted value by the ANN and the true value. Last but not least, other relevant metrics include the Sensitivity (Fig. 14d), Specificity (Fig. 14e), Precision (Fig. 14f), F-1 score (Fig. 14g) and κ-coefficient (Fig. 14h), whose definition is presented in Table 2, in terms of the True Positives (TP, images from class *k* classified as members of the class *k*), True Negatives (TN, images which are not members of class *k* and that are not classified as class *k*), False Positive (FP, images that do not belong to class *k* but are classified as class *k*) and False Negatives (FN, images that do belong to class *k*, but are not classified as class *k*). In supervised classification algorithms the cross-entropy metric is used as the loss-function to be minimized during the training phase.

It is important to emphasize that the figures-of-merit generated by the software (MATLAB, Python) code during the training phase until this point have no connection with memristors or crossbar arrays. We note that some articles focused on the fabrication and device-level characterization of one/few memristors^62–70,186,187^, also present some of the figures-of-merit generated by a software-based training ANN process (similar to the ones in Fig. 14) in order to claim that their devices exhibit potential for neuromorphic applications. This is not a recommended practice and should be always avoided, as the models involved in these cases keep little connection with the fabricated devices, leading to unrealistic performance metrics.

#### Ex situ versus In situ training

For ex situ training, the resized *n × n* images are introduced in a software-based ANN with a size *n*^*2*^ *×* m. The software calculates the synaptic weights that minimize the loss function by applying the selected algorithm (described in the previous Subsection), either for a certain number of Epochs or until the loss function is below a given threshold. Then, the synaptic weighs (block 11) are recorded into the memristive crossbar using the Write-Verify approach (block 12-14, described in the following Subsection). Ex situ training has the advantage of requiring little/no circuit overhead to perform quick tests of the classification performance of the network, and has made possible to evaluate the performance of home-made memristive crossbar-arrays^93,188^. Note that in their most simple implementation, the non-idealities of the hardware memristive crossbar notably degrade the accuracy obtained with ex situ trained memristive neural networks. To avoid this loss of accuracy, hardware-aware training methods, in which device non-idealities are incorporated during training have been proposed in the literature^189,190^.

In situ training stores and updates the synaptic weights (block 15) directly in the memristors, and performs computations (for example, forward passes) at the original place where the neural network parameters are stored, which has many advantages. For example, it avoids the need to implement a duplicated system in digital computers, as in ex situ training schemes, which substantially enhances the area/energy efficiency of the system by eliminating the processor-memory bottleneck of digital computers and avoids the mapping process. More importantly, in situ training with backpropagation is capable of self-adaptively adjust the network parameters to minimize the impacts of the inevitable non-idealities of the hardware (such as wire resistance, analogue peripheral asymmetry, non-responsive memristors, conductance drift and variations in the conductance programming) without any prior knowledge of the hardware^54^. However, there are two factors that complexifies the implementation of in situ training. First, devices involved require high resolution to program the weight update accurately and a high endurance due to the frequent SET/RESET operation during training process^191^. Mixed-precision training, which accumulates the weight update in software and only updates the memristor devices when the accumulated value surpasses the programming granularity, can greatly relax requirement for conductance update resolution and endurance and allow software-comparable accuracy to be achieved^192,193^. Second, to fully exploit in situ learning in a practical application, it is necessary not only to perform the VMM in the crossbar, but also to carry out the learning algorithm on-chip. In this regard, the challenge is twofold: On one hand, it has as a pre-requisite a high maturity of the memristor technology involved. This means that the memristor stack must be capable of being safely integrated in the back-end-of-line of the CMOS process without compromising the front-end-of-line. This is already a limitation to many research studies in which the stack involves materials and processes that are unfriendly to the typical CMOS stacks. On the other hand, and provided that the previous condition can be met, the development of the necessary on-chip electronics is not straightforward and supposes a major cost for research programs. As such, the trade-of solution is to have the peripheral circuit electronics implemented off-chip with off-the-shelf components. In this way, the impact of the analogue electronics can be assessed more realistically without incurring into prohibitive expenses, leading to a variety of prototypes in which the circuitry needed for the backpropagation are implemented off-chip, an approach here labelled as partial-in situ. This is the case of refs. ^54,105,194^. In all these works the VMM operation required for the forward pass is performed by the memristor crossbar and the digitalized output vectors recorded by an acquisition printed circuit board. Then the output vector is processed by the training algorithm in software to determine how to update the synaptic weight after each training epoch. Through this partial approach, in situ training of ANN accelerators and feed-forward ANNs were demonstrated from fully-connected neural networks to convolutional neural networks (CNNs), showing improved ability for pattern classification. Despite the learning methods described in the previous Subsection also being valid for in situ training, the usual practice reported in the literature for this kind of training has been the use of the so-called Manhattan Update Rule^105,194^, or the Stochastic Gradient Descent^54^.

#### Weight programming

The weight programming stage is the process by which the conductance (i.e., weights) of the memristors are updated to either map the ex situ trained weights or by following the specific rules of the learning algorithm for in situ approaches. The weight update process is implemented by applying voltage or current pulses to the memristors (block 13 and 14), following the Write-Verify (or Close Loop Tunning)^194–196^, or the Write-without-Verify (or Open Loop Tunning)^103,107,197,198^. The difference between them is that for the write-verify approach a read pulse is applied in between successive write pulses, to measure the conductance achieved after a write pulse and determine whether the weight update has been completed, or more/higher pulses are required. When the conductance of the memristors in the crossbar require a frequent update, the write-without-verify method is the most appropriate because it preserves the high-speed operation and keeps the hardware overhead to a minimum, at the cost of incurring in a higher writing error. On the contrary, if better controllability of the conductance values is preferred over high-speed operation or if a frequent conductance update is not a major requirement, write-verify has been pointed out as the best option.

The processes by which the memristor conductance is increased and decreased are called potentiation and depression, respectively, and have been observed when applying different sequences of voltage pulses^199–204^. They are associated with the modification of one/few properties of the materials in the memristive device (e.g., position of atoms, phase, polarization, spin, etcetera). A plethora of studies have revised the different switching mechanisms of memristive devices^205–212^, therefore we will not further dig into this issue. But the important thing from an ANN point of view is that the conductance change during the potentiation and depression processes is in most cases nonlinear. Introducing nonidentical pulses can help to reduce non-linearity, and some studies reached near-linear and symmetric potentiation and depression process by applying incremental positive pulses and decremental negative pulses, respectively^213^. In the 1T1R architecture, the third terminal (i.e., the gate of the transistor) offers higher controllability in tuning the conductance of the memristor^54^.

However, using a variable pulse scheme usually requires a write-verify approach to first identify the conductance state and then apply the correct pulse scheme to the device, or storing externally the pulse amplitudes to apply to each weight. For this reason, these approaches have been demonstrated mostly for the weight update of isolated devices, with just a few examples of on-chip integrated approaches^214^. Also, both options inevitably increases the complexity of the peripheral circuits as well as the latency and energy likely making the in situ weight update with variable pulse schemes just as inefficient as doing it externally in digital. Thereby, only approaches where identical pulses are applied to devices are used when designing neuromorphic circuits aiming to be energy efficiency. Yet, even the conventional Write-Verify pose a great exigence on the current measuring block, which must be accurate both for measuring the current through a single device (during the weight update phase) as well as through the entire column (during inference). In this regard, a promising new approach has been recently proposed by Büchel et al.^215^, aiming to further optimize the Write-Verify method. In this variant, instead of updating each weight with the goal of reaching a given conductance target, the weights are updated in order to minimize the error of the VMM product. As such, the design requirements for the current measuring circuits are less exigent.

## Fabrication/integration of the ANN chip

Crossbar arrays of two-terminal metal/insulator/metal (MIM) memristive devices can be fabricated easily using standard lithography and deposition techniques; this has been readily achieved by multiple groups^57,64,68–70,85,93,105,216,217^. Some groups prefer to incorporate a transistor in series to each MIM cell to obtain a better control over the currents through the device (i.e., improve conductance controllability and minimize sneak path currents)^53–55,60,61,102,113,167,194,218–222^,). A common practice is to fabricate the transistors in a company and mount the MIM cells on top of the transistors in-house on the as-received wafer (after the removal of the passivation film or native oxide, so that the terminals of the transistor can be reached)^48,57,219^.

The crossbar (block 5 in Fig. 2) is then integrated in the ANN by connecting each one of its inputs to a DAC (block 4, to apply the analogue voltage that represents the brightness or colour of each pixel of the image), and each one of its outputs to a TIA (block 6, to convert the output current into voltage); then, the analogue voltage output of the TIA is feed to the block that implements the activation function (block 7) and softargmax() function (block 8). To fully exploit the advantages of the crossbar array of memristors, the best scenario would be to fully integrate the CMOS blocks (DAC, TIA, ADC) on-chip. However, to avoid slow and expensive microchip fabrication (i.e., tape outs), most groups prefer to build the CMOS blocks off-chip. In the following lines we list the most common strategies followed for the hardware-implementation of memristive ANNs, from the most rudimentary up to the most complex:

The most elementary approach is a sequential (row-by-row) analogue multiplication with binary inputs^194^, which does not perform an analogue VMM operation because, despite the multiplication operation is done in each memristor, the accumulation is performed by external circuitry. Then, analogue VMM has been demonstrated both for binary inputs and weights^218–220^, as well as for binary inputs and analogue/multilevel weights^60,85,105,217^. In both cases, the circuit complexity is slightly reduced by avoiding the use of DACs in the inputs of the crossbar. Advantages specific to each case are for the case of binary weights a simpler and more reliable conductance adjustment, and for analogue/multilevel weights a higher number of bits per synapse. However, in both cases the possible input voltages are only 0 or *V*_READ_, meaning that it can only work with two colours per pixel (i.e., black/white images). The use of analogue/multi-level input signals is beneficial to process images with more colours per pixel, but it sets the requirement of a DAC for each wordline. When the number of levels of the input signal increases, so does it the complexity of the DAC circuit (and with it, its power consumption and area). The most common approach in this contest is the use of an Off-the-shelf, external DAC to drive the analogue inputs^54,55,102,222^, which are integrated with the rest of the circuit (i.e. the memristor crossbar) in printed circuit boards. For truly full-hardware, full-analogue VMM approaches, it is necessary to integrate on the same silicon chip the DAC, ADCs and memristor crossbar. This is usually limited by the area requirements of these two analogue blocks. A cost-effective recurrent solution has been to use a smaller number of DACs and share them among different rows by adding a layer of analogue multiplexors between the DACs and the wordline inputs^93,95,221^. With this approach (which we could refer to as On-chip time-multiplexed analogue input – Analogue/Multilevel weights), a given VMM operation is divided in *n* different sub-VMM operations and the partial results of each of them are added up at the end, saving area and power at the cost of throughput reduction. Finally, the most advanced prototypes exploit the time-encoding scheme, which simplifies the DAC design and allows one DAC per channel, without losing resolution of the input vector^57,71,167^. We label this case as On-chip multi-bit input – Analogue/Multilevel weights*.* In Table 3, we present a brief comparison between the most advanced hybrid RRAM/CMOS ANNs architectures and the Fully-CMOS versions commercially available. As shown, they achieve a similar performance in terms of throughput, but sometimes the hybrid RRAM/CMOS architectures are still limited by the large area consumption of the ADC circuits.

**Table 3:** Performance comparison (throughput -Tera OPerations per Second, TOPS-, Density and Efficiency) between hybrid CMOS/Hybrid prototypes and full-CMOS neuromorphic accelerators

	Exp./Sim	Type	Process (nm)	Activation resolution	Weight resolution	Clock speed	Benchmarked workload	Weight storage	R_high_	R_low_	Array size	ADC type	Throughput (TOPS)	Density (TOPS per mm^2^)	Efficiency (TOPS per W)
NVIDIA T4^277^	Exp.	Full-CMOS	12	8-bit int	8-bit int	2.6 GHz	ResNet-50 (batch = 128)	--	--	--	--	--	22.2, 130 (peak)	0.04, 0.24 (peak)	0.32
Google TPU v1^19^	Exp.	Full-CMOS	28	8-bit int	8-bit int	700 MHz	MLPs, LSTMs, CNNs	--	--	--	--	--	21.4, 92 (peak)	0.06, 0.28 (peak)	2.3 (peak)
Habana Goya HL-1000^278^	Exp.	Full-CMOS	16	16-bit int	16-bit int	2.1 GHz (CPU)	ResNet-50 (batch = 10)	--	--	--	--	--	63.1	--	0.61
DaDianNao^279^	Sim.	Full-CMOS	28	16-bit fixed-pt.	16-bit fixed-pt.	606 MHz	Peak performance	--	--	--	--	--	5.58	0.08	0.35
UNPU^280^	Exp.	Full-CMOS	65	16 bits	1 bit	200 MHz	Peak performance	--	--	--	--	--	7.37	0.46	50.6
Reference mixed-signal^281^	Exp.	Full-CMOS	28	1 bit	1 bit	10 MHz	Binary CNN (CIFAR-10)	--	--	--	--	--	0.478	0.1	532
ISAAC^160^	Exp.	RRAM-CMOS	32	16 bits	16 bits	1.2 GHz	Peak performance	ReRAM (8×2-bit)	~2 M	~2 k	128×128	SAR (8-bit)	41.3	0.48	0.63
Newton^282^	Exp.	RRAM-CMOS	32	16 bits	16 bits	1.2 GHz	Peak performance	ReRAM (8×2-bit)	~2 M	~2 k	128×128	SAR (8-bit)	--	0.68	0.92
PUMA^154^	Exp.	RRAM-CMOS	32	16 bits	16 bits	1.0 GHz	Peak performance	ReRAM (8×2-bit)	1 M	100k	128×128	SAR	26.2	0.29	0.42
PRIME^125^	Sim.	RRAM-CMOS	65	6 bits	8 bits	3.0 GHz (CPU)	--	ReRAM	20 k	1 k	256×256	Ramp (6-bit)	--	--	--
Memristive Boltzmann machine^283^	Sim.	RRAM-CMOS	22	32 bits	32 bits	3.2 GHz (CPU)	--	ReRAM	1.1 G	315 k	512×512	SAR	--	--	--
3D-aCortex^83^	Exp.	RRAM-CMOS	55	4 bits	4 bits	1.0 GHz	GNMT	NAND flash	--	2.3 M	64×128	Temporal to digital (4-bit)	10.7	0.58	70.4
Analog-AI Using Dense 2-D Mesh^284^	Sim	RRAM-CMOS	14	8 bits	Analogue	1.0 GHz	RNN/LSTM	PCM	No data	No data	512×512	Current controlled oscillator based	376.7	No data	65.6

Adapted from with permission under CC BY 4.0 license from ref. ^276^.

For all cases, the performance (defined in terms of accuracy, operations per second, power consumption, and area requirements) is limited by the electrical characteristics of the memristor devices (non-idealities such as sneak-path effect, noise, line resistance which are further discussed later in the article) and the available CMOS peripheral circuitry. To maximize the achievable performance with a given memristor technology is critical to select adequate peripheral circuits (described in  Section Structure of memristor-based ANNs). Since the design and further tape-out (i.e., fabrication) of custom CMOS ASICs is time-consuming and expensive, it is imperative to keep the number of design-fabrication-measurement cycles to a minimum. To meet this goal, chip designers rely on simulators, which are capable of providing an estimation of the integrated circuit performance and even spot possible design troubles even before the tape-out phase.

## Simulation of memristive ANNs

**Fig. 15:**
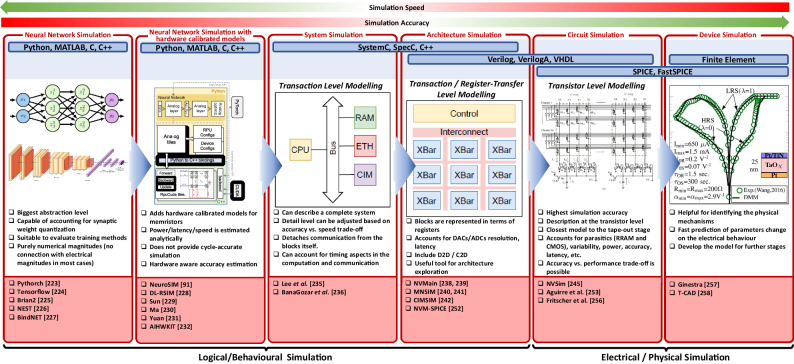
Schematic representation of the trade-off between simulation speed and accuracy across the different tools reported in the literature for memristive ANNs evaluation. For each case, we list the main programming languages involved and some examples.

Simulators are an essential tool used from low-level device modelling to high-level system exploration. Figure 15 illustrates the five major abstraction levels on which simulations are used, whereas Table 4 presents a comprehensive list of the software considered in the literature for ANN and memristive ANN simulation. In general, the trade-offs between the simulation speed and the accuracy (i.e., how close the electrical simulation resembles the real measurements of the circuit) of the simulated results have to be considered. On one hand, simulations on the neural network level require a high performance due to the vast amount of operations (e.g., VMM, pattern flattening, activation functions) and, hence, it is not optimized in terms of simulation accuracy. On the other hand, simulations conducted on the device level have to compute accurate physical models to mimic the behaviour of the devices, which slows down the simulation speed. In the following paragraphs we briefly summarize some of the main simulators developed ad hoc for the simulation of ANNs at different abstraction levels.

**Table 4:** Summary of reported simulation frameworks for the study of memristive hardware neural networks

Simulation Framework	Year	Platform	Training	Simulation type	Open Source	Type of ANN	Compatible dev.	Energy	Accuracy	Power	Latency	Variability	R_L_	C_L_	CMOS	GPU
Tensorflow^224^	2015	Python	Yes	Neural network	Yes	MLP, CNN	No dev.	No	Yes	No	No	Yes	No	No	No	Yes
Pytorch^223^	2017	Python	Yes	Neural network	Yes	MLP, CNN	No dev.	No	Yes	No	No	Yes	No	No	No	Yes
NEURON^285^	2006	Python	Yes	Neural network	Yes	SNN	No dev.	No	Yes	No	No	Yes	No	No	No	Yes
Brian2^225^	2019	Python	Yes	Neural network	Yes	SNN	No dev.	No	Yes	No	No	Yes	No	No	No	Yes
NEST^226^	2007	Python	Yes	Neural network	Yes	SNN	No dev.	No	Yes	No	No	Yes	No	No	No	Yes
BindsNET^227^	2018	Python	Yes	Neural network	Yes	SNN	No dev.	No	Yes	No	No	Yes	No	No	No	Yes
Memtorch^286^	2020	Python, C++, CUDA	No	Neurla network	Yes	CNN	RRAM	No	Yes	No	No	Yes	No	No	No	Yes
NVMain^238,239^	2015	C++	No	Architecture	Yes	Memory	RRAM	Yes	No	Yes	Yes	No	No	No	No	No
PUMA^154^	2019	C++	No	Architecture	No	MLP, CNN	RRAM	Yes	Yes	Yes	Yes	No	No	No	Yes	Yes
RAPIDNN^247^	2018	C++	No	Architecture	No	MLP, CNN	RRAM	Yes	Yes	Yes	Yes	Yes	No	No	Yes	No
DL-RSIM^228^	2018	Python	No	Architecture	No	MLP, CNN	RRAM	No	Yes	No	No	Yes	No	No	No	Yes
PipeLayer^246^	2017	C++	Yes	Architecture	No	CNN	RRAM	Yes		Yes	Yes	No	No	No	No	No
Tiny but Accurate^230^	2019	MATLAB	No	Architecture	Yes	CNN, ResNET	RRAM	Yes	Yes	Yes	No	No	No	No	No	No
Yuan et al.^231^	2019	C++, MATLAB	Yes	Architecture	Yes	No data	RRAM	No	Yes	Yes	No	No	No	No	No	Yes
Sun et al.^229^	2019	Python	Yes	Architecture	No	MLP	PCM, STT-RAM, ReRAM, SRAM, FeFET	Yes	Yes	Yes	No	Yes	No	No	No	No
A. Chen^248^	2013	MATLAB	No	Circuit	Yes	MLP	RRAM	No	No	Yes	No	Yes	Yes	No	No	No
CIM-SIM^242^	2019	SystemC (C++)	No	Architecture	Yes	SLP	RRAM	No	No	No	No	No	No	No	No	No
MNSIM^240,241^	2018	Python	No	Architecture	Yes	CNN	RRAM	Yes	No	Yes	Yes	Yes	No	No	No	Yes
NVSIM^245^	2012	C++	No	Circuital	Yes	Memory	PCM, STT-RAM, ReRAM, Flash	Yes	No	Yes	Yes	No	No	No	No	No
CrossSIM^287^	2017	Python	No data	Circuital	Yes	No data	PCM, ReRAM, Flash	No	Yes	No	No	Yes	Yes	No	No	Yes
NeuroSIM^91^	2022	Python, C++	Yes	Circuital	Yes	MLP, CNN	PCM, STT-RAM, ReRAM, SRAM, FeFET	Yes	Yes	Yes	No	Yes	No	No	No	Yes
NVM-SPICE^252^	2012	Not specified	No	Circuital	No	SLP	RRAM	Yes	No	Yes	Yes	Yes	No	No	Yes	No
IBM Analog Hardware Acceleration Kit^232^	2021	Python, C++, CUDA	Yes	Neural network	Yes	MLP, CNN, LSTM	PCM	No	Yes	No	No	Yes	No	No	No	Yes
Fritscher et al.^256^	2019	Mixed (VHDL, Verilog, SPICE)	No	Circuital	No	MLP	PCM, STT-RAM, ReRAM, SRAM, FeFET	Yes	Yes	Yes	Yes	Yes	Yes	Yes	Yes	Yes
Aguirre et al.^253^	2020	Mixed (Python, MATLAB, SPICE)	No	Circuital	No	MLP	PCM, STT-RAM, ReRAM, SRAM, FeFET	Yes	Yes	Yes	Yes	Yes	Yes	Yes	Yes	Yes

### Neural Network level simulation

The highest abstraction level in neural network simulation is comprised by the conventional machine learning tools such as the open source PyTorch^223^ (originally developed by Meta AI) and TensorFlow^224^ (proposed at Google Brain) frameworks, widely used in computer vision and natural language processing. Both are Python libraries highly optimized to exploit GPUs and CPUs for deep learning tasks. These simulators allow training and developing complex neural network architectures (e.g., CNNs architectures such as the VGG and AlexNET or Recurrent Neural Networks - RNN). Although extremely popular, these simulators provide no link at all with memristive or CMOS devices, as in both cases the magnitudes involved are non-dimensional and the synaptic connections are represented by loosely constrained numerical values.

A common workaround to partially solve these limitations, particularly for the case of Spiking Neural Networks (a particular kind of ANNs where the input vector is codified in terms of firing rate or timing instead of voltage amplitudes), has been the use of biology-oriented simulators. Among them, Brian2^225^ written in Python can be easily executed on a CPU or GPU while implementing a wide variety of neurons, input encoding methods and several learning methods such as Spike-Timing Dependent Plasticity (STDP). Taken all this into account and considering that the focus of Brian2 is on flexibility and ease of use rather than performance, it only supports simulations running on a single machine. An alternative simulator that maintains all these features while also providing support for distributed simulations across a cluster is the NEST simulator^226^. Another alternative to Brian2 capable of providing better performance at the cost of a lower fidelity to the real biological model is the BindsNET simulator^227^, a Python library built on top of PyTorch^223^. Apart from supporting CPU/GPU operation and accounting for a wide variety of neurons, input encoding methods and several learning methods (such as STDP), BindNET can be used on multiple hardware platforms like: ASIC, FPGA, Digital Signal Processing (DSP) or Advanced RISC Machine (ARM) based platforms.

Another interesting approach proposed in the literature is the addition of custom modules into the TensorFlow or PyTorch neural network models, which are responsible of capturing the non-idealities induced by the use of memristors. This approach could be treated as a sub-category within this group, which accounts for hardware calibrated device models. Whitin this group, we found for instance the DL-RSIM simulator, proposed by Lin et al.^228^, which simulates the error rates of every sum-of-products computation in memristor-based accelerators externally, and injects the errors in targeted TensorFlow-based neural network models. The same philosophy was adopted by Sun et al.^229^, placing special emphasis on the effect of the non-linear and quantized nature of the synaptic weight update. Since both cases consider TensorFlow for the simulator implementation, they offer support for pre-trained DNN conversion, GPU-accelerated inference and parameter mapping. However, the negative side is that these are rather closed pieces of software, which has been partially solved by Ma et al.^230^ and Yuan et al.^231^, by using PyTorch instead of TensorFlow, focusing in this case on the weight pruning and quantization effects. Also, the IBM Analog Hardware Acceleration Kit proposed by IBM^232^ could be listed within this group. This framework simulates neural networks with hardware-calibrated device models and circuit nonidealities. However, it provides only accuracy estimates using hardware-calibrated noise models and lacks the cycle-accurate simulations of runtime or energy. A final example (although other cases exist) is the NeuroSim^91^. This simulator can account for the characteristics of the memory type, non-ideal device parameters, transistor technology node, network topology, array size and the training dataset by mapping ANN models onto tile resources, and scheduling the full workload execution, from which it reports hardware aware accuracy metrics. Although it also reports other system parameters such as area, latency and dynamic energy consumption these are obtained by analytical estimations and not cycle-accurate simulations. All in all, these toolkits are very useful for an early-stage estimation of the learning accuracy in run-time.

### System-level simulation

The highest abstraction level that keeps some degree of connection with the hardware implementation of the neural network is the System Level simulation, which can be thought as a particular case of Transaction Level Modelling (TLM). In TLM the details of communication among computation components are separated from the physical mechanisms governing those components. Communication is modelled by channels, while transaction requests take place by calling interface functions of these channel models. Unnecessary details of communication and computation are hidden in a TLM and may be added later (see the following Sub-Section Architecture level simulation). This can be greatly exploited when using TLM for top-down approaches that start the design from the system behaviour representing the design’s functionality; then, generate a simplified system architecture from the behaviour, and gradually reaches the implementation model by adding implementation details. It is precisely this capability of customizing the representation detail of the connections and computation cores that enables high throughput performance (always at the cost of decreasing accuracy and the connection with the physical mechanisms governing the response of the memristors). Although not limited to, conventional programming platforms for System Level Simulation/Transaction Level Modelling include SystemC^233^ and SpecC^234^.

Examples of this simulation abstraction level include the work by Lee et al.^235^, which introduced a cycle-accurate system simulator to model hardware-implemented spiking neural networks. These networks follow a hierarchical structure that conceives the computing-in-memory system as an interconnection of neuromorphic cores or tiles, each of these ultimately created by the joint assembly of crossbar modules. The crossbar representation offers the ability of mimicking the non-ideal effects of actual RRAM devices which includes non-linear RRAM effects like stuck-at-faults (SAFs), write variability, and random telegraph noise (RTN). It is worth to remark that to efficiently connect the tiles, a customizable network on chip (NoC) is used, which together with the crossbar module description, allows for high flexibility and configurability.

Compared to ref. ^235^, the simulator of BanaGozar et al.^236^ focuses on the system integration of neuromorphic computing systems. Hence, the authors implemented a micro-instruction set architecture to control and operate the analogue as well as the digital components of the system. In general, the simulator follows a similar hierarchical structure as in ref. ^235^ by implementing computing in memory (CIM) tiles. These tiles are composed of a memristive memory crossbar, analogue/digital converters, digital input modulators and sample and hold stages. Furthermore, each tile has a dedicated controller orchestrating the components responsible for driving the computation.

### Architecture-level simulation

**Fig. 16:**
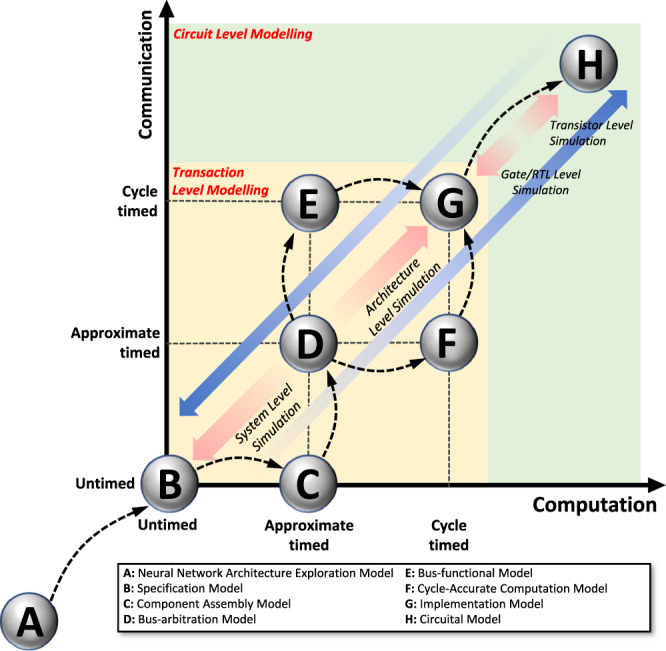
Detail of the different stages of the transaction level modelling, with the addition of the Neural Network and transistor (circuit) level simulation. Modelling approaches are arranged based on how accurately (untimed, approximate, cycle-accurate) the timing of the computation and communication aspects are captured. Transaction level models then expand from B to G, with B being the specification models (which uses considers the communication and computation to be untimed) and G the implementation models (which considers both cycle-accurate timing for both computation and communication). As we approach B, the model can be regarded as a System Level Simulation, while if it approaches G, it is regarded as an architecture-level simulation. Outside this group, we find those models simulated in Python or similar tools which focus on the network topology (A) and the circuital models which materializes the implementation models (G) in the transistor or register transfer level.

Given their customization capabilities, TLM can be divided into different categories as indicated by Gai et al.^237^ (see Fig. 16). Specification models (B) are those with the lowest degree of detail and lie closer to the neural network models (A) described previously. On the opposite corner, the Implementation Models (G) are the step immediately before the Circuital models (H) designed at the transistor level. As TLM approaches the stage of implementation models, they are also referred to as Register-Transfer Level (RTL) Models and embody what is sometimes called Architecture-Level Simulation. In other words, Architecture-Level Simulation can be considered as a sub-type of TLM with a higher detail regarding the communication and computation interfaces. Also, as the detail level increases, the programming language migrates from SpecC and systemC (used for system-level simulations) to Hardware-Description related languages, such as Verilog, Verilog-A or HDL, and even a combination of programming languages such as C++, CUDA, MATLAB and Python to simulate the behaviour of memristive devices during inference.

Emerging non-volatile memory simulators NVMain^238^ (and its successor, NVMain 2.0^239^) were proposed by Poremba et al., as an example of architecture-level, highly flexible, user-friendly main memory simulators. Although NVMain 2.0 allows to estimate energy consumption metrics based on the results of circuit-level simulations, it has limitations. Since it focuses on memory-oriented simulations of emerging non-volatile structures it does not support the inclusion of the peripheral circuitry that would be necessary to model compute-in-memory architectures. To overcome this challenge, Xia et al.^240^ presented MNSIM and Zhu et al. presented the successor MNSIM 2.0^241^. The simulator uses a behavioural model to estimate the worst case and average accuracy which significantly improves the performance of the simulation. Since memristive devices show a non-linear I-V characteristic, the behavioural model interpolates the physical characteristic with a linear function to reduce the computational effort. As a result, the performance is increased. MNSIM^240,241^, proposes a hierarchical structure for memristor-based neuromorphic computing accelerators, with interfaces for customization. Other architectural-level simulators proposed in the literature and following a very similar approach include CIM-SIM^242^ and XB-SIM^243^.

Going deeper into details, the MNEMOSENE simulator^244^ adds cycle-accurate capabilities to tile-level simulations by actually executing in-memory instructions (in the context of Fig. 16, this could be interpreted as an Implementation Model, indicated by sphere G). It also allows the user to track all the control signals and the content of crossbar/registers, and due to the modular programming of the simulator, the user can easily investigate different memristor technologies, circuit designs, and more advanced crossbar modelling (e.g., considering read/write variability). Then, moving forward with the path toward the most accurate memristive neural network simulators, the PUMAsim proposed by Ankit et al.^154^ uses Verilog HDL to model the tiles and cores at the Register Transfer Level, which allows them to be mapped into a 45 nm Silicon-on-Insulator CMOS process for area estimation. Until this point, and regardless of their level of detail (System Level or Architecture Level) simulators could be framed between the cases described by nodes B-G from Fig. 16. The final step is to describe each of the constituting blocks in term of the required electrical devices, i.e., transistors and memristors.

### Circuit level simulation

To deal with neuro-inspired computing on the circuit-level, Dong et al.^245^ proposed NVSim which represents a simulator for emerging non-volatile memories like STT-RAM, PCRAM and ReRAM structures. This allows: i) estimation of access time, access energy and silicon area, ii) Design-Space exploration, and iii) optimization of the chip for one specific design metric. However, and similarly to NVMain^238^, NVSim focuses mostly on modelling non-volatile memory structures rather than in compute-in-memory units. Alternatives to overcome this limitation have been proposed, as for instance the simulator developed by Song et al.^246^ to evaluate their PipeLayer architecture, which considers highly parallel designs based on the notion of parallelism granularity and weight replication. This simulator is based on NVSim and provides a high-level functionality to cover the requirements for computer-in-memory simulations. This is also the case of the RAPIDNN^247^ which relies on H-SPICE and Nvsim simulations to evaluate the energy consumption and performance. Another alternative for circuit-level simulation has been largely covered in the literature when aiming to simulate simple crossbar structures of the 1R kind. This methodology initially reported by Chen^248^, and then further exploited in refs. ^249–251^, describes the electrical behaviour crossbar structure by its associated mathematical representation, as a system of coupled equations.

Although both previously described methods can tackle the challenge of circuit-level simulation of memristor devices (the second one in fact only for DC quasi-static signals) they fail to account for hybrid CMOS-memristor structures. For this scenario, it is crucial to consider simulators capable of dealing with industry-standard CMOS models, preferably at the SPICE level and if not, at least at the RTL level. This is the case of the work by Fei et al.^252^, although their proposed simulation tool was not evaluated for hybrid CMOS-memristor neural networks. In this regard, in our previous work^253^ we proposed a simulation routine which, from a set of given parameters (e.g., network size, memristor electrical characteristics and non-idealities, interconnections), creates a pre-trained hybrid CMOS-memristive neural network described as a SPICE netlist (i.e., a text file that describes the circuit). This procedure was successfully used to evaluate the accuracy, power dissipation, latency and other figures-of-merit of hardware-based neural networks during inference^250,253^. It also allows to study in detail the weight update process^254^ and the mitigation of stuck-at-faults^255^. To speed up the simulation process, we rely for this implementation in the FastSPICE simulator from the Synopsys Design Suite, although it is perfectly compatible with standard H-SPICE. A similar path was followed by Fritscher et al.^256^ but considering the Cadence Design Suite. A very interesting characteristic is that the environment combines the analogue circuit simulator Cadence Spectre with the Cadence Incisive, a system-level simulator, to model a complete system from the device to the system level in a very comprehensive manner. As a final remark, to fully cover Fig. 15, device-level-simulators like Ginestra^257^ or T-CAD^258^ are intended for physics-based simulations at the atomic level of a single device, and its output is then further used for fine-tuning the compact models used in SPICE simulations.

### Software-hardware co-design and hardware-aware neural architecture search

Software-hardware co-design tool chain implies the optimization of all components involved in the hardware implementation of neural networks, including the memristive device performance, circuit blocks, architecture hierarchy and communication between the blocks. There is a lack of an efficient commercial tool for software-hardware co-design, as device-level simulators do not consider architecture-level and communication on the chip, while architecture-level simulators lack the consideration of realistic device properties^159^.

In addition to hardware-level design considerations, the software-related design parameters selected for the neural network can also affect the hardware performance. These software-related design parameters include the number of neurons and layers in the network, the sizes of convolution kernels, activation functions, etc. For example, memristor-related non-idealities can be mitigated by optimizing the software-related design parameters for the neural network^259^. Reference ^260^ shows that neural network design parameters can be optimized to reduce the effects of conductance variations and conductance drift in memristors without compromising performance accuracy. Therefore, it is important to optimize both software and hardware parameters together to achieve high-performance accuracy and hardware efficiency of memristor-based neural network hardware and mitigate device non-idealities.

Such optimization lies within the domain of hardware-aware neural architecture search, which optimizes the design parameters of the neural network considering hardware feedback^261–264^, or in some cases, searches for the optimum hardware parameters^265,266^. For example, an optimum crossbar size^266^, ADC/DAC resolution, and device precision^265^ can be searched along with the software-related parameters of the neural network. References ^263,264^, take memristor device variations into consideration when searching for the optimum software-related neural network parameters. The design parameters search can be performed using reinforcement learning^264,266^, evolutionary algorithms^259,260,263,265^, or differential methods^261^. Hardware-aware neural architecture search is a promising approach to automate the software-hardware co-design of memristor-based neural networks.

## Example of memristive ANN analysis

To evaluate the feasibility of a memristive device (implemented in crossbar arrays) for image classification, we have developed a procedure for creating and simulating a single-layer perceptron (SLP)^57^. This neural network type is simpler than those considered in other more complex memristive ANNs, e.g. Multi-layer Perceptron (MLP)^54,196,267^, Convolutional Neural Networks (CNNs)^268^, Spiking Neural Networks (SNNs)^269^, among others (see Table 5). However, it allows studying and clarifying the limitations of ANNs caused by parasitic effects and non-idealities occurring in the synaptic layers implemented with crossbar arrays of memristive devices. Such effects include the impact of the non-negligible resistance of the line interconnections, the finite resistance window (*R*_LRS_/*R*_HRS_), the Signal-to-Noise ratio (SNR), the synaptic weight variability, and the inference latency, among others. The procedures here presented are valid regardless of the memory cell considered (1T1R or 1R). The presented procedure can be extended for MLPs relatively easily; in such case, the circuit generation phase is repeated as many times as layers have the MLP.

**Table 5:** Comparison of the accuracies obtained with different memristor-based neural network types and learning algorithms, both from simulation and experimental approaches

Neural Network type	Learning algorithm	Database	Size	Training	Accuracy		Platform	Ref.
					(Sim.)	(Exp.)		
Single-Layer Perceptron (SLP)	Backpropagation (Scaled Conjugate Gradient)	MNIST (*n* × *n* px.)	1 layer (n^2^ × 10)	Ex-situ	∼91%		SPICE sim. QMM model	^253^
	Manhattan update rule	Custom pattern	1 layer (10 × 3)	In-situ	ND		Exp.(TaO_X_/Al_2_O_3_)	^105^
		Yale-Face	1 layer (320 × 3)	In-situ	∼91.7%		Exp. (TaO_X_)	^194^
Multi-Layer Perceptron (MLP)	Backpropagation (Stochastic Gradient Descent)	MNIST (8 × 8 px)	2 layers (64 × 54 × 10)	In-situ	∼91.7%	∼91.7%	Exp. (HfO_2_)	^54^
	Backpropagation (Scaled Conjugate Gradient)	MNIST (*n* × *n* px.)	k layers (n^2^ × m×…× k × 10)	Ex-situ	∼96%		SPICE sim. QMM model	^253^
	Backpropagation	MNIST (14 × 14 px)	2 layers (196 × 20 × 10)	Ex-situ	∼92%	∼82.3%	Software/Exp. (HfO_2_)	^196^
		MNIST (22 × 24 px)	2 layers (528 × 250 ×…× 125 × 10)	In-situ	∼83%	∼81%	Software/Exp. (PCM)	^267^
		MNIST (28 × 28 px)	2 layers (784 × 100×…×10)	Ex-situ	∼97%		Software (Python)	^288^
	Sign- Backpropagation	MNIST (28×28 px)	2 layer (784 × 300×…×10)	In-situ	∼94.5%		Software (MATLAB)	^289^
Convolutional Neural Network (CNN)	Backpropagation	MNIST (28×28 px)	2 layer (1st Conv., 2nd FC)	In-situ	∼94%		Software	^268^
Spiking Neural Network SNN)	Spike Timing Dependent Plasticity (Unsupervised)	MNIST (28×28 px)	2 layer (784 × 300×…×10)	In-situ	∼93.5%		Software (C++ Xnet)	^269^

Note that in all cases the synaptic layers are implemented with CPAs and simulations are performed without having into account the line parasitics or realistic memristor models. Given that the CPA is a building block in these complex neural networks, realistic SPICE simulations of the CPA are still required.

For the sake of simplicity, ex situ supervised learning will be considered here. Once trained, the synaptic weights calculated by this software-based SLP are converted to conductance values which are then implemented with memristors (i.e., the conductance of each memristor is programmed to the values calculated by the software). The recognition of patterns from the MNIST^86^ dataset is considered for benchmarking. The workflow is summarized in the chart depicted in Supplementary Fig. 9. The overall process can be split into two parts: the first one comprises a set of MATLAB subroutines for creating, training, and writing the SPICE netlist for a SLP, while the second part relates to the SPICE simulation of the proposed circuit during the classification phase.

### Translation of the synaptic weights from the Software based ANN to conductance values

There are two possible ways to set each of the memristors placed in the crossbars to its corresponding conductance value from the ${{{{{{\bf{G}}}}}}}_{{{{{{\bf{M}}}}}}}^{{{{{{\boldsymbol{+}}}}}}}$ and ${{{{{{\bf{G}}}}}}}_{{{{{{\bf{M}}}}}}}^{{{{{{\boldsymbol{-}}}}}}}$ matrices. One is to simulate the programming phase, during which the required conductance in each device is achieved by the application of a train of pulses of controlled amplitude and width while monitoring the progressive increase in the device conductance until meeting a target. However, this process is very demanding in terms of simulation resources specially for large networks. Another possibility is to use a memristor compact model and estimate the value of the state variable in the Memory Equation that leads to the target conductance. For the case of the Quasi-static Memdiode Model (QMM) considered in refs. ^250,253^, this is done by adjusting the control parameter *λ* that runs between 0 (HRS) and 1 (LRS). The required value of *λ* is obtained by solving Eq. 14:

14 $$I={{{{\mathrm{sgn}}}}}\left(V\right)\left\{\frac{W\left(\alpha {{IR}}_{{{{{{\rm{S}}}}}}}{I}_{0}\left(\lambda \right){e}^{\alpha \left({{{{{\rm{abs}}}}}}\left(V\right)+{R}_{{{{{{\rm{S}}}}}}}{I}_{0}\left(\lambda \right)\right)}\right)}{\alpha {R}_{{{{{{\rm{S}}}}}}}}-{I}_{0}\left(\lambda \right)\right\}$$

for $I={g}_{{{{{{\rm{i}}}}}},{{{{{\rm{j}}}}}}}{\cdot V}$, with *g*_i,j_ being each of the elements of ${{{{{{\bf{G}}}}}}}_{{{{{{\bf{M}}}}}}}^{{{{{{\boldsymbol{+}}}}}}}$ and ${{{{{{\bf{G}}}}}}}_{{{{{{\bf{M}}}}}}}^{{{{{{\boldsymbol{-}}}}}}}$. In Eq. 14, *I*_0_(*λ*)=*I*_min_(1− *λ*)+*I*_max_*λ* is the diode current amplitude, *α* a fitting constant, and *R*_S_ a series resistance. Equation 14 is the solution of a diode with series resistance and *W()* is the Lambert function. *I*_min_ and *I*_max_ are the minimum and maximum values of the current amplitude, respectively. abs(*V*) is the absolute value of the applied bias and sgn() the sign function. As *I*_0_ increases in Eq. 14, the *I-V* curve changes its shape from exponential to linear through a continuum of states, as experimentally observed for this kind of devices^253^. This equation is solved for each of the memristors in the positive and negative array, as indicated in the Supplementary Algorithm 4. As a result, two different matrices (${{{{{{\boldsymbol{\lambda }}}}}}}_{{{{{{\bf{M}}}}}}}^{{{{{{\boldsymbol{+}}}}}}}$ and ${{{{{{\boldsymbol{\lambda }}}}}}}_{{{{{{\bf{M}}}}}}}^{{{{{{\boldsymbol{-}}}}}}}$) are produced. Note that for other memristive models the state variable would be calculated following a different equation (for instance in the Stanford model^270^).

The non-negligible resistance of the metallic lines connecting the upper and bottom electrodes of the memristors integrated in a crossbar structure produces an IR (voltage) drop along them that reduces the voltage delivered to the memristors. This phenomenon worsens for memristors located away from the input (crossbar’s row terminals) and output (crossbar’s column terminals) ports, as the interconnection lines required to reach such devices are increasingly longer. A widely accepted^104,271^, alternative design to minimize this problem consists in dividing the large crossbars into smaller ones (Supplementary Fig. 9b), whose reduced size improves their read margin (that is the portion of the applied voltage in the inputs that is actually delivered to the memristors). The number of partitions is denoted as NP, and the recommended size of each partition depends on the ratio of conductance between the memristors and the resistance of the metallic wires. Supplementary Fig 10 shows the simplified sketch of the partitioned crossbar and the interconnections required to realize the complete VMM. By exploding the integrability of the crossbar with CMOS circuitry, vertical interconnects used to connect the outputs of the vertical crossbar partitions may be placed under the partitioned structure (as well as the analogue sensing electronics) allowing the partitioned crossbar to maintain a similar area consumption than the original non-partitioned case^104^. The vertical interconnects are grounded through the sensing circuit (i.e. the TIA) to absorb the currents within the same vertical wire. To achieve this partitioned structure, both the ${{{{{{\boldsymbol{\lambda }}}}}}}_{{{{{{\bf{M}}}}}}}^{{{{{{\boldsymbol{+}}}}}}}$ and ${{{{{{\boldsymbol{\lambda }}}}}}}_{{{{{{\bf{M}}}}}}}^{{{{{{\boldsymbol{-}}}}}}}$ matrices are subdivided into smaller portions (as shown in the upper part of Supplementary Fig. 9b). Each of these partitions is mapped to a different memristor crossbar. For instance, those 4 different matrices are mapped to the 4 different crossbars in Supplementary Fig. 10.

### Creation of the memristive ANN circuit representation

**Fig. 17:**
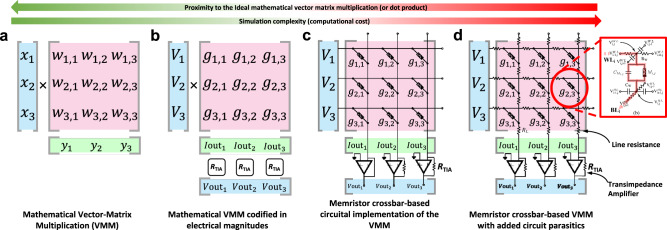
Different representations of the Vector Matrix Multiplication operation typical from a synaptic layer. (**a**) Unitless mathematic VMM operation. (**b**) Mathematic VMM operation involving electrical magnitudes. (**c**) Electrical circuit representation of the memristive crossbar-based analogue VMM operation. (**d**) Realistic memristor crossbar representation considering the line resistance (*R*_L_) and the interline capacitances (see the inset showing a circuit schematic of a memristive cell in a CPA structure considering the associated wire parasitic resistance and capacitance). Aspects such as device variability are captured by the memristor model employed.

**Fig. 18:**
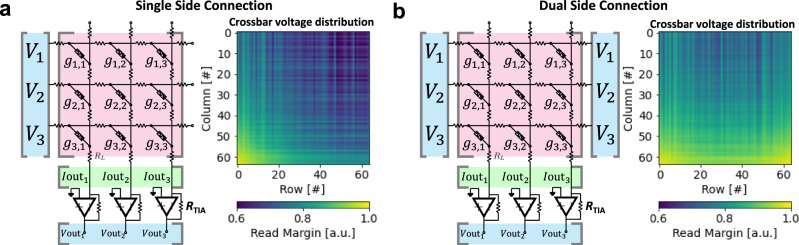
Connections schemes used to feed the CPA with the input pattern. **a** Single Side Connect (SSC) and (**b**) Dual Side Connect (DSC). On the SSC case, the input stimuli are applied only to the inputs of one side of the CPA, while the other is connected to high impedances (or remain disconnected). **b** In the DSC case, both terminals of a given wordline (horizontal lines in the CPA) are connected to the same input voltage, which thereby reduces the voltage drop along the wordlines.

In the next step, the software (MATLAB in this example) is used to write (line by line) the SPICE netlist that corresponds to the *n*^2^×10 memristor crossbar-based ANN, taking into account the connection scheme (positive and negative matrixes, each of them partitioned) and the control logic necessary to perform the inference phase. Figure 17 describes the different abstraction levels going from the pure mathematical representation of the VMM (Fig. 17a), then to the block diagram involving the electrical magnitudes (voltages, conductances, resistances and currents, see Fig. 17b), then to a circuit schematic with no parasitics (including in this stage the memristors and the necessary analogue electronics, see Fig. 17c), to finally reach the equivalent analogue circuit that performs the VMM including the circuit parasitics (Fig. 17d). In this example, we use the fprintf() function of MATLAB^94^, and we employed a memristor cell that takes into account all the wire resistances and capacitances. The custom-made MATLAB code receives as input arguments the array size and partitioning scheme, and it automatically determines the number of memristors to place and how to connect them to the adjacent line resistances to realize the crossbar electrical structure. Such a source code uses nested for loops that iterate over the number of rows and columns, creating the crossbar structure. Also, the parasitic capacitance between parallel adjacent lines in the same plane (i.e., between adjacent rows and columns), between the top-bottom line intersections, and between the bottom lines and ground are added. By this, we can account for the delay propagation through the crossbar, also known as latency (that is, when the goal is measuring the time elapsed since a pattern is applied in the SLP inputs until the output stabilizes). As a result, each memristor in the crossbar structure is connected to 4 resistors and 5 capacitors, as shown in Fig. 17d. As an example, the resulting SPICE code for a SLP to classify 4×4 pixels images is shown in Supplementary Algorithm 5. In order to avoid voltage loses at the wires of the crossbar, we employed a Dual Side Connection scheme. Despite the increased peripheral circuitry complexity, this scheme improves the voltage delivery to each synapse^248^ by connecting the two terminals of each wordline to the same input stimuli. The difference between Dual Side Connection and Single Side Connection is shown in Fig. 18. In practice, when designing the circuits for input voltage supply for the Dual Side Connection scheme on a chip, any mismatches and variations in voltages *V*_i_ (Fig. 18b) should be avoided. The voltages *V*_i_ from both sides of the crossbar should be identical with carefully designed communication wires. Any variations caused by the difference in the length of the wires connecting the crossbar rows to the input supply voltages can lead to undesirable voltage drops and issues related to sneak path currents.

The input stimuli are obtained by scaling each of the 10,000 unrolled grayscale images from the MNIST test dataset, previously stored in a *n*^*2*^*×*10,000 vector, by a voltage *V*_READ_ as shown in Fig. 4c. *V*_READ_ is chosen such as to prevent altering the memristor states during the inference simulation. In this way, during the inference process each of the test images is presented to the crossbar as a vector **V** of analogue voltages *V*_i_ in the range [0, *V*_READ_].

**Fig. 19:**
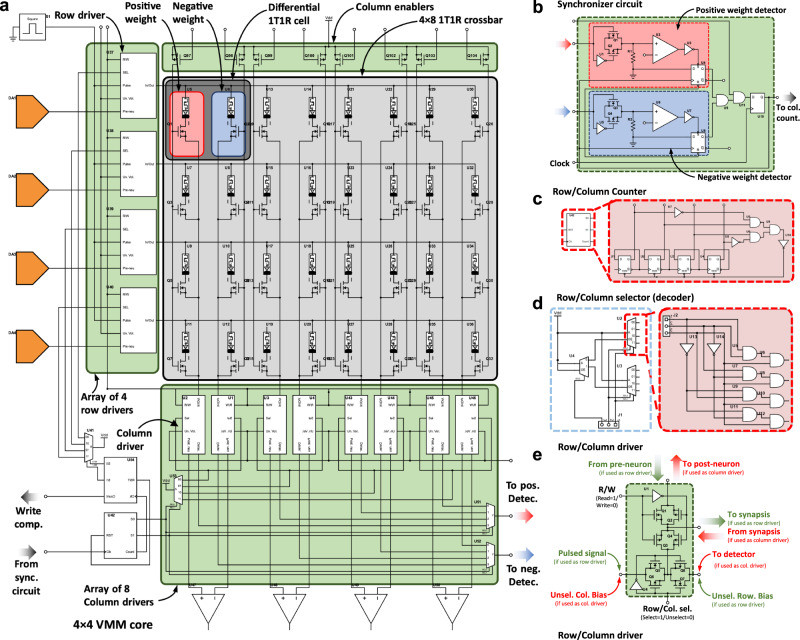
Detail of the control circuits used for the dual inference/write procedures. **a** complete circuit schematic for a 4×8 1T1R crossbar array. **b** Detail of the synchronizers including the sense amplifiers used to detect the correct programming of a given memristor. **c** Address block, essentially a counter which sequentially addresses each memristor in the crossbar. **d** Row and column decoders, used to enable the memristor addressed by the address block. **e** Row and column driver, used to bias the rows with the voltage input or with the programming signal, and to connect the columns to the output neurons (during inference) or the sense amplifier (during write-verify).

During the inference phase, the inputs of the partitioned crossbar need to be connected to the voltages representing the brightness of the pixels, and the outputs of the crossbar need to be connected to peripheral analogue circuits consisting on adders constructed using few resistors and TIA (see Supplementary Fig. 9c left and Fig. 19a)^250,253^. During the write phase, the partitioned crossbar needs to be connected to the peripheral circuitry necessary to produce the electrical stimuli that program the memristor conductance to the values calculated via MATLAB. This peripheral circuitry consists of a crossbar address block, Row/Column address decoders, Row/Column selectors, and a Write Acknowledge block (see Supplementary Fig. 9c right and Fig. 19a). The crossbar Address Block (crossbar-AB) is a circuit that produces a pulse every time the memristor located in the {i,j} position is completely written in all partitions (thereby working as a counter, as depicted in Fig. 19b), which thereby results in *n*^2^*/*NP·*10* output pulses (corresponding to the number of memristors in each of the NP partitions). These pulses (generated by a sensing amplifier comprising a comparator and a latch circuit as shown in Fig. 19c) are propagated to the crossbar Column Decoder (crossbar-CD). The crossbar-CD is an asynchronous counter with 4 parallel outputs (see Fig. 19d) used to indicate, in a binary code, which column to address during the programming Write-Verify loop. Also, the column decoder outputs a pulse every time 10 pulses are received, which can also be seen as a pulse every time a row is completely programmed. This pulse is sent to the crossbar Row Decoder (crossbar-RD), which is a similar counter but with *n*^2^*/*NP parallel outputs and thereby *S* control inputs, with *S* being the nearest integer higher than log_2_(*n*^2^/NP)). The codes of the addressed row and column are then propagated to the crossbar Row/Column Selector (crossbar-RS/crossbar-CS). Both the crossbar-RS and crossbar-CS blocks comprise two stages. The first one, shown in Fig. 19d, is a digital de-multiplexer with *S* control inputs (for a crossbar with 10 columns, the control input is a 4 bit code, S_1_-S_4_, and it can be generalized as the nearest integer higher than *log*_*2*_*(x)*, with *x* the number of rows/columns). For a given control input, only one of the parallel outputs is active at a time. Thereby this produces a sparse column vector of size 10 (crossbar-CD) or *n*^2^*/*NP (crossbar-RD). The second stage is a column array of 10 (crossbar-CD) or *n*^2^*/NP* (crossbar-RD) of analogue switches that connect the input node of each crossbar row to *V*_WRITE_ or *V*_READ_ (for addressing that particular Row during the write procedure), *V*_DD_/2 (if another row is being addressed) or to *V*_i_ (when the ANN is operating in the inference state). The column selector is a similar array that connects the columns output nodes to a sensing amplifier (sensing amplifier, a TIA coupled to a voltage comparator) if that particular column is being addressed, or *V*_DD_/2 (if another column is being addressed). Each of these analogue switches comprises 4 pass gates cells, as indicated in Fig. 19e. Figure 19a shows a bigger picture of this latter concept, indicating how the multiplexor in the Row/Column selector blocks is connected to the array of analogue switches, which are ultimately connected to the crossbar block. After the MATLAB code generates a netlist, it is passed to a SPICE simulator which evaluates the voltage and current distributions in the crossbar circuit and then passes the resulting waveform back to the MATLAB routine for metrics extraction (Supplementary Fig. 9d). In this example, all the peripheral circuitry connected to the crossbar have been designed using a commercially available 130 nm CMOS process, whose model is available in SPICE libraries.

### SPICE Simulation and metrics extraction

#### Inference Procedure

**Fig. 20:**
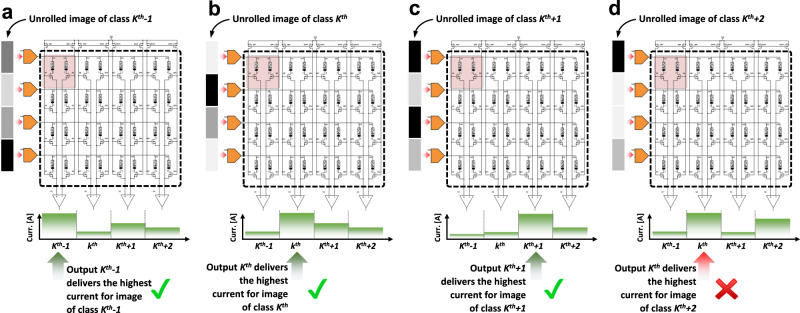
Schematic representation of the *n*^2^×1 column vectors of analogue voltages being fed to the SLP. 4 cases are represented: **a**–**c** corresponds to the correct classification of images from classes *k*, *k*+1 and k-1, respectively (for instance, in the case of the MNIST database, they might be images of the ‘5’, ‘6’ and ‘4’ digits). **d** Depicts the case of misclassification, as the highest current corresponds to the *k*+1 output for an image from class *k*.

Between the inference and write routines, the inference is simpler. During this phase each of the test images from the dataset are presented sequentially to the inputs of the SLP as a column vector **V** of size *n*^2^×1, where each of its elements are voltages *V*_i_ within the range [0, *V*_READ_] (see Supplementary Fig. 9). Each of these image vectors produces a current through the wordlines and bitlines, as they flow through the memristors (artificial synapses). Depending on the strength of such synapses, the current will be high (strong synapsis – high memristor conductance) or low (weak synapsis – low memristor conductance). The total current flowing out of each bitline of the crossbar is sensed at the bitline output. For a dataset with *m* classes (i.e. *m* possible output values), and considering a differential encoding (i.e., each synaptic weight is represented with 2 memristors) a crossbar with 2·*m* bitlines is required, which results in *m* output current signals. The main idea behind the inference phase is that for an input image of class *k*, the current flowing out of bitline *k* will be the highest. Similarly, for classes *k*-1 and *k*+1, the bitlines with the maximal current will be *k*-1 and *k*+1. A schematic representation of this behaviour is presented in Fig. 20. As seen, the case of misclassified images exists, corresponding to the highest current for an image from class *k* not being provided by column *k*. The selection of the highest current at a given time *t* is performed ex situ (i.e. via MATLAB) by processing the recorded current traces. This could be easily implemented on-chip by including a softargmax()^148^ CMOS circuit as those discussed in Section SoftArgMax function (block 8). This block has to be tailored for the dynamic range of the output current, as it depends on the size of the crossbar and the resistance of the lines.

To study the inference phase, different metrics were defined and they are divided in two groups, which can be referred as: (i) pattern recognition metrics (which are intrinsic characteristics of the SLP or ANN and were introduced in Table 2 and Fig. 14) and (ii) electrical measures (related to the particular memristor-based implementation of the SLP). The second group comprises the average output current range, the power consumption of the crossbar (useful not only to address the energy requirements of the crossbar, but also to determine where the power dissipation takes place: in the interconnections or in the memristors), the Signal-to-Noise ratio of the output current signals, the inference latency, the read and write margins (that is, the portion of the voltage applied in the crossbar inputs that effectively reaches the memristors during the read or write operations) and the maximal operational frequency of the complete neuromorphic circuit (crossbar plus CMOS electronics).

#### Write-verify procedure

**Fig. 21:**
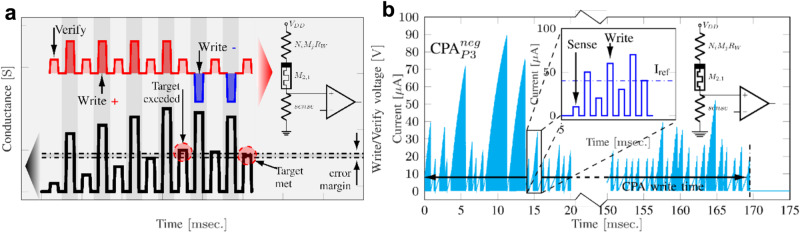
Write-verify approach for conductance programming. **a** Schematic representation of the Write-Verify loop approach for programming the memristors in the CPA to a given conductance value. Reproduced with permission under CCBY 4.0 license from ref. ^253^. **b** Sensed output current for a SLP partition (one small CPA) during the programming phase in a Write-Verify loop procedure. The greater the peak, the higher the conductance level being programmed. The inset in the center shows a schematic representation of the current measured during the verify and write pulses, as well as the current target. The inset in the right of both panels shows a schematic of the equivalent circuit using during the verify phase. Reproduced with permission under CCBY 4.0 license from ref. ^254^.

During the write operation each memristor in a crossbar (*M*_i,j_) is individually addressed and supplied with a train of alternating read and write pulses of amplitude *V*_READ_ and *V*_WRITE_ respectively, that causes a gradual increment (or decrement) of the memristor conductance. Such addressing procedure is performed following the *V*_DD_*/2* approach as it minimizes the line disturbance^248^. Within this writing method, the non-addressed rows are set to a constant source of value *V*_DD_/2. Similarly, the output node of the column of the addressed memristor is grounded through the sensing amplifier, which measures the current flowing out of this column (the other columns are at *V*_DD_/2). Such current is proportional to the applied voltage pulses and the memristor conductance plus the parasitic wire resistance corresponding to the addressed device (*M*_i,j_). This allows to estimate the conductance of the addressed memristor. This process is represented by the simplified equivalent circuit shown in the inset of Fig. 21.

Before starting the write process, we translate the conductance matrix for each partition to a currents matrix, by multiplying each element *g*_i,j_ by *V*_READ_. In this way, we obtain a measurable quantity for each of the elements in the conductance matrix. The goal of the write cycle is to gradually increase the conductance of a given element in the crossbar until sensing that the current flowing through it has reached the value indicated by the currents matrix for the same position (target value), which means that the desired conductance was also reached. The writing procedure for the addressed memristor *M*_i,j_ begins by sensing the output current during the read pulse of voltage *V*_READ_. In case this current is lower than a target value (*g*_i,j_·*V*_READ_*)*, a write pulse of voltage *V*_WRITE_ is applied (*V*_WRITE_*>V*_READ_), causing an increment in the *M*_i,j_ conductance. Then a new read pulse is applied, and the current is sensed again. This process continues iteratively until the sensed current during the read pulse meets the target value. Once reached, the sensing amplifier outputs a pulse that indicates the completion of the writing procedure for the addressed memristor (*M*_i,j_), stopping the train of read/write pulses and preparing the following devices to be programmed.

It is worth noting that the partitioned architecture allows the simultaneous programming of the *M*_i,j_ memristor of all partitions using a smaller control circuit. Let us assume that the devices to be programmed are the *M*_i,j_ memristors of a 2*×(n*^2^*×*10*)* crossbar with NP partitions, such as the one presented in Supplementary Fig. 9d. In this case, the *i*^*th*^ output of the row decoder (*n*^*2*^*/*NP outputs) will be the only active output, as well as the *j*^*th*^ output (10 outputs) of the column decoder. Then these output vectors are passed to every Row/Column selector, which simultaneously select the *M*_*i,j*_ memristor in every crossbar. This causes all the *i*^*th*^ rows to be connected to a train of alternating read and write pulses and all the *j*^*th*^ columns to be connected to the partition sensing amplifier (each crossbar partition has its own sensing amplifier). All other rows and columns are connected to *V*_DD_/2. The current flowing through each of the *M*_i,j_ memristors (and therefore out of the *j*^*th*^ columns) is sensed by its associated sensing amplifier until the target conductance value for that *M*_i,j_ memristor is achieved. Then the associated sensing amplifier propagates an acknowledge pulse (ACK) to the Write Acknowledge block, which then disconnects the addressed memristor from the write pulse generator to prevent it from being further potentiated/depressed. This block waits for the ACK pulses from the sensing amplifiers of every partition. Once all ACK pulses are received, the *i*^*th*^*,j*^*t*h^ position of all crossbars is considered to be successfully written, and by the time the Write Acknowledge block receives the following system clock pulse, it instructs the crossbar address block to address the M_i,j+1_ memristor and the write sequence starts again. This process continues until the crossbar address block has addressed all the memristor positions in the crossbar partitions (*n*^2^*/*NP*×*10 positions).

Memristive ANNs help on reducing the data transfer typical from digital processors, by performing computations locally within the memory. However, these systems have their own unique challenges which still limit their further development. To exploit the intrinsic advantages of crossbar-based computation, a careful design of the system architecture is crucial, as otherwise, the peripheral CMOS circuits become a bottleneck impeding the power, area, and latency improvement that in-memory-computing could achieve. A main goal in designing these architectures is to keep this peripheral overhead to a minimum without sacrificing performance. However, and despite that the concept of analogue neuromorphic accelerators has been investigated for over the last decade, papers reporting true full-on-chip hybrid CMOS/memristors have only started to appear in the last two years. Thereby, performance metrics obtained from systems heavily relying on extensive off-chip electronics should be analysed carefully.

While the crossbar computations are performed in the analogue domain, digital encoding is used for the external routing/processing. Although every block in the peripheral circuit supposes a considerable effort by itself, the conversion between the analogue and digital domains, constitutes the main challenge in the design of memristive ANN. This is achieved by the analogue-to-digital and digital-to-analogue converters, and a primary trade-off that needs to be made in the design of a memristive ANN is that between energy efficiency and precision: high precision comes at the cost of greater ADC/DAC silicon and thereby power consumption. Nonetheless, there are various ways to reduce this overhead, such as by encoding the weights to reduce ADC precision, by multiplexing techniques of the crossbar outputs or reducing the number of available states in the memristors. Given the overhead that ADCs imply, another option points towards a fully analogue approach, pushing the analogue/digital frontier towards the end of the neural network: some architectures remain mostly digital by using binary inputs and quantized/binary weights for the VMM; some consider analogue inputs and weights, but the VMM product is immediately digitalized and processed in the digital domain; and others are almost fully analogue, with digitalization only taking place after the activation functions and softargmax() blocks.

Beyond the CMOS circuits required for pre/post processing the signals, the performance of memristive ANNs is also threatened by the non-idealities intrinsic to the crossbar geometry and the individual memory devices of the crossbar. Non-ideal physical properties of the devices compromise the reliability, scalability, accuracy, latency and power consumption of the memristive ANN. The available number of conductance states, and the potentiation and depression linearity play a fundamental role in the weight update procedure and sets basic requirements for the peripheral CMOS circuitry in charge of performing that process. Consequently, device–hardware co-design (i.e. optimizing the device characteristics based on the circuitry capabilities, and vice versa) is indispensable, and a powerful tool to enable this process is the realistic electrical simulation of hybrid CMOS/memristor systems.

The simulation of ANNs allows to tackle design problems before fabrication as well as to estimate the hypothetical performance achievable by a given memristor technology. Depending on the requirements, it can go from a high abstraction level, with little (if any) connection to the actual devices, down to the circuit level, using standard SPICE/Verilog compact behavioural models for the CMOS devices and the memristors. In between these two extremes, there are various transaction-level approaches which consider a varying level of detail to represent both the ANN architecture as well as the communication between them. The selection of the most suitable simulation technique depends thereby in the requirements of the specific design stage: the closer it gets to the tape-out, higher accuracy in the simulation is required (achievable with circuit level simulations), instead, for the early design stages, system-level modelling is enough to get a quick estimation of the achievable performance in large, complex ANNs. In any case, properly combining this many different simulation tools will ultimately lead to the optimization and further development of the memristive ANNs.

### Supplementary information

Supplementary Information

## Data Availability

The code examples provided in the Supplementary Information are publicly available at https://github.com/aguirref/supplementary_ANN_algorithms.

## Code availability

The MNIST dataset used for the image classification in this study is openly available at https://yann.lecun.com/exdb/mnist.
