# Supplementary material for: Hardware implementation of memristor-based artificial neural networks
Source: Nat Commun. 2024 Mar 4;15:1974. doi: 10.1038/s41467-024-45670-9 (PMC10912231; doi:10.1038/s41467-024-45670-9)
Supplement: Supplementary file 1 — Supplementary Information [file 41467_2024_45670_MOESM1_ESM.pdf]

Hardware implementation of memristor-based artificial neural networks

Fernando Aguirre[1,2], Abu Sebastian[3], Manuel le Gallo[3], Wenhao Song[4], Tong Wang[4], J. Joshua Yang[4], Wei Lu[5], Meng-Fan Chang[6], Daniele Ielmini[7], Yuchao Yang[8], Adnan Mehonic[9], Anthony Kenyon[9], Marco A. Villena[1], Juan B. Roldan[10], Yuting Wu[5], Hung-Hsi Hsu[6], Nagarajan Raghavan[11], Jordi Suñé[2], Enrique Miranda[2], Ahmed Eltawil[12], Gianluca Setti[12], Kamilya Smagulova[12], Khaled N. Salama[12], Olga Krestinskaya[12], Xiaobing Yan[13], Kah-Wee Ang[14], Samarth Jain[14], Sifan Li[14], Osamah Alharbi[1], Sebastian Pazos[1], Mario Lanza[1*]

[1] Physical Science and Engineering Division, King Abdullah University of Science and Technology (KAUST), Thuwal 23955-6900, Saudi Arabia

[2] Departament d'Enginyeria Electrònica, Universitat Autònoma de Barcelona (UAB), 08193 Barcelona, Spain

[3] IBM Research – Zurich, Rüschlikon, Switzerland

[4] Department of Electrical and Computer Engineering, University of Southern California (USC) Los Angeles, CA 90089, United States of America

[5] Department of Electrical Engineering and Computer Science, University of Michigan, Ann Arbor, Michigan 48109, USA

[6] Department of Electrical Engineering, National Tsing Hua University, Hsinchu 30013, Taiwan

[7] Dipartimento di Elettronica, Informazione e Bioingegneria, Politecnico di Milano and IUNET, Piazza L. da Vinci 32, 20133 Milano, Italy

[8] School of Electronic and Computer Engineering, Peking University, Shenzhen, People's Republic of China

[9] Department of Electronic and Electrical Engineering, University College London (UCL), Torrington Place, WC1E 7JE London, United Kingdom

[10] Departamento de Electrónica y Tecnología de Computadores, Facultad de Ciencias, Universidad de Granada, Avenida Fuentenueva s/n, 18071 Granada, Spain

[11] Engineering Product Development (EPD) Pillar, Singapore University of Technology & Design, 8 Somapah Road, 487372, Singapore

[12] Computer, Electrical and Mathematical Sciences and Engineering Division, King Abdullah University of Science and Technology (KAUST), Thuwal 23955-6900, Saudi Arabia

[13] Key Laboratory of Brain-Like Neuromorphic Devices and Systems of Hebei Province, Hebei University, Baoding 071002, PR China.

[14] College of Design and Engineering, National University of Singapore (NUS), Singapore

*Corresponding author Email: mario.lanza@kaust.edu.sa

Supplementary Algorithm 1. Iterative algorithm for rescaling the image database. Note that a for loop is used to iterate over each image in the dataset. In this case, the rescaling of 28×28 px. to 8×8 px. is considered. Once selected, the image is displayed in a $p \times p$ (28×28 in this case) representation (reshape() function), which is then resized (to 8×8 px.) with the imresize() function to allow rendering with fewer pixels. Finally, the image is again reshaped to a column vector format ($n^2 \times 1$, in this case 64×1 px.) and stored in a matrix, by horizontally concatenating each column vector.

```
%% this part of the code performs the dataset re-scaling
% this block re-scales the train dataset
for i = 1:60000  % This for loop iterates over the train image dataset
    digit = reshape(images(:,i),[28,28]);  % This reshapes image i from p^2x1 to pxp
    digit_resized = imresize(digit,[8,8]); % This resizes the image from pxp to nxn
    if limit_output==1                     % This block limits any value
        digit_resized(digit_resized>1)=1;  % outside the range [0,1] to that
        digit_resized(digit_resized<0)=0;  % range
    end
    images_8x8(:,i) = reshape(digit_resized, [8*8,1]); % The re-scaled images are re-
                                                       % shaped back to the n^2x1 format
                                                       % and stored in the images_mxm
                                                       % variable
end
labels_8x8 = labels;                       % This stores the labels in a new variable

% and this part re-scales de test dataset.
for i = 1:10000
         digit = reshape(images_t10k(:, i), [28,28]);
         digit_resized = imresize(digit,[8,8]);
         if limit_output==1
                  digit_resized(digit_resized>1)=1;
                  digit_resized(digit_resized<0)=0;
         end
         images_t10k_8x8(:,i) = reshape(digit_resized, [8*8,1]);
end
labels_t10k_8x8 = labels_t10k;
```

Supplementary note 1: Advanced mapping methods, bit-slicing, and tiling

In most cases of mapping neural networks to memristor-based hardware, it is not enough to convert the weights to conductance values. Circuit and architecture designers often have to deal with a limited number of conductance states in memristive devices and non-linear conductance distribution, optimization of the crossbar sizes, mapping large weights matrices into smaller crossbars, and mapping different types of neural network layers with different distributions of weights. All these challenges have corresponding solutions related to crossbar mapping techniques.

One of the most common techniques to map high-precision ANN weights into low-precision memristive devices is called bit-slicing or weight-slicing [1], [2] Bit-slicing implies that n-bit neural network weights are represented by 2^{n-m} m-bit memristive devices (devices with 2^m conductance levels). This can be done due to the following reasons: (1) when the number of stable conductance levels in memristive devices is limited and lower than the required weight precision, (2) when low-precision ADC is used and cannot support high-resolution crossbar weights, or (3) when the device-to-device or cycle-to-cycle conductance variation of memristors is too high and can affect the accuracy of VMM operation. A lower number of bits per device can decrease the effect of conductance variation on the VMM output. However, it also reduces the storage density of the crossbar, and, in turn, increases on-chip area due to the increased number of devices that should be used per single synapse [1].

Concerning the mapping of positive and negative weights, bit slicing has proven to be very useful when combined with 2's complement codification of the binarized synaptic weights [3]–[8]. In this approach, it is possible to avoid the differential codification using two high-precision (i.e. with many well defined levels) memristors, and to replace them by a single array of low-precision memristors per synaptic weight. A different approach for mapping has been suggested in [9], where the training process involves constraining twice the number of weights to be nonnegative and associating them with individual devices. This method results in a more natural connection between neural network parameters and conductances, and also permits weight regularization to be utilized as a way of reducing power consumption.

The other challenge in memristor-based neural network hardware designs is to create generalized designs fitting various sizes of weight matrices and preserving efficient hardware utilization. This often implies a fixed size of the crossbars throughout the design. In turn, large weight matrices cannot be mapped into a single crossbar array. Therefore, a technique called tiling is used to divide a weight matrix and map it to several crossbar tiles or sub-crossbars [1], [10]. There are several reasons to apply tiling: (1) when mapping large weight matrices to the hardware, which cannot fit into a single crossbar array, (2) when reducing required ADC resolution for a large crossbar, (3) when optimizing the size of the crossbar to avoid IR drop due to interconnects and long crossbar wires.

Same to the bit-streaming technique, both bit-slicing and tiling produce additional hardware overhead required to calculate partial sums [1]. In the case of tiling, partial sums are produced by each tile or sub-crossbar, and then accumulated to form a final VMM value using adder circuits. In the case of bit-slicing, several memristive devices representing a single weight are arranged to neighbouring columns of a single crossbar or multiple crossbar tiles. When all devices representing a single weight are in the same crossbar, multiplexers and sample-and-hold circuits are used to process and store the partial sums before calculating the final VMM value. When the components of a single weight are stored in different crossbar tiles, the partial sums from these tiles can be read in parallel but still require an adder circuit.

Non-idealities of memristive devices can also affect weight mapping. For example, the non-linear conductance distribution of the device should be considered, when quantizing and converting neural network weights to the conductance levels. If the example shown above assumes that it is possible to achieve linear and uniform conductance distribution, most of the memristive devices experience non-linearly distributed conductance states. In this case,

quantized weights should be mapped to non-uniformly distributed discrete states before mapping to the conductance values. Moreover, advanced mapping methods can be used for alleviating the effect of memristor non-idealities [1]. For example, ANN weights more sensitive to variations and defects can be mapped to defect-free or low-variation memristive cells in a crossbar [11], or closer to the voltage source to be affected less by IR drop occurring in the crossbar columns further from the voltage source due to wire resistances [1].

In addition, for more complex neural networks than ANN, the distribution of the weights in different layers may vary. For example, weight distribution in convolution layers in CNNs is different from fully-connected layers. Therefore, such layers require additional design considerations, including unrolling convolution kernels, and rearranging or duplicating the weights to achieve optimized hardware design [12], [13].

Supplementary Note 2: Memristor variability and thermal heating problem

Nanoscale size and non-volatility make memristor devices advantageous in implementation of fast, area and energy-efficient neural network accelerators. Typically, memristor are arranged into crossbar arrays and are stacked into 2.5D and 3D heterogeneous architectures [14]. However, downscaling of the feature size F of memristor and their utilization in dense architectures also make them susceptible to thermal heating problem. In particular, miniaturization of the NiO device from 100 nm to 30 nm led to increase of temperature from 400K to 1800K [15]. Moreover, increase of temperature leads to decrease of the *expected shortest lifetime* (ESL) [14] of a memristor and shrinking it's a R_{on}/R_{off} ratio leading to low reliability and data loss [16]. In addition, a hot "aggressor" cell negatively affects the performance of surrounding "victim" cells resulting in thermal cross-talk problem. Their sensitivity level depends on the resolution of the device, crossbar array pitch length, materials used in conducting filament (CF) and electrodes. Similarly, heterogeneous parts of the hardware also have different thermal densities and cause inter-die thermal coupling and hotspots as shown in Supplementary Figure 1 for ISAAC [17], [18]. As a result, in 2.5D design lifetime of a memristor is close to ESL, whereas in 3D design it reduces below 2.6 years [14]. In other words, increase of temperature zoom in malfunctions associated with device-to-device and cycle-to-cycle variability.

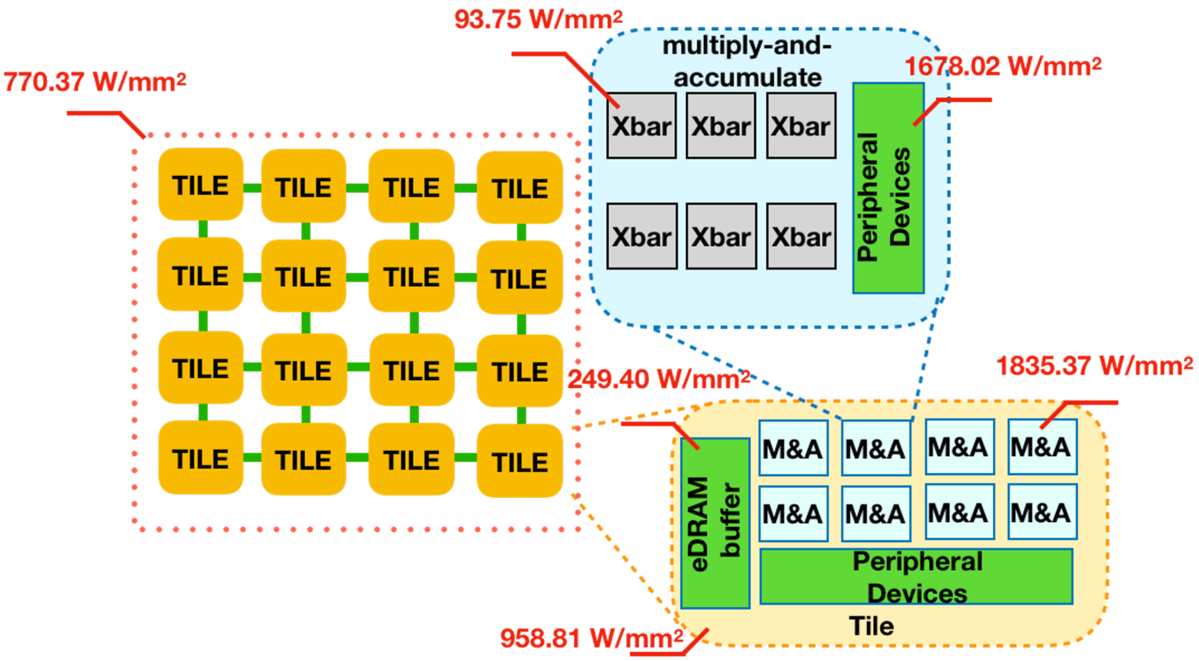

Supplementary Figure 1. Power density in ISAAC.

It is important to note, that due to its cumulative nature, thermal effects have more impact on memristor-based ANN accelerators rather than memristor-based storage devices. In addition, different workloads result in different thermal distribution. For instance, the temperature difference between VGG16, InsecptionV3 and ResNet50 neural networks mapped to the same crossbar arrays can reach up to 17 ºK, as illustrated in Supplementary Figures 2a, 2b and 2c, respectively [19]. Another work demonstrated that thermal problems can be mitigated via memristors static allocation considering the thermal distribution. Comparison of "naïve", "strike" and "chess" allocation schemes on memristor crossbar arrays for the same workload allowed to reduce the temperature difference between neighbouring cells down to 8 ºK as seen in Supplementary Figures 3a, 3b and 3c, respectively [20]. This became the basis of numerous studies addressing the thermal challenges in memristor-based accelerators. The

proposed solutions presented different re-mapping and optimization schemes at weight [19], [21] row/column [22], [23], subarray and arrays levels [19], [24].

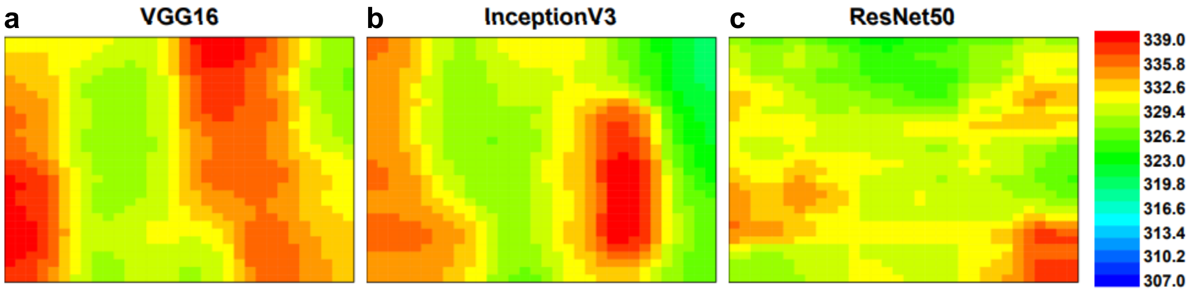

Supplementary Figure 2. Thermal distribution of memristor crossbar array running three different workloads (a) VGG16; (b) InceptionV3, and (c) ResNet50.

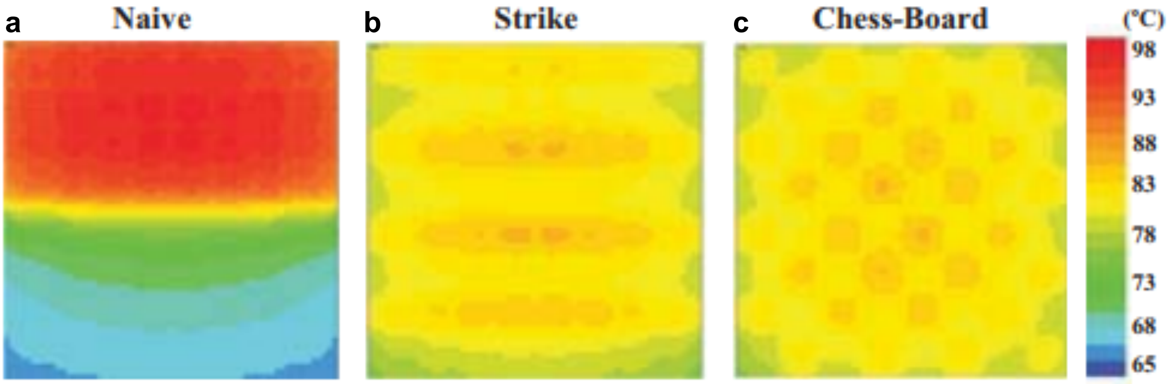

Supplementary Figure 3. Thermal distribution of memristor crossbar array running the same workload using three different static allocation schemes: (a) naïve, (b) strike, and (c) chess.

All of these re-mapping solutions rely on having a thermal heat map of crossbar array, the neural network model and the hardware characteristics. To construct such a heat map, synaptic weights of a neural network are first mapped to the memristor crossbar arrays using the "naïve" approach. Then, the temperature of each memristor is sensed by individually assigned sensors (Ideal Thermal Sensors -TPS-Ideal- approach) and ranges between 273 ºK and 400 ºK. According to the empirical results, the critical temperature above which memristor performance starts to degrade is ~340 ºK. Therefore, a threshold temperature typically is set to 330 ºK. To reduce redundancy of temperature sensors, a limited TPS approach can be adopted. Here, temperature is collected at the hottest points of the memristor crossbar and temperature of other rows is estimated using the equation below:

$$\text{Estimated } T_j = T_{k\times l} + \delta + \alpha \qquad (SI-1)$$

where $\delta = \frac{j}{k}$, $\quad 0 \le l < \left|\frac{n}{k}\right|$; $\alpha = T_{k\times(l+1)} - T_{k\times l}$; $T_{k\times l}$ and $T_{k\times(l+1)}$ are consecutively obtained temperatures of the two rows; $k \times l \le j \le k \times (l+1)$. In Hybrid Memory Cube (HMC) design, temperature sensors are placed at the centre and corners of the memristor crossbars [23]. Knowing the heat distribution in the memristor crossbars, the following re-mapping approaches can be applied to mitigate its effects.

A) Weight Pruning (WP).

In temperature-aware weight adjustment (TAWA) scheme [22], when the temperature measured or estimated for a memristor cell is above 330K (temperature threshold), the cell is considered as "hot" and the weight mapped to it is pruned.

B) Temperature-aware row adjustment in a crossbar arrays

Here, effective rows of a neural network are mapped to cold rows of a crossbar array, whereas ineffective rows are mapped to hot rows as in Supplementary Figure 4. Neural network matrix rows are classified to *effective* and *ineffective* rows using Summed Weight Variations (SWV) metric and predefined threshold θ. Memristor crossbar array rows are categorized as cold if temperature is below threshold 330 ºK, otherwise rows are hot. The Summed Weight Variations (SWV) metric can be estimated based on equation below:

$$SWV_{pq} = \sum_{j=0}^{m} \left| w_{pj} - g_{qj} \right| \qquad (SI-2)$$

where q is the row of $n \times m$ memristor crossbar array; w_{pj} is the weight at location (p,j) and g_{qj} is the actual weight at location (q,j). SWV value changes with temperature. If $SWV_{pq} > \theta$, rows are considered as effective, otherwise as ineffective. Different applications require different levels of θ [23].

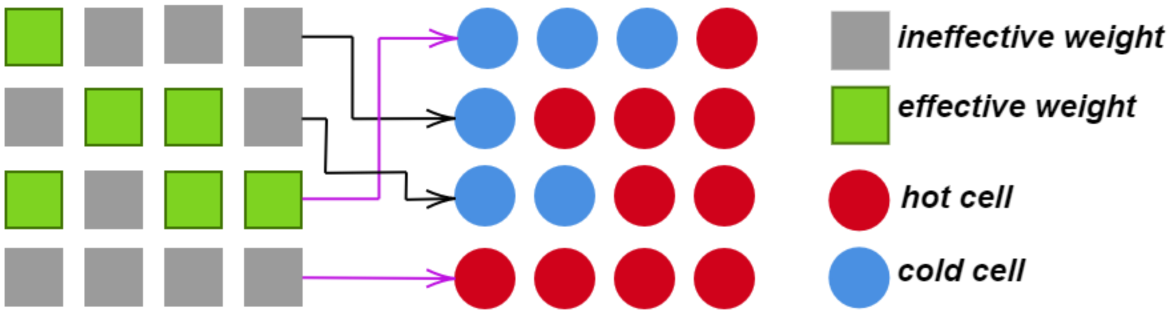

Supplementary Figure 4. Thermal-aware row-adjustment

C) Weight decomposition

The conventional way to map both positive and negative weights of a neural network on memristor crossbar array is to split it to two crossbar arrays in a way that the absolute value of a positive weight is stored in positive array W^{pos} and the absolute value of a negative weights is stored in a negative array W^{neg}. Then, analog subtraction of the values is done. There are $2^N - V$ ways to decompose N bit value V into W^{pos} and W^{neg}. Temperature of a memristor crossbar array is proportional to the applied voltage. Besides, the sum of partial weights stored in each cell is proportional to the applied voltage too. Therefore, the thermal-aware decomposition strategy aims to find the case with the smallest sum of partial weights:

$$Cost(V,k) = \sum_{i=1}^{N_c} \left(\omega_i^{pos}(V,k) + \omega_i^{neg}(V,k) \right) \qquad (SI-3)$$

$$\left(W_{V,opt}^{pos}, W_{V,opt}^{neg} \right) = \arg\min_{k} Cost(V,k) \qquad (SI-4)$$

Here, k is the decomposition case for weight value V; $\omega_i^{pos}(V,k)$ and $\omega_i^{neg}(V,k)$ i^{th} positive and negative partial weights of the kth decomposition case for weight V.

To reduce temperature in crossbar array, the weight decomposition technique called TOPAR I generates all possible decomposition cases with corresponding cost $Cost(V,k)$. For example, if a weight value V=15 should be represented as an 8-bit value using four 2-bit memristor cells, there are 241 decomposition cases such as (15,0), (16,1), (17,2), and so on. Based on Equation 3, it can be found that the decomposition case (16,1) that can be represented using eight 2-bit cells 00,01,00,00,00,00,00,01 has the minimum cost $Cost(16,1) = 2$ which is also illustrated in Supplementary Figure 5.

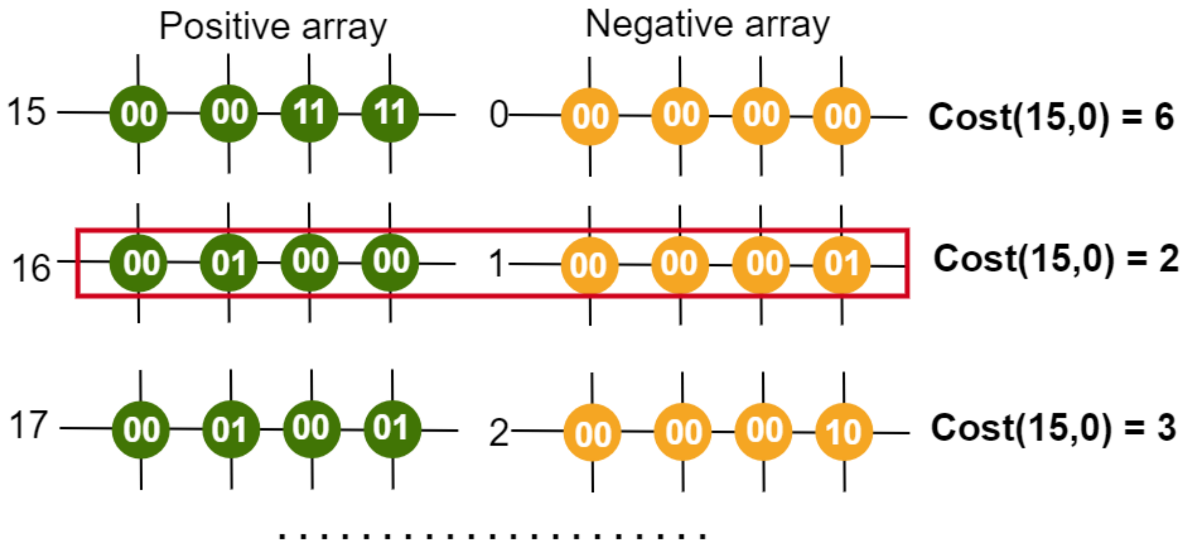

Supplementary Figure 5. Weight decomposition with the lowest temperature

D) Thermal-aware column re-ordering

Temperature-aware column re-ordering is typically used after weight decomposition. It aims to reduce the temperature difference between arrays by shuffling the order of pairs. Since a single weight is represented by a pair of two weights located at W^{pos} and W^{neg}, column re-ordering should take this into consideration and therefore difference between positives and negative arrays of i_{th} re-ordering pair:

$$Diff_i = \sum_{n=1}^{N_c} \omega_n^{pos} - \sum_{n=1}^{N_c} \omega_n^{neg} \qquad (SI-5)$$

where $N_c$the number of crossbar elements; $\omega_i^{pos}(V,k)$ and $\omega_i^{neg}(V,k)$ are n_{th} partial weight in positive and negative array of each pair. Column re-ordering is an optimization problem that aims to minimize the sum of the partial weights in each array $Cost^{arr}$ as shown in Supplementary Figure 6.

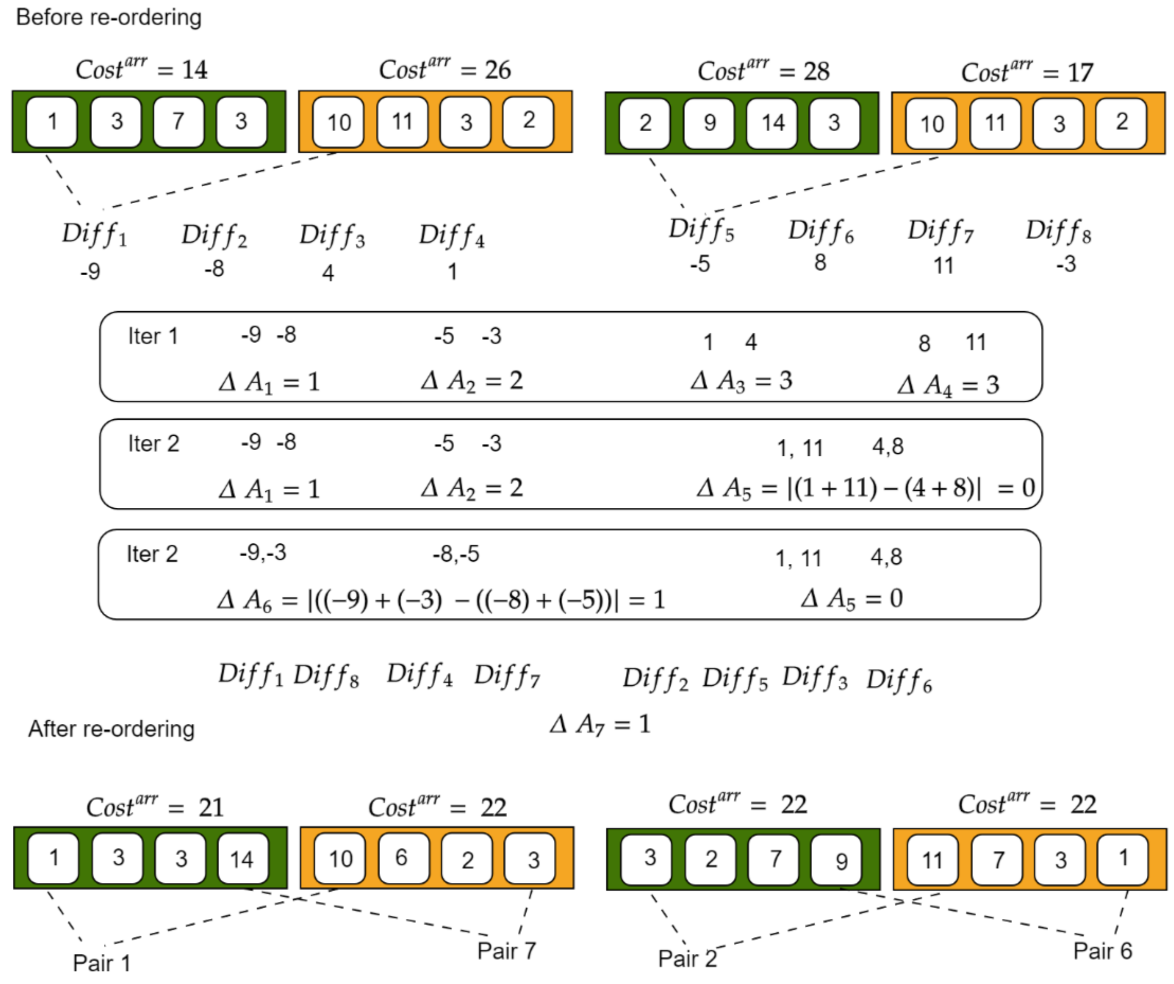

Supplementary Figure 6. Column re-ordering to reduce temperature variance between arrays

E) Fine-grained weight adjustment

There are cases when several weight decomposition cases have the same minimum cost $Cost(V,k)$. However, since the weights are stored in separate W^{pos} and W^{neg} arrays, temperature of the array may depend on the chosen decomposition case. The so-called fine-grained weight adjustment scheme uses a greedy algorithm and is applied after column re-ordering to reduce the temperature variance between W^{pos} and W^{neg} arrays considering $Cost^{pos}(V,k)$ and $Cost^{neg}(V,k)$. For example, decomposition cases (10,0) and (16, 6) both have cost equal to 4 (Supplementary Figure 7). Among these two cases, we select the pair that reduces $Cost^{pos}(V,k)$ and $Cost^{neg}(V,k)$. If $Cost^{pos} = 16$ and $Cost^{neg} = 19$, the decomposition case (10,0) has the minimum cost $|Cost^{pos}(V,k)$ - $Cost^{neg}(V,k)|$ = |(16+4)-(19+0)| =1.

F) Bit-width downgrading

The bit-width downgrading scheme [19] is applied if the sensed temperature of a memristor exceeds the threshold temperature of 330 ºK. A typical weight to conductance encoding scheme is explained in detail in section 2.3 and equation 4 from the main text. Yet, for the sake of clarity we can re-write it as:

$$G = \alpha * W + \beta \qquad (SI-6)$$

where $\alpha = \frac{G_{max}+G_{min}}{w_{max}-w_{min}}$ and $\beta = G_{max} - \alpha * w_{max}$. To downgrade the bit width of a hot cell a new conductance value is calculated:

$$G_{new} = \frac{1}{2^N} * (\alpha * W + \beta) \qquad (SI-7)$$

where N represents the number of bits shifted right. Then a memristor states should be encoded with a new conductance value. In [] when downgrade bit signal is obtained, there are two cases. If it makes change from 0 to 1, the system recomputed the conductance with N bit shift and re-programmes the cells. If change is made from 1 to 0, the system performs weight restoration and programmes original weights to memristors. If bit downgrading took place, output of crossbar array is shifted N bits back using shift-and-add unit.

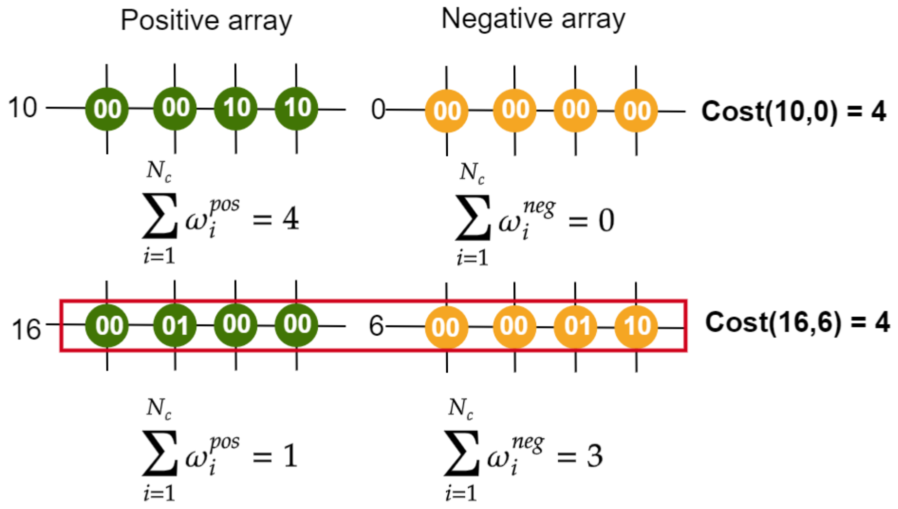

Supplementary Figure 7. Fine-grained weight adjustment

G) Tile-pairing

The idea of tile pairing is pairing overheated "master" tile with cooled-down "slave" pair to reduce the power consumption in the hot tile [19]. The unpaired tile is considered overheated in case that 80% of the crossbar's memristors reach the threshold temperature. Crossbar arrays in both master and slave tiles use the same weights G_{ij} and same input V_i. The output of master crossbar array is $V_{out}^m = \{v_0, v_{2,..,} v_{2N}\}$ and the slave crossbar is $V_{out}^s = \{v_1, v_{3,..,} v_{2N+1}\}$, respectively. The output voltage can be found as:

$$V_o = V_i^T \times G_{new} \times R_s \times 2^N = \left(V_i^T \times G_{old} \times \frac{R_s}{2^N}\right) \times 2^N \qquad (SI-8)$$

H) Temperature-aware weights remapping in subarrays

According to [21], shallow layers (L2-L4) of a VGG-11 neural network model have more impact on the final result than deep layers (L8-L10). To preserve the accuracy of neural model, the weights of shallow layers should be mapped on sub-arrays with lower temperature. To implement weights re-mapping on crossbar array a special placement strategy is used [21]. Therefore, weights of each layer are divided into smaller weights subsets to fit the size of a subarray and the required number of subarray is calculated. Then, temperature of each subarray is summed and stored. The aim of placement function is to place adjacent weights subsets as close as possible to reduce interconnection delays with consideration of a generated thermal heat map of crossbar array. When the function is applied, a "weight map" with the lowest temperature distribution is generated.

I) Weight pruning and splitting (WPS) in subarrays

Weight pruning and splitting technique is used to reduce temperature of hot subarrays in the cases when the accuracy is below a threshold Thr_{Acc} (determined by a designer) and weight remapping did not help [21]. Decision on which weights should be pruned is made based on criticality parameter. Since neural network layers have different impact on the final output, pruning ratio *Prune_ratio* of first layers should be smaller than the ratio of the deep layers. Besides, weights of each layer i have their own level of importance. The criticality of each subset is evaluated using absolute sum of the weights and subsets are sorted in a list based on their criticality level from high to low. First half of the list is classified as "critical" and the other half as "less-critical". Pruning top critical weights has more negative impact on the accuracy than pruning less-critical weights. After each pruning iteration δ is subtracted from a initial *Prune_ratio=80%* and repeated until the obtained accuracy is higher than Thr_{Acc} . Pruning generates unused subarrays and decreases power consumption, but also decreases the accuracy. Therefore, the pruned model is retrained and a new weight map is generated. Splitting of critical weights to lower conductance values is applied in the case of the sufficient number of unused subarrays. For instance, an 8-bit weight is mapped to two 4-bit memristor cells. For the decimal value V=236 , cells should store 14 (1110) and 12 (1100). However, due to high temperature both cells store 8 (1000) that corresponds to a wrong V=136. Therefore, splitting conductance level will allow to restore the value V=236 using four 4-bit cells and lower half of the conductance range corresponding to 8, 6, 8 and 4 as shown in Supplementary Figure 8.

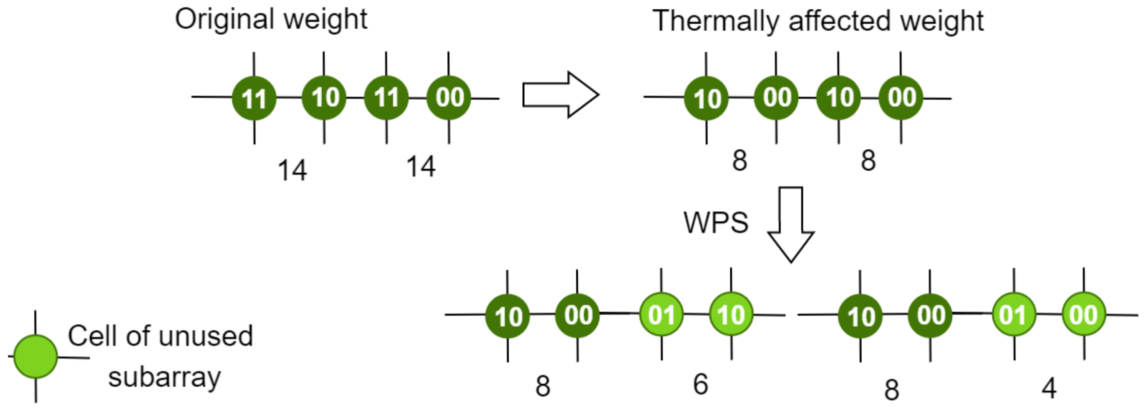

Supplementary Figure 8. Weight pruning and splitting

J) Weight compensation (WC) in subarrays

It is known that temperature affects weights with higher conductance. One of the ways to reduce weight value is shifting it right and restoring the output by shifting it left as was suggested in bitwidth downgrading in [19]. For example, the weight value V=7 and input voltage is 1. If three MSB levels are affected by hight temperature, they can be shifted right, and the weight value becomes V=4. The multiplication output is equal to 4 and shifted left giving compensated value V=8 which is close to 7. The use of such weight compensation techniques is suggested only if weight remapping and WPS methods did not help [21].

Supplementary Algorithm 2. Code of the *k*-fold cross-validation for MATLAB. Note that the part of the code that performs the training in each iteration is identical to that one used for training the SLP, except that in this case, the parameters **`trainFcn`**, **`trainRatio`** and **`testRatio`** are passed as arguments to the training code shown in Algorithm 3

```
%% This part performs the k-fold analysis.
train_functions={'trainlm',...      %Levenberg-Marquardt
                 'trainbfg',...     %BFGS Quasi-Newton
                 'trainrp',...      %Resilient Backpropagation
                 'trainscg',...     %Scaled Conjugate Gradient
                 'traincgb',...     %Conjugate Gradient with Powell/Beale Restarts
                 'traincgf',...     %Fletcher-Powell Conjugate Gradient
                 'traincgp',...     %Polak-Ribiére Conjugate Gradient
                 'trainoss',...     %One Step Secant
                 'traingdx',...     %Variable Learning Rate Gradient Descent
                 'traingdm',...     %Gradient Descent with Momentum
                 'traingd'};        %Gradient Descent

k_folds=5;                                     % number of groups to divide de dataset
k_fold_iterations=10;                          % number of k-fold repeats
for trainFcn_i=1:length(train_functions)       % this iterates over train functions
    for kf_it=1:k_fold_iterations              % this iterates over the repeats
        tStart = cputime                       % starts measuring the training time
        train_results=train_MLP_fcn([8 8],...           % See Algorithm 3. Same code
                              [64 10],...         % but takes trainRatio, testRatio
                              'valChecks',20,... % and trainFcn as arguments
                              'save?',0,...
                              'trainRatio',(100/(k_folds-1))*k_folds;
                              'testRatio',100/(k_folds-1);
                              'trainFcn',train_functions{trainFcn_i});
        tStop = cputime - tStart               % saves the training time
        accuracy_matrix(trainFcn_i,kf_it)=train_results.Accuracy; % stores the accuracy
        trained_nets{trainFcn_i,kf_it}=train_results.A;           % stores the network
        time_methods(trainFcn_i,kf_it)=train_results.tStop;       % stores the train time
    end
end
```

Supplementary Algorithm 3. Creation and training of a SLP for the classification of the MNIST images rescaled to 8×8 pixels (SLP of size 64×10). The network is created as a MATLAB net object, which includes properties such as the network number of inputs, biases, synaptic weights, neurons activation function, database distribution among test, train and validation samples.

```
%% This part creates and trains the neural network.
x = images_8x8;              % For simplicity the train dataset is stored in variable x
t = labels_8x8;              % For simplicity the test dataset is stored in variable t
t_vec=full(ind2vec(t+1,10)); % The row vector t of 1x10000 is turned into a sparse zero
                             % matrix of 10x10000, with each column having only one non
                             % zero element corresponing to the indicated class

% Network creation using function 'patternnet(). First argument
% indicate the number of output classes (and thereby the number of net
% outputs) and the second argument the training algorithm, in this case
% SCG. Produced network is a MATLAB net object called 'net'
net=patternnet(10,'trainscg');

% Network configuration: number of inputs, biases, neurons's activation functions, etc.
net.inputs{1,1}.size=8*8;                 % This sets the number of inputs of the ANN
net.layers{2,1}.size=10;                  % This sets the number of outputs of the ANN
net.LW{2,1}=diag(ones(10,1));             % As the patternet function adds a second
net.layerweights{2,1}.learn=0;            % synaptic layer connected to a poolmax
                                          % neural layer, these lines force it not to
                                          % play a part in the trainig-test
net.inputweights{1,1}.learnFcn='learncon';
net.layers{1,1}.transferFcn='purelin';    % Sets the transfer function of the output
                                          % neuronal layer to mimic the CPA.
net.biases{1,1}.learn=0;                  % It is important to force the
net.biases{2,1}.learn=0;                  % biases not to be trained and to be kept
net.biasConnect=[0;0];                    % equal to 0 and constant to mimic the CPA

% Dataset partitioning
net.divideFcn='dividerand';               % Instruct the dataset to be randomly devided
net.divideMode='sample';                  % intro train, and validation chunks.
net.divideParam.trainRatio=80/100;        % 80% is used for tranining
net.divideParam.valRatio=20/100;          % 20% is used for validation
net.divideParam.testRatio=0/100;

% Tranining settings
net.trainParam.max_fail=15;       % Sets the output conditions for the traning procedure,
net.trainParam.min_grad=1e-7;     % in this case it is stopped after 15 correct
                                  % validations or a gradient below 1e-7
[net,tr_a] = train(net,x,t_vec);  % Network Training using the train() function and the
                                  % dataset x andlabels t_vec
```

Supplementary Algorithm 4. MATLAB code that solves the transport equation to determine the value of the control parameter λ for each of the memdiode devices.

```
for i=1:2                    % The process is repeated for the positive and negative CPA
    for G_idx2=1:10          % This iterates over the rows of the 8*8x10 CPA
        for G_idx1=1:8*8     % This iterates over the columns of the 8*8x10 CPA
            % This defines an equation of the form I_ij=I(V,lambda_ij), where lambda_ij
            % is the variable to find. V is the read voltage. I_ij is calculated as
            % g_ij*V
            eqn= I_matrix(G_idx1,G_idx2,i)==(Imax*lambda+Imin*(1-
                lambda))*(exp(beta*(alphamax*lambda+alphamin*(1-lambda))*(V-
                ((rsmax*lambda+rsmin*(1-lambda)))*I_matrix(G_idx1,G_idx2,i)))-exp(-(1-
                beta)*(alphamax*lambda+alphamin*(1-lambda))*(V-((rsmax*lambda+rsmin*(1-
                lambda)))*I_matrix(G_idx1,G_idx2,i))));
            % This solves equation eqn for variable lambda using the function vpasolve,
            % and stores the lambda value in the matrix W_init, in the position
            %(G_idx1,G_idx2,i);
            W_init(G_idx1,G_idx2,i)=double(vpasolve(eqn,lambda));
        end
    end
end
```

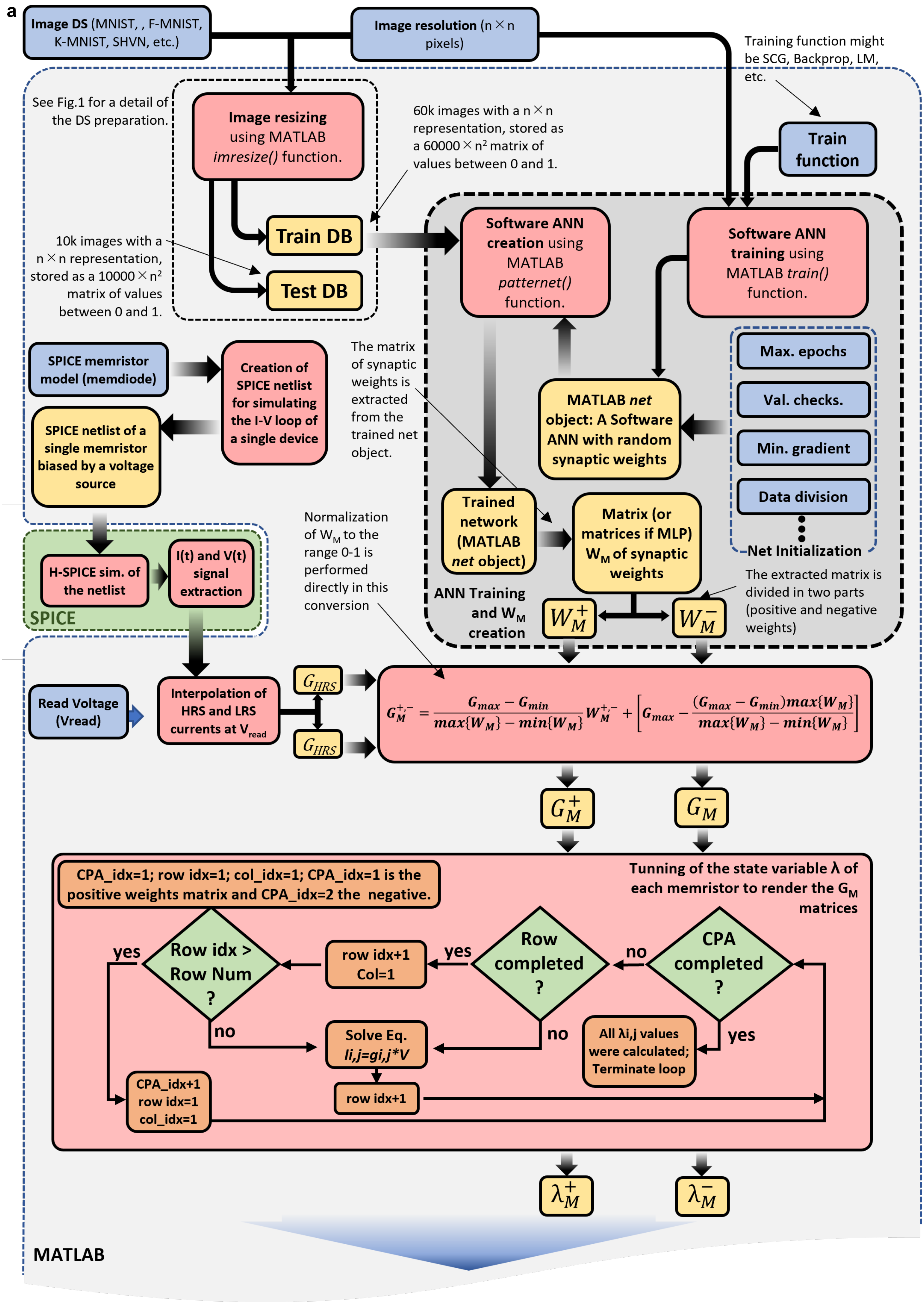

a
Image DS (MNIST, , F-MNIST, K-MNIST, SHVN, etc.)
Image resolution (n × n pixels)
Training function might be SCG, Backprop, LM, etc.
Train function
See Fig.1 for a detail of the DS preparation.
Image resizing using MATLAB imresize() function.
60k images with a n × n representation, stored as a 60000 × n² matrix of values between 0 and 1.
Train DB
Test DB
10k images with a n × n representation, stored as a 10000 × n² matrix of values between 0 and 1.
Software ANN creation using MATLAB patternet() function.
Software ANN training using MATLAB train() function.
SPICE memristor model (memdiode)
Creation of SPICE netlist for simulating the I-V loop of a single device
SPICE netlist of a single memristor biased by a voltage source
The matrix of synaptic weights is extracted from the trained net object.
MATLAB net object: A Software ANN with random synaptic weights
Max. epochs
Val. checks.
Min. gradient
Data division
Net Initialization
Trained network (MATLAB net object)
Matrix (or matrices if MLP) W_M of synaptic weights
H-SPICE sim. of the netlist
I(t) and V(t) signal extraction
SPICE
Normalization of W_M to the range 0-1 is performed directly in this conversion
ANN Training and W_M creation
The extracted matrix is divided in two parts (positive and negative weights)
Read Voltage (Vread)
Interpolation of HRS and LRS currents at V_read
G_HRS
G_HRS
$G_M^{+,-} = \frac{G_{max} - G_{min}}{max\{W_M\} - min\{W_M\}} W_M^{+,-} + \left[G_{max} - \frac{(G_{max} - G_{min}) max\{W_M\}}{max\{W_M\} - min\{W_M\}} \right]$
G_M^+
G_M^-
CPA_idx=1; row idx=1; col_idx=1; CPA_idx=1 is the positive weights matrix and CPA_idx=2 the negative.
Tunning of the state variable λ of each memristor to render the G_M matrices
yes
Row idx > Row Num ?
row idx+1 Col=1
yes
Row completed ?
no
CPA completed ?
no
no
Solve Eq. Ii,j=gi,j*V
All λi,j values were calculated; Terminate loop
yes
CPA_idx+1 row idx=1 col_idx=1
row idx+1
λ_M^+
λ_M^-
MATLAB

b

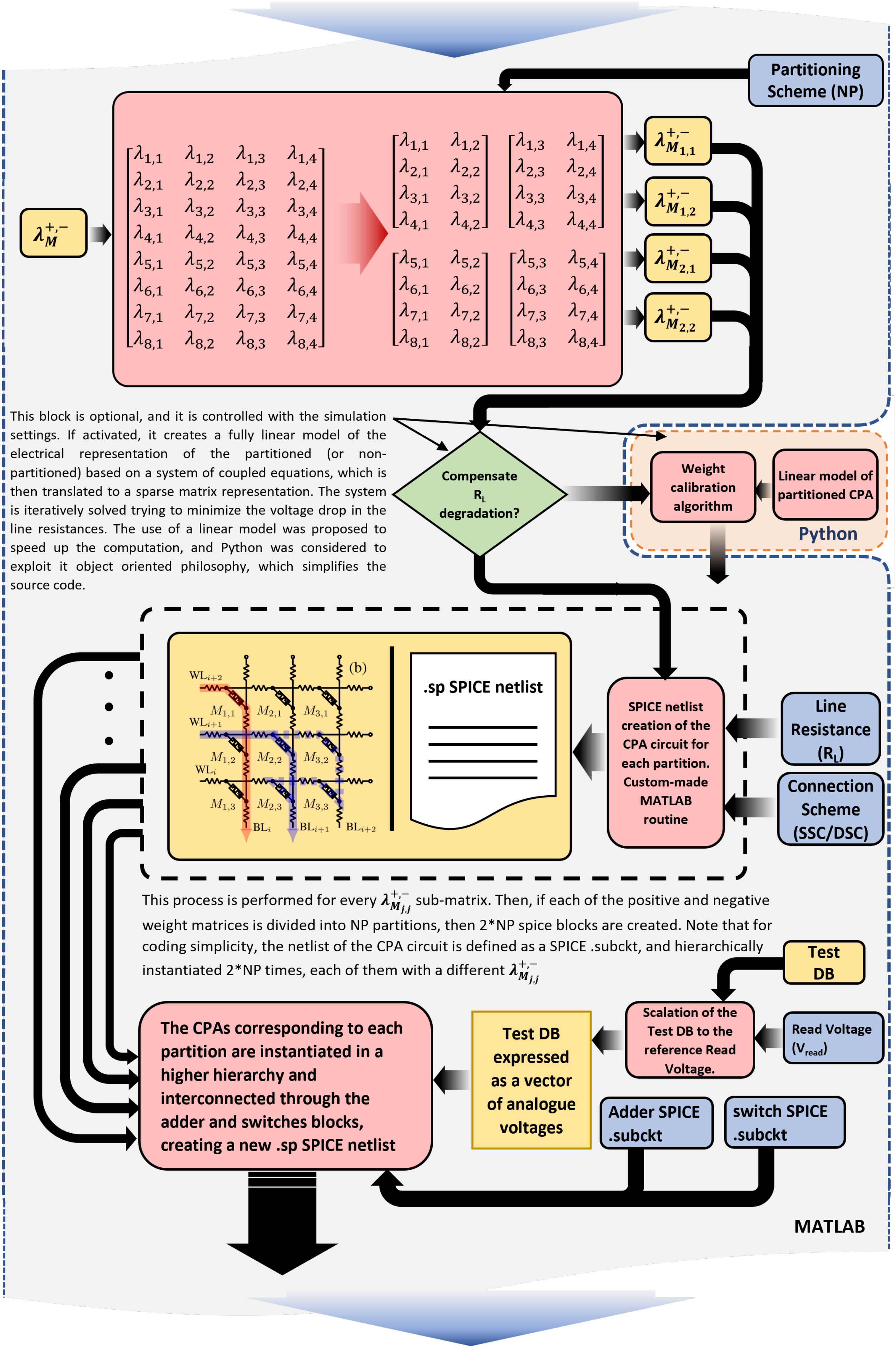
Partitioning Scheme (NP)
$\lambda_M^{+,-}$
$\lambda_{M_{1,1}}^{+,-}$
$\lambda_{M_{1,2}}^{+,-}$
$\lambda_{M_{2,1}}^{+,-}$
$\lambda_{M_{2,2}}^{+,-}$
This block is optional, and it is controlled with the simulation settings. If activated, it creates a fully linear model of the electrical representation of the partitioned (or non-partitioned) based on a system of coupled equations, which is then translated to a sparse matrix representation. The system is iteratively solved trying to minimize the voltage drop in the line resistances. The use of a linear model was proposed to speed up the computation, and Python was considered to exploit it object oriented philosophy, which simplifies the source code.
Compensate R_L degradation?
Weight calibration algorithm
Linear model of partitioned CPA
Python
.sp SPICE netlist
SPICE netlist creation of the CPA circuit for each partition. Custom-made MATLAB routine
Line Resistance (R_L)
Connection Scheme (SSC/DSC)
This process is performed for every $\lambda_{M_{j,j}}^{+,-}$ sub-matrix. Then, if each of the positive and negative weight matrices is divided into NP partitions, then 2*NP spice blocks are created. Note that for coding simplicity, the netlist of the CPA circuit is defined as a SPICE .subckt, and hierarchically instantiated 2*NP times, each of them with a different $\lambda_{M_{j,j}}^{+,-}$
Test DB
Scalation of the Test DB to the reference Read Voltage.
Read Voltage (V_{read})
Test DB expressed as a vector of analogue voltages
The CPAs corresponding to each partition are instantiated in a higher hierarchy and interconnected through the adder and switches blocks, creating a new .sp SPICE netlist
Adder SPICE .subckt
switch SPICE .subckt
MATLAB

c

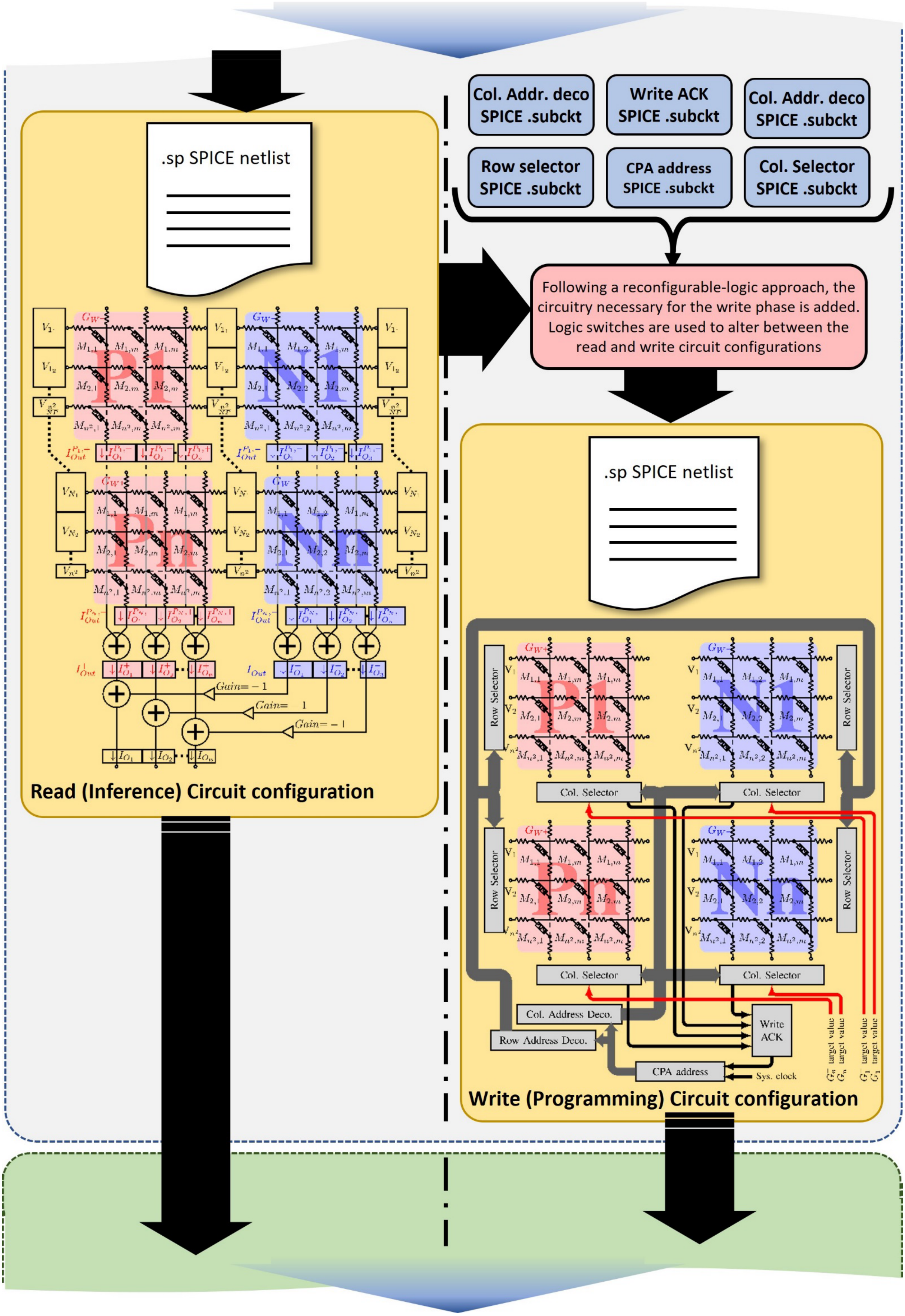
.sp SPICE netlist
Read (Inference) Circuit configuration
Col. Addr. deco SPICE .subckt
Write ACK SPICE .subckt
Col. Addr. deco SPICE .subckt
Row selector SPICE .subckt
CPA address SPICE .subckt
Col. Selector SPICE .subckt
Following a reconfigurable-logic approach, the circuitry necessary for the write phase is added. Logic switches are used to alter between the read and write circuit configurations
.sp SPICE netlist
Row Selector
Col. Selector
Col. Address Deco.
Row Address Deco.
CPA address
Write ACK
Sys. clock
Write (Programming) Circuit configuration

d

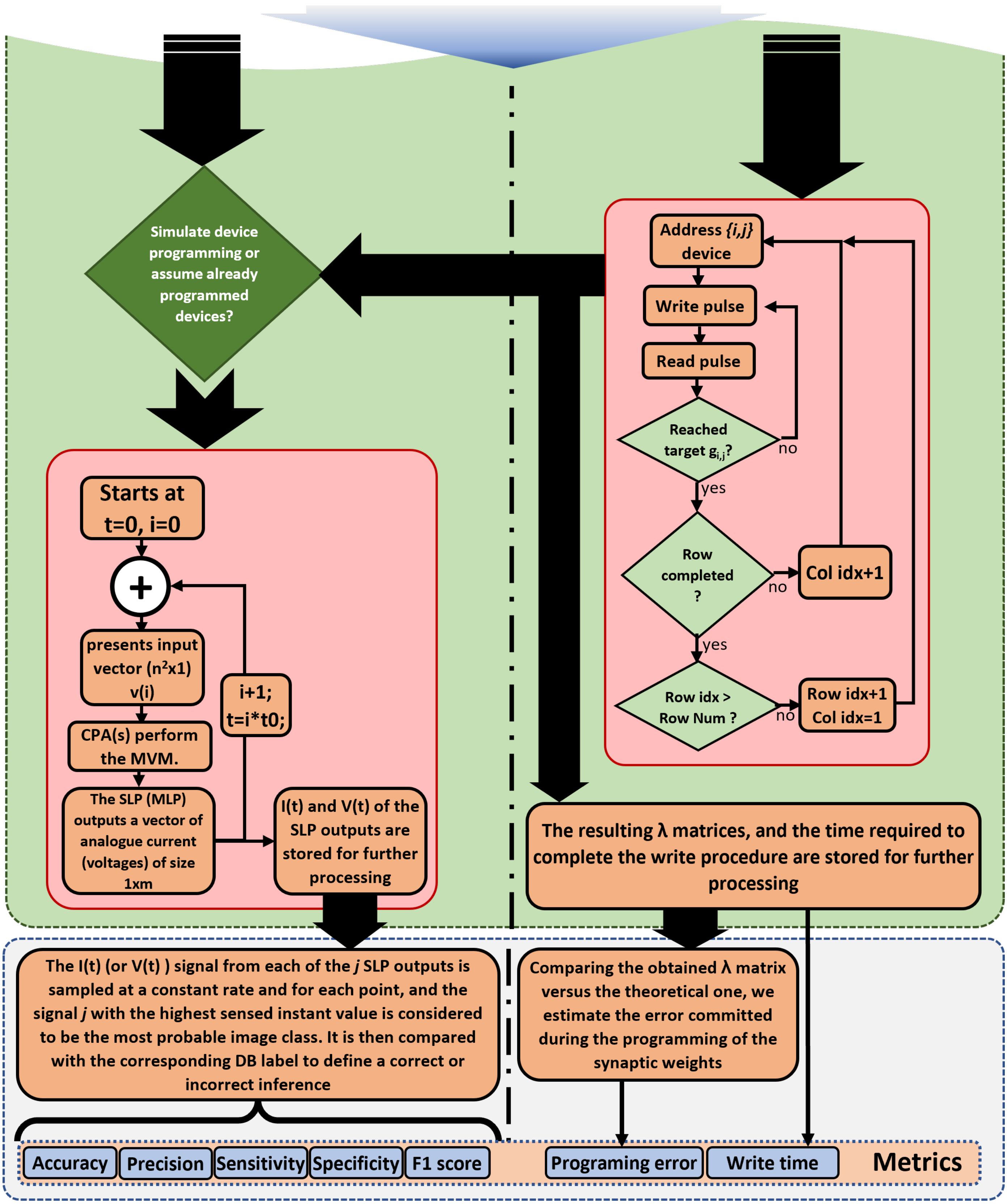

Supplementary Figure 9. (a) Database creation and ideal software ANN generation and training. The last phase shows the computation of the memristors conductance values. (b) Partitioning of the matrix of synaptic weights (or control parameters λ). The possible correction of these values is performed at this point (if required) and the resulting values of the control parameter are then passed to the routine than creates the SPICE netlist for each partition, considering the line resistance, RRAM model, and connection scheme. (c) Partitions are combined to render the full circuit. Reconfigurable logic is added to switch between inference and write configurations. (d) Weight programming and inference simulations. Metrics extraction.

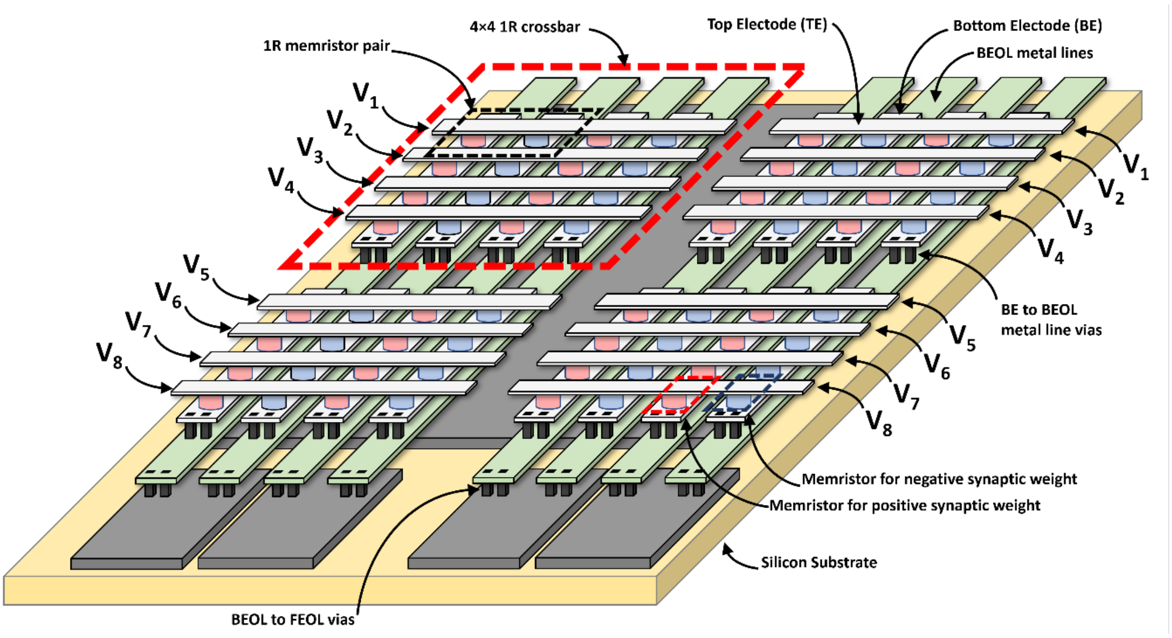

Supplementary Figure 10. Simplified sketch for a partitioned crossbar-array based single layer perceptron. The crossbar array is subdivided into N identically sized partitions to minimize the parasitic voltage drops and placed in the Back-End-Of-Line (BEOL). The circuit peripherals could potentially be placed below them, in the Front-End-Of-Line (FEOL). Partial output current vectors are indicated in the output of each partition.

Supplementary Algorithm 5. SPICE netlist of a 4×4 RRAM-based single layer perceptron implemented using 2 partitions for both the positive and negative crossbars of memristor arrays. The netlist include the CMOS blocks required for the inference as well as the and write phase.

```
******************************************************************
******************************************************************
******************************************************************
******************************************************************

.global gnd! vdd! vss!
.option search='/home/Tecnologias/IBM_8RF/kit/analog/hspice/models'
.option INGOLD=1
.option RUNLVL=1
.option OPFILE=1
.option SPLIT_DP=1
.option MEASFORM=1
.option CSDF=1
.option SYMB=1
.option RELTOL=0.01
.option METHOD=TRAP
.option DELMAX=10u
.option LIST=1
.option LIS_NEW=1
.option POST=CSDF
.option finesim_print_to_probe=1
.option finesim_output=fsdb
.temp 25
.lib 'allModels.inc' tt
.include 'design.inc'
.inc "/home/users/aguirref/nn_rs_uab/models/std_cells/ibm13rfrtv/ibm13rfrvt_XOR2X4TF.sp"
.inc "/home/users/aguirref/nn_rs_uab/models/std_cells/ibm13rfrtv/ibm13rfrvt_DFFSRX4TF.sp"
.inc "/home/users/aguirref/nn_rs_uab/models/std_cells/ibm13rfrtv/ibm13rfrvt_BUFX4TF.sp"
.inc "/home/users/aguirref/nn_rs_uab/models/std_cells/ibm13rfrtv/ibm13rfrvt_INVX4TF.sp"
.inc "/home/users/aguirref/nn_rs_uab/models/std_cells/ibm13rfrtv/ibm13rfrvt_OR2X4TF.sp"
.inc "/home/users/aguirref/nn_rs_uab/models/std_cells/ibm13rfrtv/ibm13rfrvt_AND2X4TF.sp"
.inc "/home/users/aguirref/nn_rs_uab/models/std_cells/ibm13rfrtv/ibm13rfrvt_AND3X4TF.sp"
.inc "/home/users/aguirref/nn_rs_uab/models/std_cells/ibm13rfrtv/ibm13rfrvt_AND4X4TF.sp"
.inc "/home/users/aguirref/nn_rs_uab/models/std_cells/ibm13rfrtv/ibm13rfrvt_neuron.sp"
.inc "/home/users/aguirref/nn_rs_uab/models/memdiode/new_version/memdiode_HSPICE_new_C2.sp"

*####################  Resistive memory array SUBCKT  ###################
.subckt MEMD_network_2_by_4 r1_PAD_1 r2_PAD_1 c1_PAD_1 c2_PAD_1 c3_PAD_1 c4_PAD_1
*First layer interconnect lines with series resistance
Rr1_PAD_1 r1_PAD_1 r1-1 R='Rs_PAD*n_extra'
Rr1_1_2 r1-1 r1-2 R='Rs'
Cr1_1_up r1-1 gnd! C='C_line2gnd'
Rr1_2_3 r1-2 r1-3 R='Rs'
Cr1_2_up r1-2 gnd! C='C_line2gnd'
Rr1_3_4 r1-3 r1-4 R='Rs'
Cr1_3_up r1-3 gnd! C='C_line2gnd'
Rr1_PAD_2 r1-4 r1_PAD_1 R='Rs_PAD*n_extra'
Rr2_PAD_1 r2_PAD_1 r2-1 R='Rs_PAD*n_extra'
Rr2_1_2 r2-1 r2-2 R='Rs'
Cr2_1_up r2-1 gnd! C='C_line2gnd'
Cr2_1_1 2-1 1-1 C='C_interline'
Rr2_2_3 r2-2 r2-3 R='Rs'
Cr2_2_up r2-2 gnd! C='C_line2gnd'
Cr2_2_2 2-2 1-2 C='C_interline'
Rr2_3_4 r2-3 r2-4 R='Rs'
Cr2_3_up r2-3 gnd! C='C_line2gnd'
Cr2_3_3 2-3 1-3 C='C_interline'
Rr2_PAD_2 r2-4 r2_PAD_1 R='Rs_PAD*n_extra'
**************************************************************************
*Second layer interconnect lines with series resistance
Rc1_PAD_1 c1_PAD_1 c1-1 R='Rs_PAD'
Rc1_1_2 c1-1 c1-2 R='Rs'
Cc1_1_low c1-1 gnd! C='C_line2gnd'
Rc1_PAD_2 c1-2 c1_PAD_2 R='Rs_PAD'
Rc1_PAD_3 c1_PAD_2 gnd! R='HZ'
Rc2_PAD_1 c2_PAD_1 c2-1 R='Rs_PAD'
Rc2_1_2 c2-1 c2-2 R='Rs'
Cc2_1_low c2-1 gnd! C='C_line2gnd'
Cc2_1_1 c2-1 c1-1 C='C_interline'
Rc2_PAD_2 c2-2 c2_PAD_2 R='Rs_PAD'
```

```
Rc2_PAD_3 c2_PAD_2 gnd! R='HZ'
Rc3_PAD_1 c3_PAD_1 c3-1 R='Rs_PAD'
Rc3_1_2 c3-1 c3-2 R='Rs'
Cc3_1_low c3-1 gnd! C='C_line2gnd'
Cc3_1_1 c3-1 c2-1 C='C_interline'
Rc3_PAD_2 c3-2 c3_PAD_2 R='Rs_PAD'
Rc3_PAD_3 c3_PAD_2 gnd! R='HZ'
Rc4_PAD_1 c4_PAD_1 c4-1 R='Rs_PAD'
Rc4_1_2 c4-1 c4-2 R='Rs'
Cc4_1_low c4-1 gnd! C='C_line2gnd'
Cc4_1_1 c4-1 c3-1 C='C_interline'
Rc4_PAD_2 c4-2 c4_PAD_2 R='Rs_PAD'
Rc4_PAD_3 c4_PAD_2 gnd! R='HZ'
*************************************************************************
*Resistive memory devices connecting neurons
Rcs_r1-1c1-1_1 r1-1 r1-1_p R='Rcs'
XMEMDr1-1c1-1 r1-1_p c1-1_n memdiode H0='H0_r1_c1'
CMEMDr1-1c1-1 r1-1_p c1-1_n memdiode C='C_memdiode'
Rcs_r1-1c1-1_2 c1-1_n c1-1 R='Rcs'
Rcs_r1-2c2-1_1 r1-2 r1-2_p R='Rcs'
XMEMDr1-2c2-1 r1-2_p c2-1_n memdiode H0='H0_r1_c2'
CMEMDr1-2c2-1 r1-2_p c2-1_n memdiode C='C_memdiode'
Rcs_r1-2c2-1_2 c2-1_n c2-1 R='Rcs'
Rcs_r1-3c3-1_1 r1-3 r1-3_p R='Rcs'
XMEMDr1-3c3-1 r1-3_p c3-1_n memdiode H0='H0_r1_c3'
CMEMDr1-3c3-1 r1-3_p c3-1_n memdiode C='C_memdiode'
Rcs_r1-3c3-1_2 c3-1_n c3-1 R='Rcs'
Rcs_r1-4c4-1_1 r1-4 r1-4_p R='Rcs'
XMEMDr1-4c4-1 r1-4_p c4-1_n memdiode H0='H0_r1_c4'
CMEMDr1-4c4-1 r1-4_p c4-1_n memdiode C='C_memdiode'
Rcs_r1-4c4-1_2 c4-1_n c4-1 R='Rcs'
Rcs_r2-1c1-2_1 r2-1 r2-1_p R='Rcs'
XMEMDr2-1c1-2 r2-1_p c1-2_n memdiode H0='H0_r2_c1'
CMEMDr2-1c1-2 r2-1_p c1-2_n memdiode C='C_memdiode'
Rcs_r2-1c1-2_2 c1-2_n c1-2 R='Rcs'
Rcs_r2-2c2-2_1 r2-2 r2-2_p R='Rcs'
XMEMDr2-2c2-2 r2-2_p c2-2_n memdiode H0='H0_r2_c2'
CMEMDr2-2c2-2 r2-2_p c2-2_n memdiode C='C_memdiode'
Rcs_r2-2c2-2_2 c2-2_n c2-2 R='Rcs'
Rcs_r2-3c3-2_1 r2-3 r2-3_p R='Rcs'
XMEMDr2-3c3-2 r2-3_p c3-2_n memdiode H0='H0_r2_c3'
CMEMDr2-3c3-2 r2-3_p c3-2_n memdiode C='C_memdiode'
Rcs_r2-3c3-2_2 c3-2_n c3-2 R='Rcs'
Rcs_r2-4c4-2_1 r2-4 r2-4_p R='Rcs'
XMEMDr2-4c4-2 r2-4_p c4-2_n memdiode H0='H0_r2_c4'
CMEMDr2-4c4-2 r2-4_p c4-2_n memdiode C='C_memdiode'
Rcs_r2-4c4-2_2 c4-2_n c4-2 R='Rcs'
*************************************************************************
*########################################################################
.ends MEMD_network_2_by_4

*#########################  selector SUBCKT  ############################
.subckt selector Out Vcont Write Neuron
gi1 Out Write vcr pwl(1) Vcont gnd! 0.1,100Meg 0.9,0.1
gi2 Neuron Out vcr pwl(1) Vcontn gnd! 0.1,100Meg 0.9,0.1
Xinv2 Vcont Vcontn invx4tf
*########################################################################
.ends selector

*#########################  selector SUBCKT  ############################
.subckt and1x4tf input_1 output
xbufx4tf input_1 output bufx4tf
*########################################################################
.ends and1x4tf

*#########################  selector SUBCKT  ############################
.subckt and5x4tf input_1 input_2 input_3 input_4 input_5 output
xand3x4tf_1 input_1 input_2 input_3 output_1 and3x4tf
xand2x4tf_2 input_4 input_5 output_2 and2x4tf

xand2x4tf_1 output_1 output_2 output and2x4tf

*########################################################################
.ends and5x4tf

*#########################  selector SUBCKT  ############################
.subckt and6x4tf input_1 input_2 input_3 input_4 input_5 input_6 output
xand3x4tf_1 input_1 input_2 input_3 output_1 and3x4tf
xand3x4tf_2 input_4 input_5 input_6 output_2 and3x4tf

```

```
xand2x4tf_1 output_1 output_2 output and2x4tf

*######################################################################
.ends and6x4tf

*#########################  selector SUBCKT  ###########################
.subckt and7x4tf input_1 input_2 input_3 input_4 input_5 input_6 input_7 output
xand4x4tf_1 input_1 input_2 input_3 input_4 output_1 and4x4tf
xand3x4tf_2 input_5 input_6 input_7 output_2 and3x4tf

xand2x4tf_1 output_1 output_2 output and2x4tf

*######################################################################
.ends and7x4tf

*#########################  selector SUBCKT  ###########################
.subckt and8x4tf input_1 input_2 input_3 input_4 input_5 input_6 input_7 input_8 output
xand4x4tf_1 input_1 input_2 input_3 input_4 output_1 and4x4tf
xand4x4tf_2 input_5 input_6 input_7 input_8 output_2 and4x4tf

xand2x4tf_1 output_1 output_2 output and2x4tf

*######################################################################
.ends and8x4tf

*#########################  selector SUBCKT  ###########################
.subckt and12x4tf input_1 input_2 input_3 input_4 input_5 input_6 input_7 input_8 input_9 input_10 input_11 input_12 output
xand4x4tf_1 input_1 input_2 input_3 input_4 output_1 and4x4tf
xand4x4tf_2 input_5 input_6 input_7 input_8 output_2 and4x4tf
xand4x4tf_3 input_9 input_10 input_11 input_12 output_3 and4x4tf

xand3x4tf_1 output_1 output_2 output_3 output and3x4tf

*######################################################################
.ends and8x4tf

*#########################  selector SUBCKT  ###########################
.subckt and15x4tf input_1 input_2 input_3 input_4 input_5 input_6 input_7 input_8 input_9 input_10 input_11 input_12 input_13 input_14 input_15 output
xand4x4tf_1 input_1 input_2 input_3 input_4 output_1 and4x4tf
xand4x4tf_2 input_5 input_6 input_7 input_8 output_2 and4x4tf

xand4x4tf_3 input_9 input_10 input_11 input_12 output_3 and4x4tf
xand3x4tf_4 input_13 input_14 input_15  output_4 and3x4tf

xand4x4tf_5 output_1 output_2 output_3 output_4 output and4x4tf

*######################################################################
.ends and15x4tf

*#########################  selector SUBCKT  ###########################
.subckt and16x4tf input_1 input_2 input_3 input_4 input_5 input_6 input_7 input_8 input_9 input_10 input_11 input_12 input_13 input_14 input_15 input_16 output
xand4x4tf_1 input_1 input_2 input_3 input_4 output_1 and4x4tf
xand4x4tf_2 input_5 input_6 input_7 input_8 output_2 and4x4tf

xand4x4tf_3 input_9 input_10 input_11 input_12 output_3 and4x4tf
xand4x4tf_4 input_13 input_14 input_15 input_16 output_4 and4x4tf

xand4x4tf_5 output_1 output_2 output_3 output_4 output and4x4tf

*######################################################################
.ends and16x4tf

*#########################  selector SUBCKT  ###########################
.subckt and18x4tf input_1 input_2 input_3 input_4 input_5 input_6 input_7 input_8 input_9 input_10 input_11 input_12 input_13 input_14 input_15 input_16 input_17 input_18 output
xand4x4tf_1 input_1 input_2 input_3 input_4 output_1 and4x4tf
xand4x4tf_2 input_5 input_6 input_7 input_8 output_2 and4x4tf

xand4x4tf_3 input_9 input_10 input_11 input_12 output_3 and4x4tf
xand4x4tf_4 input_13 input_14 input_15 input_16 output_4 and4x4tf

xand2x4tf_5 input_17 input_18 output_5 and2x4tf

xand3x4tf_6 output_1 output_2 output_3 output_A and3x4tf

xand2x4tf_7 output_4 output_5 output_B and2x4tf
```

```

xand2x4tf_8 output_A output_B output and2x4tf

*########################################################################
.ends and18x4tf

*#########################  selector SUBCKT  ###########################
.subckt and28x4tf input_1 input_2 input_3 input_4 input_5 input_6 input_7 input_8
input_9 input_10 input_11 input_12 input_13 input_14 input_15 input_16 input_17 input_18
input_19 input_20 input_21 input_22 input_23 input_24 input_25 input_26 input_27
input_28 output

xand4x4tf_1 input_1 input_2 input_3 input_4 output_1 and4x4tf
xand4x4tf_2 input_5 input_6 input_7 input_8 output_2 and4x4tf

xand4x4tf_3 input_9 input_10 input_11 input_12 output_3 and4x4tf
xand4x4tf_4 input_13 input_14 input_15 input_16 output_4 and4x4tf
xand4x4tf_5 input_17 input_18 input_19 input_20 output_5 and4x4tf
xand4x4tf_6 input_21 input_22 input_23 input_24 output_6 and4x4tf
xand4x4tf_7 input_25 input_26 input_27 input_28 output_7 and4x4tf
xand4x4tf_9 output_1 output_2 output_3 output_4 output_A and4x4tf
xand3x4tf_10 output_5 output_6 output_7 output_B and3x4tf

xand2x4tf_10 output_A output_B output and2x4tf

*########################################################################
.ends and28x4tf

*#########################  selector SUBCKT  ###########################
.subckt and32x4tf input_1 input_2 input_3 input_4 input_5 input_6 input_7 input_8
input_9 input_10 input_11 input_12 input_13 input_14 input_15 input_16 input_17 input_18
input_19 input_20 input_21 input_22 input_23 input_24 input_25 input_26 input_27
input_28 input_29 input_30 input_31 input_32 output

xand4x4tf_1 input_1 input_2 input_3 input_4 output_1 and4x4tf
xand4x4tf_2 input_5 input_6 input_7 input_8 output_2 and4x4tf

xand4x4tf_3 input_9 input_10 input_11 input_12 output_3 and4x4tf
xand4x4tf_4 input_13 input_14 input_15 input_16 output_4 and4x4tf
xand4x4tf_5 input_17 input_18 input_19 input_20 output_5 and4x4tf
xand4x4tf_6 input_21 input_22 input_23 input_24 output_6 and4x4tf
xand4x4tf_7 input_25 input_26 input_27 input_28 output_7 and4x4tf
xand4x4tf_8 input_29 input_30 input_31 input_32 output_8 and4x4tf

xand4x4tf_9 output_1 output_2 output_3 output_4 output_A and4x4tf
xand4x4tf_10 output_5 output_6 output_7 output_8 output_B and4x4tf

xand2x4tf_10 output_A output_B output and2x4tf

*########################################################################
.ends and32x4tf

*#########################  Decoder 2-bits SUBCKT  ############################
.subckt decoder_2_output output_1 output_2 clk PRE_1 clear
Xdff1 clk output_2 output_1 RQn1 clearn PRE_1n dffsrx4tf
RQn1 RQn1 gnd! R=1Meg
Xdff2 clk output_1 output_2 RQn2 clearn vdd! dffsrx4tf
RQn2 RQn2 gnd! R=1Meg
Xinv1 PRE_1 PRE_1n invx4tf
Xinv2 clear clearn invx4tf
*########################################################################
.ends decoder_2_output

*#########################  Decoder 4-bits SUBCKT  ############################
.subckt decoder_4_output output_1 output_2 output_3 output_4 clk PRE_1 clear
Xdff1 clk output_4 output_1 RQn1 clearn PRE_1n dffsrx4tf
RQn1 RQn1 gnd! R=1Meg
Xdff2 clk output_1 output_2 RQn2 clearn vdd! dffsrx4tf
RQn2 RQn2 gnd! R=1Meg
Xdff3 clk output_2 output_3 RQn3 clearn vdd! dffsrx4tf
RQn3 RQn3 gnd! R=1Meg
Xdff4 clk output_3 output_4 RQn4 clearn vdd! dffsrx4tf
RQn4 RQn4 gnd! R=1Meg
Xinv1 PRE_1 PRE_1n invx4tf
Xinv2 clear clearn invx4tf
*########################################################################
.ends decoder_4_output

*#########################  Counter 2-bit SUBCKT   ############################
.subckt counter_2_pulses clk counter_pulse_out clear
```

```
Xdff1 clk input output_1 RQn1 clearn vdd! dffsrx4tf
RQn1 RQn1 gnd! R=1Meg
Xdff2 clk output_1 output_2 RQn2 clearn vdd! dffsrx4tf
RQn2 RQn2 gnd! R=1Meg
Xxbuf1 output_2 counter_pulse_out bufx4tf
Xinv1 clear clearn invx4tf
X_set clk gnd! inputDFF inputDFF_n vdd! clearn dffsrx4tf
X_or_set inputDFF output_2 input or2x4tf
*######################################################################
.ends counter_2_pulses

*#########################  Counter 4-bit SUBCKT   ###########################
.subckt counter_4_pulses clk counter_pulse_out clear
Xdff1 clk input output_1 RQn1 clearn vdd! dffsrx4tf
RQn1 RQn1 gnd! R=1Meg
Xdff2 clk output_1 output_2 RQn2 clearn vdd! dffsrx4tf
RQn2 RQn2 gnd! R=1Meg
Xdff3 clk output_2 output_3 RQn3 clearn vdd! dffsrx4tf
RQn3 RQn3 gnd! R=1Meg
Xdff4 clk output_3 output_4 RQn4 clearn vdd! dffsrx4tf
RQn4 RQn4 gnd! R=1Meg
Xxbuf1 output_4 counter_pulse_out bufx4tf
Xinv1 clear clearn invx4tf
X_set clk gnd! inputDFF inputDFF_n vdd! clearn dffsrx4tf
X_or_set inputDFF output_4 input or2x4tf
*######################################################################
.ends counter_4_pulses

*#########################  1-input neuron SUBCKT   ###########################
.subckt neuron_1x2_in in1_pos in1_neg neuron_output ref_node

Rsense_1_pos in1_pos ref_node R='R_in_TIA'

Rsense_1_neg in1_neg ref_node R='R_in_TIA'

Eoutput neuron_output ref_node vol='(v(in1_pos)-v(in1_neg))*100000'
*######################################################################
.ends neuron_1x2_in

*#########################  2-input neuron SUBCKT   ###########################
.subckt neuron_2x2_in in1_pos in2_pos in1_neg in2_neg neuron_output ref_node

Rsense_1_pos in1_pos ref_node R='R_in_TIA'

Rsense_2_pos in2_pos ref_node R='R_in_TIA'

Rsense_1_neg in1_neg ref_node R='R_in_TIA'

Rsense_2_neg in2_neg ref_node R='R_in_TIA'

Eoutput neuron_output ref_node vol='(v(in1_pos)+v(in2_pos)-v(in1_neg)-v(in2_neg))*100000'
*######################################################################
.ends neuron_2x2_in

*#########################  1-input logsig neuron SUBCKT   ###########################
.subckt logsig_neuron_1x2_in in1_pos in1_neg neuron_output ref_node

.param logsig(x)='1/(1+exp(-x))'

Rsense_1_pos in1_pos ref_node R='R_in_TIA'

Rsense_1_neg in1_neg ref_node R='R_in_TIA'

Eoutput neuron_output ref_node vol='0.300000*logsig((v(in1_pos)-v(in1_neg))*100000)' min=0 max=0.300000
*######################################################################
.ends logsig_neuron_1x2_in

*#########################  2-input logsig neuron SUBCKT   ###########################
.subckt logsig_neuron_2x2_in in1_pos in2_pos in1_neg in2_neg neuron_output ref_node

.param logsig(x)='1/(1+exp(-x))'

Rsense_1_pos in1_pos ref_node R='R_in_TIA'

Rsense_2_pos in2_pos ref_node R='R_in_TIA'

Rsense_1_neg in1_neg ref_node R='R_in_TIA'

```

```
Rsense_2_neg in2_neg ref_node R='R_in_TIA'

Eoutput neuron_output ref_node vol='0.300000*logsig((v(in1_pos)+v(in2_pos)-v(in1_neg)-v(in2_neg))*100000)' min=0 max=0.300000
*########################################################################
.ends logsig_neuron_2x2_in

*#########################  1-input tansig neuron SUBCKT   ############################
.subckt tansig_neuron_1x2_in in1_pos in1_neg neuron_output ref_node

.param tansig(x)='2/(1+exp(-2*x))-1'

Rsense_1_pos in1_pos ref_node R='R_in_TIA'

Rsense_1_neg in1_neg ref_node R='R_in_TIA'

Eoutput neuron_output ref_node vol='0.150000*tansig((v(in1_pos)-v(in1_neg))*100000)' min=-0.150000 max=0.150000
*########################################################################
.ends tansig_neuron_1x2_in

*#########################  2-input tansig neuron SUBCKT   ############################
.subckt tansig_neuron_2x2_in in1_pos in2_pos in1_neg in2_neg neuron_output ref_node

.param tansig(x)='2/(1+exp(-2*x))-1'

Rsense_1_pos in1_pos ref_node R='R_in_TIA'

Rsense_2_pos in2_pos ref_node R='R_in_TIA'

Rsense_1_neg in1_neg ref_node R='R_in_TIA'

Rsense_2_neg in2_neg ref_node R='R_in_TIA'

Eoutput neuron_output ref_node vol='0.150000*tansig((v(in1_pos)+v(in2_pos)-v(in1_neg)-v(in2_neg))*100000)' min=-0.150000 max=0.150000
*########################################################################
.ends tansig_neuron_2x2_in

*#########################   1 layer synchronizer SUBCKT  ############################
.subckt sync_W_OK_1_layers WOK_layer_1 output clk PRE_1 clear

Xdff1 clk_en gnd! output_1 RQn1 clearn WOK_layer_1n dffsrx4tf
Xinv1 WOK_layer_1 WOK_layer_1n invx4tf
RQn1 RQn1 gnd! R=1Meg

Xinv_clear clear clearn invx4tf

Xand1 output_1 output and1x4tf
Xdff_gral clk output clk_en clk_en_n clearn vdd! dffsrx4tf
*########################################################################
.ends sync_W_OK_1_layers

*========================================================================================================================================================
*##############################################################  TOP CIRCUIT ###########################################################################

*======================================================================================================================================
*Layers A-B, including interconections (CPA), input and output neurons and programming electronic****************************************************************************************************************************************
*#######################################################################################################################################

*========================================================================
*Layers (A-B) interconnections (CPA): Partition 1-1, pos polarity
*************************************************************************
XMEMD_network_A-B_P1-1_pos AB1_PAD_1_P1-1_pos AB2_PAD_1_P1-1_pos BA1_PAD_1_P1-1_pos BA2_PAD_1_P1-1_pos BA3_PAD_1_P1-1_pos BA4_PAD_1_P1-1_pos MEMD_network_2_by_4 H0_r1_c1=0.443228 H0_r1_c2=0.443228 H0_r1_c3=0.443228 H0_r1_c4=0.443228 H0_r2_c1=0.443228 H0_r2_c2=0.443228 H0_r2_c3=0.443228 H0_r2_c4=0.443228

*========================================================================
*Drivers for the input neurons of intercon. layer A-B (Neural layer A, CPA: 1-1-pos)
*************************************************************************
Xselector_RW_AB1-1-P1-1_pos AB1_PAD_1_P1-1_pos RW_A-B write_signal_AB1_1_P1-1_pos NeuronA1_P1-1 selector
```

```
Xselector_RW_AB1-2-P1-1_pos write_signal_AB1_1_P1-1_pos VcontAB1-1_out_P1-1_pos Vpulsedsignal RHZ_AB1_P1-1_pos selector
VHZ_AB1_P1-1_pos RHZ_AB1_P1-1_pos gnd! dc=0.000000
XandAB1_P1-1_pos VcontA-B1-1 enable_write_A-B_P1-1_pos VcontAB1-1_out_P1-1_pos and2x4tf
VNeuronA1_P1 NeuronA1_P1-1 gnd! PWL(0 0 1.000000000000e-06 0 1.001000000000e-06 0.300000 1.100100000000e-05 0.300000 1.100200000000e-05 0.000000 2.100200000000e-05 0.000000 2.100300000000e-05 0.000000 3.100300000000e-05 0.000000 3.100400000000e-05 0.000000 4.100400000000e-05 0.000000 4.100500000000e-05 0.000000 5.200420000000e-05 0.000000)
************************************************************************
Xselector_RW_AB2-1-P1-1_pos AB2_PAD_1_P1-1_pos RW_A-B write_signal_AB2_1_P1-1_pos NeuronA2_P1-1 selector
Xselector_RW_AB2-2-P1-1_pos write_signal_AB2_1_P1-1_pos VcontAB2-1_out_P1-1_pos Vpulsedsignal RHZ_AB2_P1-1_pos selector
VHZ_AB2_P1-1_pos RHZ_AB2_P1-1_pos gnd! dc=0.000000
XandAB2_P1-1_pos VcontA-B2-1 enable_write_A-B_P1-1_pos VcontAB2-1_out_P1-1_pos and2x4tf
VNeuronA2_P1 NeuronA2_P1-1 gnd! PWL(0 0 1.000000000000e-06 0 1.001000000000e-06 0.000000 1.100100000000e-05 0.000000 1.100200000000e-05 0.300000 2.100200000000e-05 0.300000 2.100300000000e-05 0.000000 3.100300000000e-05 0.000000 3.100400000000e-05 0.000000 4.100400000000e-05 0.000000 4.100500000000e-05 0.000000 5.200420000000e-05 0.000000)
************************************************************************

*=======================================================================
*Drivers for the output neurons of intercon. layer A-B (Neural layer B, CPA: 1-1-pos)
************************************************************************
Xselector_RW_BA1-1-P1-1_pos BA1_PAD_1_P1-1_pos RW_A-B write_signal_BA1_1_P1-1_pos NeuronB1_P1-1-pos selector
Xselector_RW_BA1-2-P1-1_pos write_signal_BA1_1_P1-1_pos VcontBA1-1_out_P1-1_pos comparator_A-B_P1-1_pos RHZ_BA1_P1-1_pos selector
VHZ_BA1_P1-1_pos RHZ_BA1_P1-1_pos gnd! dc=0.650000
XandBA1_P1-1_pos VcontB-A1-1 enable_write_A-B_P1-1_pos VcontBA1-1_out_P1-1_pos and2x4tf
RNeuronB1_P1-1_pos NeuronB1_P1-1-pos gnd! R='R_sense'
************************************************************************
Xselector_RW_BA2-1-P1-1_pos BA2_PAD_1_P1-1_pos RW_A-B write_signal_BA2_1_P1-1_pos NeuronB2_P1-1-pos selector
Xselector_RW_BA2-2-P1-1_pos write_signal_BA2_1_P1-1_pos VcontBA2-1_out_P1-1_pos comparator_A-B_P1-1_pos RHZ_BA2_P1-1_pos selector
VHZ_BA2_P1-1_pos RHZ_BA2_P1-1_pos gnd! dc=0.650000
XandBA2_P1-1_pos VcontB-A2-1 enable_write_A-B_P1-1_pos VcontBA2-1_out_P1-1_pos and2x4tf
RNeuronB2_P1-1_pos NeuronB2_P1-1-pos gnd! R='R_sense'
************************************************************************
Xselector_RW_BA3-1-P1-1_pos BA3_PAD_1_P1-1_pos RW_A-B write_signal_BA3_1_P1-1_pos NeuronB3_P1-1-pos selector
Xselector_RW_BA3-2-P1-1_pos write_signal_BA3_1_P1-1_pos VcontBA3-1_out_P1-1_pos comparator_A-B_P1-1_pos RHZ_BA3_P1-1_pos selector
VHZ_BA3_P1-1_pos RHZ_BA3_P1-1_pos gnd! dc=0.650000
XandBA3_P1-1_pos VcontB-A3-1 enable_write_A-B_P1-1_pos VcontBA3-1_out_P1-1_pos and2x4tf
RNeuronB3_P1-1_pos NeuronB3_P1-1-pos gnd! R='R_sense'
************************************************************************
Xselector_RW_BA4-1-P1-1_pos BA4_PAD_1_P1-1_pos RW_A-B write_signal_BA4_1_P1-1_pos NeuronB4_P1-1-pos selector
Xselector_RW_BA4-2-P1-1_pos write_signal_BA4_1_P1-1_pos VcontBA4-1_out_P1-1_pos comparator_A-B_P1-1_pos RHZ_BA4_P1-1_pos selector
VHZ_BA4_P1-1_pos RHZ_BA4_P1-1_pos gnd! dc=0.650000
XandBA4_P1-1_pos VcontB-A4-1 enable_write_A-B_P1-1_pos VcontBA4-1_out_P1-1_pos and2x4tf
RNeuronB4_P1-1_pos NeuronB4_P1-1-pos gnd! R='R_sense'
************************************************************************

*=======================================================================
*Events (write) detection circuit for CPA A-B partition array 1-1, group pos
************************************************************************
E1_A-B_P1-1_pos event_in_A-B_P1-1_pos! gnd! vol='((V(comparator_A-B_P1-1_pos-1)-0)*2e6)' min=0 max=1.2
gi_A-B_P1-1_pos comparator_A-B_P1-1_pos comparator_A-B_P1-1_pos-1 vcr pwl(1) enable_read_pulse gnd! 0.1,100Meg 0.9,1
Raux_B-A_P1-1_pos comparator_A-B_P1-1_pos-1 gnd! R=10k
Rshunt_B-A_P1-1_pos comparator_A-B_P1-1_pos gnd! R='R_shunt'

Xbuf_event_A-B_P1-1_pos event_in_A-B_P1-1_pos! event_in_m_A-B_P1-1_pos! bufx4tf
Xinv_event_A-B_P1-1_pos event_in_m_A-B_P1-1_pos! event_out_A-B_P1-1_pos! invx4tf
XinvReset resetDFFs sr_rw_sync invx4tf
Xevent_det_A-B_P1-1_pos clk_event_A-B gnd! EVENT_A-B_P1-1_pos! EVENTn_A-B_P1-1_pos! sr_rw_sync event_out_A-B_P1-1_pos! dffsrx4tf
Xbuf_enable_write_A-B_P1-1_pos EVENTn_A-B_P1-1_pos! enable_write_A-B_P1-1_pos bufx4tf
************************************************************************
*=======================================================================
*Layers (A-B) interconnections (CPA): Partition 2-1, pos polarity
************************************************************************
XMEMD_network_A-B_P2-1_pos AB1_PAD_1_P2-1_pos AB2_PAD_1_P2-1_pos BA1_PAD_1_P2-1_pos BA2_PAD_1_P2-1_pos BA3_PAD_1_P2-1_pos BA4_PAD_1_P2-1_pos MEMD_network_2_by_4
```

```
H0_r1_c1=0.443228 H0_r1_c2=0.443228 H0_r1_c3=0.443228 H0_r1_c4=0.443228
H0_r2_c1=0.443228 H0_r2_c2=0.443228 H0_r2_c3=0.443228 H0_r2_c4=0.443228

*=========================================================================
*Drivers for the input neurons of intercon. layer A-B (Neural layer A, CPA: 2-1-pos)
*************************************************************************
Xselector_RW_AB1-1-P2-1_pos AB1_PAD_1_P2-1_pos RW_A-B write_signal_AB1_1_P2-1_pos NeuronA1_P2-1 selector
Xselector_RW_AB1-2-P2-1_pos write_signal_AB1_1_P2-1_pos VcontAB1-1_out_P2-1_pos Vpulsedsignal RHZ_AB1_P2-1_pos selector
VHZ_AB1_P2-1_pos RHZ_AB1_P2-1_pos gnd! dc=0.000000
XandAB1_P2-1_pos VcontA-B1-1 enable_write_A-B_P2-1_pos VcontAB1-1_out_P2-1_pos and2x4tf
VNeuronA1_P2 NeuronA1_P2-1 gnd! PWL(0 0 1.000000000000e-06 0 1.001000000000e-06 0.000000 1.100100000000e-05 0.000000 1.100200000000e-05 0.000000 2.100200000000e-05 0.000000 2.100300000000e-05 0.300000 3.100300000000e-05 0.300000 3.100400000000e-05 0.000000 4.100400000000e-05 0.000000 4.100500000000e-05 0.000000 5.200420000000e-05 0.000000)
*************************************************************************
Xselector_RW_AB2-1-P2-1_pos AB2_PAD_1_P2-1_pos RW_A-B write_signal_AB2_1_P2-1_pos NeuronA2_P2-1 selector
Xselector_RW_AB2-2-P2-1_pos write_signal_AB2_1_P2-1_pos VcontAB2-1_out_P2-1_pos Vpulsedsignal RHZ_AB2_P2-1_pos selector
VHZ_AB2_P2-1_pos RHZ_AB2_P2-1_pos gnd! dc=0.000000
XandAB2_P2-1_pos VcontA-B2-1 enable_write_A-B_P2-1_pos VcontAB2-1_out_P2-1_pos and2x4tf
VNeuronA2_P2 NeuronA2_P2-1 gnd! PWL(0 0 1.000000000000e-06 0 1.001000000000e-06 0.000000 1.100100000000e-05 0.000000 1.100200000000e-05 0.000000 2.100200000000e-05 0.000000 2.100300000000e-05 0.000000 3.100300000000e-05 0.000000 3.100400000000e-05 0.300000 4.100400000000e-05 0.300000 4.100500000000e-05 0.300000 5.200420000000e-05 0.300000)
*************************************************************************

*=========================================================================
*Drivers for the output neurons of intercon. layer A-B (Neural layer B, CPA: 2-1-pos)
*************************************************************************
Xselector_RW_BA1-1-P2-1_pos BA1_PAD_1_P2-1_pos RW_A-B write_signal_BA1_1_P2-1_pos NeuronB1_P2-1-pos selector
Xselector_RW_BA1-2-P2-1_pos write_signal_BA1_1_P2-1_pos VcontBA1-1_out_P2-1_pos comparator_A-B_P2-1_pos RHZ_BA1_P2-1_pos selector
VHZ_BA1_P2-1_pos RHZ_BA1_P2-1_pos gnd! dc=0.650000
XandBA1_P2-1_pos VcontB-A1-1 enable_write_A-B_P2-1_pos VcontBA1-1_out_P2-1_pos and2x4tf
RNeuronB1_P2-1_pos NeuronB1_P2-1-pos gnd! R='R_sense'
*************************************************************************
Xselector_RW_BA2-1-P2-1_pos BA2_PAD_1_P2-1_pos RW_A-B write_signal_BA2_1_P2-1_pos NeuronB2_P2-1-pos selector
Xselector_RW_BA2-2-P2-1_pos write_signal_BA2_1_P2-1_pos VcontBA2-1_out_P2-1_pos comparator_A-B_P2-1_pos RHZ_BA2_P2-1_pos selector
VHZ_BA2_P2-1_pos RHZ_BA2_P2-1_pos gnd! dc=0.650000
XandBA2_P2-1_pos VcontB-A2-1 enable_write_A-B_P2-1_pos VcontBA2-1_out_P2-1_pos and2x4tf
RNeuronB2_P2-1_pos NeuronB2_P2-1-pos gnd! R='R_sense'
*************************************************************************
Xselector_RW_BA3-1-P2-1_pos BA3_PAD_1_P2-1_pos RW_A-B write_signal_BA3_1_P2-1_pos NeuronB3_P2-1-pos selector
Xselector_RW_BA3-2-P2-1_pos write_signal_BA3_1_P2-1_pos VcontBA3-1_out_P2-1_pos comparator_A-B_P2-1_pos RHZ_BA3_P2-1_pos selector
VHZ_BA3_P2-1_pos RHZ_BA3_P2-1_pos gnd! dc=0.650000
XandBA3_P2-1_pos VcontB-A3-1 enable_write_A-B_P2-1_pos VcontBA3-1_out_P2-1_pos and2x4tf
RNeuronB3_P2-1_pos NeuronB3_P2-1-pos gnd! R='R_sense'
*************************************************************************
Xselector_RW_BA4-1-P2-1_pos BA4_PAD_1_P2-1_pos RW_A-B write_signal_BA4_1_P2-1_pos NeuronB4_P2-1-pos selector
Xselector_RW_BA4-2-P2-1_pos write_signal_BA4_1_P2-1_pos VcontBA4-1_out_P2-1_pos comparator_A-B_P2-1_pos RHZ_BA4_P2-1_pos selector
VHZ_BA4_P2-1_pos RHZ_BA4_P2-1_pos gnd! dc=0.650000
XandBA4_P2-1_pos VcontB-A4-1 enable_write_A-B_P2-1_pos VcontBA4-1_out_P2-1_pos and2x4tf
RNeuronB4_P2-1_pos NeuronB4_P2-1-pos gnd! R='R_sense'
*************************************************************************

*=========================================================================
*Events (write) detection circuit for CPA A-B partition array 2-1, group pos
*************************************************************************
E1_A-B_P2-1_pos event_in_A-B_P2-1_pos! gnd! vol='((V(comparator_A-B_P2-1_pos-1)-0)*2e6)' min=0 max=1.2
gi_A-B_P2-1_pos comparator_A-B_P2-1_pos comparator_A-B_P2-1_pos-1 vcr pwl(1) enable_read_pulse gnd! 0.1,100Meg 0.9,1
Raux_B-A_P2-1_pos comparator_A-B_P2-1_pos-1 gnd! R=10k
Rshunt_B-A_P2-1_pos comparator_A-B_P2-1_pos gnd! R='R_shunt'

Xbuf_event_A-B_P2-1_pos event_in_A-B_P2-1_pos! event_in_m_A-B_P2-1_pos! bufx4tf
Xinv_event_A-B_P2-1_pos event_in_m_A-B_P2-1_pos! event_out_A-B_P2-1_pos! invx4tf
Xevent_det_A-B_P2-1_pos clk_event_A-B gnd! EVENT_A-B_P2-1_pos! EVENTn_A-B_P2-1_pos! sr_rw_sync event_out_A-B_P2-1_pos! dffsrx4tf
Xbuf_enable_write_A-B_P2-1_pos EVENTn_A-B_P2-1_pos! enable_write_A-B_P2-1_pos bufx4tf
```

```
*************************************************************************
*=======================================================================
*Layers (A-B) interconnections (CPA): Partition 1-1, neg polarity
*************************************************************************
XMEMD_network_A-B_P1-1_neg AB1_PAD_1_P1-1_neg AB2_PAD_1_P1-1_neg BA1_PAD_1_P1-1_neg BA2_PAD_1_P1-1_neg BA3_PAD_1_P1-1_neg BA4_PAD_1_P1-1_neg MEMD_network_2_by_4 H0_r1_c1=0.003252 H0_r1_c2=0.003252 H0_r1_c3=0.003252 H0_r1_c4=0.003252 H0_r2_c1=0.003252 H0_r2_c2=0.003252 H0_r2_c3=0.003252 H0_r2_c4=0.003252

*=======================================================================
*Drivers for the input neurons of intercon. layer A-B (Neural layer A, CPA: 1-1-neg)
*************************************************************************
Xselector_RW_AB1-1-P1-1_neg AB1_PAD_1_P1-1_neg RW_A-B write_signal_AB1_1_P1-1_neg NeuronA1_P1-1 selector
Xselector_RW_AB1-2-P1-1_neg write_signal_AB1_1_P1-1_neg VcontAB1-1_out_P1-1_neg Vpulsedsignal RHZ_AB1_P1-1_neg selector
VHZ_AB1_P1-1_neg RHZ_AB1_P1-1_neg gnd! dc=0.000000
XandAB1_P1-1_neg VcontA-B1-1 enable_write_A-B_P1-1_neg VcontAB1-1_out_P1-1_neg and2x4tf
*************************************************************************
Xselector_RW_AB2-1-P1-1_neg AB2_PAD_1_P1-1_neg RW_A-B write_signal_AB2_1_P1-1_neg NeuronA2_P1-1 selector
Xselector_RW_AB2-2-P1-1_neg write_signal_AB2_1_P1-1_neg VcontAB2-1_out_P1-1_neg Vpulsedsignal RHZ_AB2_P1-1_neg selector
VHZ_AB2_P1-1_neg RHZ_AB2_P1-1_neg gnd! dc=0.000000
XandAB2_P1-1_neg VcontA-B2-1 enable_write_A-B_P1-1_neg VcontAB2-1_out_P1-1_neg and2x4tf
*************************************************************************

*=======================================================================
*Drivers for the output neurons of intercon. layer A-B (Neural layer B, CPA: 1-1-neg)
*************************************************************************
Xselector_RW_BA1-1-P1-1_neg BA1_PAD_1_P1-1_neg RW_A-B write_signal_BA1_1_P1-1_neg NeuronB1_P1-1-neg selector
Xselector_RW_BA1-2-P1-1_neg write_signal_BA1_1_P1-1_neg VcontBA1-1_out_P1-1_neg comparator_A-B_P1-1_neg RHZ_BA1_P1-1_neg selector
VHZ_BA1_P1-1_neg RHZ_BA1_P1-1_neg gnd! dc=0.650000
XandBA1_P1-1_neg VcontB-A1-1 enable_write_A-B_P1-1_neg VcontBA1-1_out_P1-1_neg and2x4tf
RNeuronB1_P1-1_neg NeuronB1_P1-1-neg gnd! R='R_sense'
*************************************************************************
Xselector_RW_BA2-1-P1-1_neg BA2_PAD_1_P1-1_neg RW_A-B write_signal_BA2_1_P1-1_neg NeuronB2_P1-1-neg selector
Xselector_RW_BA2-2-P1-1_neg write_signal_BA2_1_P1-1_neg VcontBA2-1_out_P1-1_neg comparator_A-B_P1-1_neg RHZ_BA2_P1-1_neg selector
VHZ_BA2_P1-1_neg RHZ_BA2_P1-1_neg gnd! dc=0.650000
XandBA2_P1-1_neg VcontB-A2-1 enable_write_A-B_P1-1_neg VcontBA2-1_out_P1-1_neg and2x4tf
RNeuronB2_P1-1_neg NeuronB2_P1-1-neg gnd! R='R_sense'
*************************************************************************
Xselector_RW_BA3-1-P1-1_neg BA3_PAD_1_P1-1_neg RW_A-B write_signal_BA3_1_P1-1_neg NeuronB3_P1-1-neg selector
Xselector_RW_BA3-2-P1-1_neg write_signal_BA3_1_P1-1_neg VcontBA3-1_out_P1-1_neg comparator_A-B_P1-1_neg RHZ_BA3_P1-1_neg selector
VHZ_BA3_P1-1_neg RHZ_BA3_P1-1_neg gnd! dc=0.650000
XandBA3_P1-1_neg VcontB-A3-1 enable_write_A-B_P1-1_neg VcontBA3-1_out_P1-1_neg and2x4tf
RNeuronB3_P1-1_neg NeuronB3_P1-1-neg gnd! R='R_sense'
*************************************************************************
Xselector_RW_BA4-1-P1-1_neg BA4_PAD_1_P1-1_neg RW_A-B write_signal_BA4_1_P1-1_neg NeuronB4_P1-1-neg selector
Xselector_RW_BA4-2-P1-1_neg write_signal_BA4_1_P1-1_neg VcontBA4-1_out_P1-1_neg comparator_A-B_P1-1_neg RHZ_BA4_P1-1_neg selector
VHZ_BA4_P1-1_neg RHZ_BA4_P1-1_neg gnd! dc=0.650000
XandBA4_P1-1_neg VcontB-A4-1 enable_write_A-B_P1-1_neg VcontBA4-1_out_P1-1_neg and2x4tf
RNeuronB4_P1-1_neg NeuronB4_P1-1-neg gnd! R='R_sense'
*************************************************************************

*=======================================================================
*Events (write) detection circuit for CPA A-B partition array 1-1, group neg
*************************************************************************
E1_A-B_P1-1_neg event_in_A-B_P1-1_neg! gnd! vol='((V(comparator_A-B_P1-1_neg-1)-0)*2e6)' min=0 max=1.2
gi_A-B_P1-1_neg comparator_A-B_P1-1_neg comparator_A-B_P1-1_neg-1 vcr pwl(1) enable_read_pulse gnd! 0.1,100Meg 0.9,1
Raux_B-A_P1-1_neg comparator_A-B_P1-1_neg-1 gnd! R=10k
Rshunt_B-A_P1-1_neg comparator_A-B_P1-1_neg gnd! R='R_shunt'

Xbuf_event_A-B_P1-1_neg event_in_A-B_P1-1_neg! event_in_m_A-B_P1-1_neg! bufx4tf
Xinv_event_A-B_P1-1_neg event_in_m_A-B_P1-1_neg! event_out_A-B_P1-1_neg! invx4tf
Xevent_det_A-B_P1-1_neg clk_event_A-B gnd! EVENT_A-B_P1-1_neg! EVENTn_A-B_P1-1_neg! sr_rw_sync event_out_A-B_P1-1_neg! dffsrx4tf
Xbuf_enable_write_A-B_P1-1_neg EVENTn_A-B_P1-1_neg! enable_write_A-B_P1-1_neg bufx4tf
*************************************************************************
*=======================================================================
```

```
*Layers (A-B) interconnections (CPA): Partition 2-1, neg polarity
**************************************************************************
XMEMD_network_A-B_P2-1_neg AB1_PAD_1_P2-1_neg AB2_PAD_1_P2-1_neg BA1_PAD_1_P2-1_neg BA2_PAD_1_P2-1_neg BA3_PAD_1_P2-1_neg BA4_PAD_1_P2-1_neg MEMD_network_2_by_4 H0_r1_c1=0.003252 H0_r1_c2=0.003252 H0_r1_c3=0.003252 H0_r1_c4=0.003252 H0_r2_c1=0.003252 H0_r2_c2=0.003252 H0_r2_c3=0.003252 H0_r2_c4=0.003252

*=========================================================================
*Drivers for the input neurons of intercon. layer A-B (Neural layer A, CPA: 2-1-neg)
**************************************************************************
Xselector_RW_AB1-1-P2-1_neg AB1_PAD_1_P2-1_neg RW_A-B write_signal_AB1_1_P2-1_neg NeuronA1_P2-1 selector
Xselector_RW_AB1-2-P2-1_neg write_signal_AB1_1_P2-1_neg VcontAB1-1_out_P2-1_neg Vpulsedsignal RHZ_AB1_P2-1_neg selector
VHZ_AB1_P2-1_neg RHZ_AB1_P2-1_neg gnd! dc=0.000000
XandAB1_P2-1_neg VcontA-B1-1 enable_write_A-B_P2-1_neg VcontAB1-1_out_P2-1_neg and2x4tf
**************************************************************************
Xselector_RW_AB2-1-P2-1_neg AB2_PAD_1_P2-1_neg RW_A-B write_signal_AB2_1_P2-1_neg NeuronA2_P2-1 selector
Xselector_RW_AB2-2-P2-1_neg write_signal_AB2_1_P2-1_neg VcontAB2-1_out_P2-1_neg Vpulsedsignal RHZ_AB2_P2-1_neg selector
VHZ_AB2_P2-1_neg RHZ_AB2_P2-1_neg gnd! dc=0.000000
XandAB2_P2-1_neg VcontA-B2-1 enable_write_A-B_P2-1_neg VcontAB2-1_out_P2-1_neg and2x4tf
**************************************************************************

*=========================================================================
*Drivers for the output neurons of intercon. layer A-B (Neural layer B, CPA: 2-1-neg)
**************************************************************************
Xselector_RW_BA1-1-P2-1_neg BA1_PAD_1_P2-1_neg RW_A-B write_signal_BA1_1_P2-1_neg NeuronB1_P2-1-neg selector
Xselector_RW_BA1-2-P2-1_neg write_signal_BA1_1_P2-1_neg VcontBA1-1_out_P2-1_neg comparator_A-B_P2-1_neg RHZ_BA1_P2-1_neg selector
VHZ_BA1_P2-1_neg RHZ_BA1_P2-1_neg gnd! dc=0.650000
XandBA1_P2-1_neg VcontB-A1-1 enable_write_A-B_P2-1_neg VcontBA1-1_out_P2-1_neg and2x4tf
RNeuronB1_P2-1_neg NeuronB1_P2-1-neg gnd! R='R_sense'
**************************************************************************
Xselector_RW_BA2-1-P2-1_neg BA2_PAD_1_P2-1_neg RW_A-B write_signal_BA2_1_P2-1_neg NeuronB2_P2-1-neg selector
Xselector_RW_BA2-2-P2-1_neg write_signal_BA2_1_P2-1_neg VcontBA2-1_out_P2-1_neg comparator_A-B_P2-1_neg RHZ_BA2_P2-1_neg selector
VHZ_BA2_P2-1_neg RHZ_BA2_P2-1_neg gnd! dc=0.650000
XandBA2_P2-1_neg VcontB-A2-1 enable_write_A-B_P2-1_neg VcontBA2-1_out_P2-1_neg and2x4tf
RNeuronB2_P2-1_neg NeuronB2_P2-1-neg gnd! R='R_sense'
**************************************************************************
Xselector_RW_BA3-1-P2-1_neg BA3_PAD_1_P2-1_neg RW_A-B write_signal_BA3_1_P2-1_neg NeuronB3_P2-1-neg selector
Xselector_RW_BA3-2-P2-1_neg write_signal_BA3_1_P2-1_neg VcontBA3-1_out_P2-1_neg comparator_A-B_P2-1_neg RHZ_BA3_P2-1_neg selector
VHZ_BA3_P2-1_neg RHZ_BA3_P2-1_neg gnd! dc=0.650000
XandBA3_P2-1_neg VcontB-A3-1 enable_write_A-B_P2-1_neg VcontBA3-1_out_P2-1_neg and2x4tf
RNeuronB3_P2-1_neg NeuronB3_P2-1-neg gnd! R='R_sense'
**************************************************************************
Xselector_RW_BA4-1-P2-1_neg BA4_PAD_1_P2-1_neg RW_A-B write_signal_BA4_1_P2-1_neg NeuronB4_P2-1-neg selector
Xselector_RW_BA4-2-P2-1_neg write_signal_BA4_1_P2-1_neg VcontBA4-1_out_P2-1_neg comparator_A-B_P2-1_neg RHZ_BA4_P2-1_neg selector
VHZ_BA4_P2-1_neg RHZ_BA4_P2-1_neg gnd! dc=0.650000
XandBA4_P2-1_neg VcontB-A4-1 enable_write_A-B_P2-1_neg VcontBA4-1_out_P2-1_neg and2x4tf
RNeuronB4_P2-1_neg NeuronB4_P2-1-neg gnd! R='R_sense'
**************************************************************************

*=========================================================================
*Events (write) detection circuit for CPA A-B partition array 2-1, group neg
**************************************************************************
E1_A-B_P2-1_neg event_in_A-B_P2-1_neg! gnd! vol='((V(comparator_A-B_P2-1_neg-1)-0)*2e6)' min=0 max=1.2
gi_A-B_P2-1_neg comparator_A-B_P2-1_neg comparator_A-B_P2-1_neg-1 vcr pwl(1) enable_read_pulse gnd! 0.1,100Meg 0.9,1
Raux_B-A_P2-1_neg comparator_A-B_P2-1_neg-1 gnd! R=10k
Rshunt_B-A_P2-1_neg comparator_A-B_P2-1_neg gnd! R='R_shunt'

Xbuf_event_A-B_P2-1_neg event_in_A-B_P2-1_neg! event_in_m_A-B_P2-1_neg! bufx4tf
Xinv_event_A-B_P2-1_neg event_in_m_A-B_P2-1_neg! event_out_A-B_P2-1_neg! invx4tf
Xevent_det_A-B_P2-1_neg clk_event_A-B gnd! EVENT_A-B_P2-1_neg! EVENTn_A-B_P2-1_neg! sr_rw_sync event_out_A-B_P2-1_neg! dffsrx4tf
Xbuf_enable_write_A-B_P2-1_neg EVENTn_A-B_P2-1_neg! enable_write_A-B_P2-1_neg bufx4tf
**************************************************************************
*=========================================================================
*Hidden layer of neurons (layer B)
**************************************************************************
```

```
XNeuronB1_P1-1 NeuronB1_P1-1-pos NeuronB1_P2-1-pos NeuronB1_P1-1-neg NeuronB1_P2-1-neg NeuronB1_P1-1 gnd! neuron_2x2_in
RNeuronB1_P1-1 NeuronB1_P1-1 gnd! R=100Meg
XNeuronB2_P1-1 NeuronB2_P1-1-pos NeuronB2_P2-1-pos NeuronB2_P1-1-neg NeuronB2_P2-1-neg NeuronB2_P1-1 gnd! neuron_2x2_in
RNeuronB2_P1-1 NeuronB2_P1-1 gnd! R=100Meg
XNeuronB3_P1-1 NeuronB3_P1-1-pos NeuronB3_P2-1-pos NeuronB3_P1-1-neg NeuronB3_P2-1-neg NeuronB3_P1-1 gnd! neuron_2x2_in
RNeuronB3_P1-1 NeuronB3_P1-1 gnd! R=100Meg
XNeuronB4_P1-1 NeuronB4_P1-1-pos NeuronB4_P2-1-pos NeuronB4_P1-1-neg NeuronB4_P2-1-neg NeuronB4_P1-1 gnd! neuron_2x2_in
RNeuronB4_P1-1 NeuronB4_P1-1 gnd! R=100Meg

************************************************************************
XdecoderA-B VcontA-B1-1 VcontA-B2-1 eventsA_A-B WR_user reset_count_deco_A-B decoder_2_output
Xcounter_A-B eventsA_A-B write_ok_pulse!_A-B reset_count_deco_A-B counter_2_pulses
Xinv_wr_pulse_A-B write_ok_pulse!_A-B write_ok_pulse_n!_A-B invx4tf
X_WR_OK_AB sys_clk gnd! write_ok!_A-B write_okn!_A-B resetDFFsn write_ok_pulse_n!_A-B dffsrx4tf
XdecoderB-A VcontB-A1-1 VcontB-A2-1 VcontB-A3-1 VcontB-A4-1 EVENT!_A-B WR_user reset_count_deco_A-B decoder_4_output
XcounterB EVENT!_A-B eventsA_A-B reset_count_deco_A-B counter_4_pulses

XorAB4 resetDFFs RWn_A-B reset_count_deco_A-B or2x4tf

Xevent_sync_A-B_pos event_A-B_P1-1_pos! event_A-B_P2-1_pos! EVENT_A-B_pos! and2x4tf
Xevent_sync_A-B_neg event_A-B_P1-1_neg! event_A-B_P2-1_neg! EVENT_A-B_neg! and2x4tf
Xclk_sync_A-B EVENT_A-B_pos! EVENT_A-B_neg! clk_event_n_A-B EVENT_S_A-B! and3x4tf
Xevent_sync_A-B EVENT_A-B_pos! EVENT_A-B_neg! EVENT!_A-B and2x4tf
Xclk_sync_DFF_A-B sys_clk EVENT_S_A-B! clk_event_A-B clk_event_n_A-B sr_rw_sync vdd! dffsrx4tf

*Read-write circuit synchronizer
************************************************************************
X_WR_control_A-B write_ok!_A-B gnd! RW_A-B RWn_A-B resetDFFsn wr_user_n dffsrx4tf
Xlayer_sync write_ok!_A-B write_ok! sys_clk vdd! resetDFFsn sync_W_OK_1_layers



*Read-write signals
************************************************************************
Xinv_rw_reset resetDFFs resetDFFsn invx4tf
Xinv_rw_user wr_user wr_user_n invx4tf

*logic gates power suply
************************************************************************
VVDD vdd! gnd! dc=1.2
VVSS vss! gnd! dc=0

*reser of DFFs
************************************************************************
VresetDFFs resetDFFs gnd! dc=0 pulse ( 0 1.2 1u 10n 10n 1u 5.200420000000e-05 )

*System clock
************************************************************************
Vclk sys_clk gnd! dc=0

*Pulsed signal for memristor programming
************************************************************************
Vsetting Vpulsedsignal gnd! dc=0
Vsetting_en enable_read_pulse gnd! dc=0

*Read-Write signal
************************************************************************
V_WR WR_user gnd! dc=0

*######################################################################

*######################  .LIB / .INC statements  ##########################

*######################################################################

*#########################  .PARAM statements  ############################

.PARAM Rs=10.000000
.PARAM Rs_PAD=10.000000
.PARAM n_extra=1
.PARAM Rcs=1.000000
.PARAM R_shunt=10.000000
```

```
.PARAM HZ=100Meg
.PARAM R_sense=1000.000000
.PARAM R_in_TIA=1.000000
*##########################################################################

*#########################  .SIM statements  ############################

.tran 1.000000000000e-06 5.200420000000e-05 0

*##########################################################################

*#########################  .PRINT statements  ############################

.PROBE TRAN V(xmemd_network_A-B_P1-1_pos.xmemdr1-1c1-1.h)
.PROBE TRAN I(xmemd_network_A-B_P1-1_pos.Rcs_r1-1c1-1_1)
.PROBE TRAN V(xmemd_network_A-B_P1-1_pos.r1-1_p)
.PROBE TRAN V(xmemd_network_A-B_P1-1_pos.c1-1_n)
.PROBE TRAN V(xmemd_network_A-B_P1-1_pos.xmemdr1-2c2-1.h)
.PROBE TRAN I(xmemd_network_A-B_P1-1_pos.Rcs_r1-2c2-1_1)
.PROBE TRAN V(xmemd_network_A-B_P1-1_pos.r1-2_p)
.PROBE TRAN V(xmemd_network_A-B_P1-1_pos.c2-1_n)
.PROBE TRAN V(xmemd_network_A-B_P1-1_pos.xmemdr1-3c3-1.h)
.PROBE TRAN I(xmemd_network_A-B_P1-1_pos.Rcs_r1-3c3-1_1)
.PROBE TRAN V(xmemd_network_A-B_P1-1_pos.r1-3_p)
.PROBE TRAN V(xmemd_network_A-B_P1-1_pos.c3-1_n)
.PROBE TRAN V(xmemd_network_A-B_P1-1_pos.xmemdr1-4c4-1.h)
.PROBE TRAN I(xmemd_network_A-B_P1-1_pos.Rcs_r1-4c4-1_1)
.PROBE TRAN V(xmemd_network_A-B_P1-1_pos.r1-4_p)
.PROBE TRAN V(xmemd_network_A-B_P1-1_pos.c4-1_n)
.PROBE TRAN V(xmemd_network_A-B_P1-1_pos.xmemdr2-1c1-2.h)
.PROBE TRAN I(xmemd_network_A-B_P1-1_pos.Rcs_r2-1c1-2_1)
.PROBE TRAN V(xmemd_network_A-B_P1-1_pos.r2-1_p)
.PROBE TRAN V(xmemd_network_A-B_P1-1_pos.c1-2_n)
.PROBE TRAN V(xmemd_network_A-B_P1-1_pos.xmemdr2-2c2-2.h)
.PROBE TRAN I(xmemd_network_A-B_P1-1_pos.Rcs_r2-2c2-2_1)
.PROBE TRAN V(xmemd_network_A-B_P1-1_pos.r2-2_p)
.PROBE TRAN V(xmemd_network_A-B_P1-1_pos.c2-2_n)
.PROBE TRAN V(xmemd_network_A-B_P1-1_pos.xmemdr2-3c3-2.h)
.PROBE TRAN I(xmemd_network_A-B_P1-1_pos.Rcs_r2-3c3-2_1)
.PROBE TRAN V(xmemd_network_A-B_P1-1_pos.r2-3_p)
.PROBE TRAN V(xmemd_network_A-B_P1-1_pos.c3-2_n)
.PROBE TRAN V(xmemd_network_A-B_P1-1_pos.xmemdr2-4c4-2.h)
.PROBE TRAN I(xmemd_network_A-B_P1-1_pos.Rcs_r2-4c4-2_1)
.PROBE TRAN V(xmemd_network_A-B_P1-1_pos.r2-4_p)
.PROBE TRAN V(xmemd_network_A-B_P1-1_pos.c4-2_n)
.PROBE TRAN i__RneuronB1_P1-1_pos__=PAR('1*ISUB(Xselector_RW_BA1-1-P1-1_pos.Neuron)')

.PROBE TRAN i__RneuronB2_P1-1_pos__=PAR('1*ISUB(Xselector_RW_BA2-1-P1-1_pos.Neuron)')

.PROBE TRAN i__RneuronB3_P1-1_pos__=PAR('1*ISUB(Xselector_RW_BA3-1-P1-1_pos.Neuron)')

.PROBE TRAN i__RneuronB4_P1-1_pos__=PAR('1*ISUB(Xselector_RW_BA4-1-P1-1_pos.Neuron)')

.PROBE TRAN V(enable_write_A-B_P1-1_pos)
.PROBE TRAN V(comparator_A-B_P1-1_pos)
.PROBE TRAN V(comparator_A-B_P1-1_pos-1)
.PROBE TRAN V(EVENT_A-B_P1-1_pos!)
.PROBE TRAN V(xmemd_network_A-B_P2-1_pos.xmemdr1-1c1-1.h)
.PROBE TRAN I(xmemd_network_A-B_P2-1_pos.Rcs_r1-1c1-1_1)
.PROBE TRAN V(xmemd_network_A-B_P2-1_pos.r1-1_p)
.PROBE TRAN V(xmemd_network_A-B_P2-1_pos.c1-1_n)
.PROBE TRAN V(xmemd_network_A-B_P2-1_pos.xmemdr1-2c2-1.h)
.PROBE TRAN I(xmemd_network_A-B_P2-1_pos.Rcs_r1-2c2-1_1)
.PROBE TRAN V(xmemd_network_A-B_P2-1_pos.r1-2_p)
.PROBE TRAN V(xmemd_network_A-B_P2-1_pos.c2-1_n)
.PROBE TRAN V(xmemd_network_A-B_P2-1_pos.xmemdr1-3c3-1.h)
.PROBE TRAN I(xmemd_network_A-B_P2-1_pos.Rcs_r1-3c3-1_1)
.PROBE TRAN V(xmemd_network_A-B_P2-1_pos.r1-3_p)
.PROBE TRAN V(xmemd_network_A-B_P2-1_pos.c3-1_n)
.PROBE TRAN V(xmemd_network_A-B_P2-1_pos.xmemdr1-4c4-1.h)
.PROBE TRAN I(xmemd_network_A-B_P2-1_pos.Rcs_r1-4c4-1_1)
.PROBE TRAN V(xmemd_network_A-B_P2-1_pos.r1-4_p)
.PROBE TRAN V(xmemd_network_A-B_P2-1_pos.c4-1_n)
.PROBE TRAN V(xmemd_network_A-B_P2-1_pos.xmemdr2-1c1-2.h)
.PROBE TRAN I(xmemd_network_A-B_P2-1_pos.Rcs_r2-1c1-2_1)
.PROBE TRAN V(xmemd_network_A-B_P2-1_pos.r2-1_p)
.PROBE TRAN V(xmemd_network_A-B_P2-1_pos.c1-2_n)
.PROBE TRAN V(xmemd_network_A-B_P2-1_pos.xmemdr2-2c2-2.h)
.PROBE TRAN I(xmemd_network_A-B_P2-1_pos.Rcs_r2-2c2-2_1)
.PROBE TRAN V(xmemd_network_A-B_P2-1_pos.r2-2_p)
```

```
.PROBE TRAN V(xmemd_network_A-B_P2-1_pos.c2-2_n)
.PROBE TRAN V(xmemd_network_A-B_P2-1_pos.xmemdr2-3c3-2.h)
.PROBE TRAN I(xmemd_network_A-B_P2-1_pos.Rcs_r2-3c3-2_1)
.PROBE TRAN V(xmemd_network_A-B_P2-1_pos.r2-3_p)
.PROBE TRAN V(xmemd_network_A-B_P2-1_pos.c3-2_n)
.PROBE TRAN V(xmemd_network_A-B_P2-1_pos.xmemdr2-4c4-2.h)
.PROBE TRAN I(xmemd_network_A-B_P2-1_pos.Rcs_r2-4c4-2_1)
.PROBE TRAN V(xmemd_network_A-B_P2-1_pos.r2-4_p)
.PROBE TRAN V(xmemd_network_A-B_P2-1_pos.c4-2_n)
.PROBE TRAN i__RneuronB1_P2-1_pos__=PAR('1*ISUB(Xselector_RW_BA1-1-P2-1_pos.Neuron)')
.PROBE TRAN i__RneuronB2_P2-1_pos__=PAR('1*ISUB(Xselector_RW_BA2-1-P2-1_pos.Neuron)')
.PROBE TRAN i__RneuronB3_P2-1_pos__=PAR('1*ISUB(Xselector_RW_BA3-1-P2-1_pos.Neuron)')
.PROBE TRAN i__RneuronB4_P2-1_pos__=PAR('1*ISUB(Xselector_RW_BA4-1-P2-1_pos.Neuron)')
.PROBE TRAN V(enable_write_A-B_P2-1_pos)
.PROBE TRAN V(comparator_A-B_P2-1_pos)
.PROBE TRAN V(comparator_A-B_P2-1_pos-1)
.PROBE TRAN V(EVENT_A-B_P2-1_pos!)
.PROBE TRAN V(EVENT_A-B_pos!)
.PROBE TRAN V(xmemd_network_A-B_P1-1_neg.xmemdr1-1c1-1.h)
.PROBE TRAN I(xmemd_network_A-B_P1-1_neg.Rcs_r1-1c1-1_1)
.PROBE TRAN V(xmemd_network_A-B_P1-1_neg.r1-1_p)
.PROBE TRAN V(xmemd_network_A-B_P1-1_neg.c1-1_n)
.PROBE TRAN V(xmemd_network_A-B_P1-1_neg.xmemdr1-2c2-1.h)
.PROBE TRAN I(xmemd_network_A-B_P1-1_neg.Rcs_r1-2c2-1_1)
.PROBE TRAN V(xmemd_network_A-B_P1-1_neg.r1-2_p)
.PROBE TRAN V(xmemd_network_A-B_P1-1_neg.c2-1_n)
.PROBE TRAN V(xmemd_network_A-B_P1-1_neg.xmemdr1-3c3-1.h)
.PROBE TRAN I(xmemd_network_A-B_P1-1_neg.Rcs_r1-3c3-1_1)
.PROBE TRAN V(xmemd_network_A-B_P1-1_neg.r1-3_p)
.PROBE TRAN V(xmemd_network_A-B_P1-1_neg.c3-1_n)
.PROBE TRAN V(xmemd_network_A-B_P1-1_neg.xmemdr1-4c4-1.h)
.PROBE TRAN I(xmemd_network_A-B_P1-1_neg.Rcs_r1-4c4-1_1)
.PROBE TRAN V(xmemd_network_A-B_P1-1_neg.r1-4_p)
.PROBE TRAN V(xmemd_network_A-B_P1-1_neg.c4-1_n)
.PROBE TRAN V(xmemd_network_A-B_P1-1_neg.xmemdr2-1c1-2.h)
.PROBE TRAN I(xmemd_network_A-B_P1-1_neg.Rcs_r2-1c1-2_1)
.PROBE TRAN V(xmemd_network_A-B_P1-1_neg.r2-1_p)
.PROBE TRAN V(xmemd_network_A-B_P1-1_neg.c1-2_n)
.PROBE TRAN V(xmemd_network_A-B_P1-1_neg.xmemdr2-2c2-2.h)
.PROBE TRAN I(xmemd_network_A-B_P1-1_neg.Rcs_r2-2c2-2_1)
.PROBE TRAN V(xmemd_network_A-B_P1-1_neg.r2-2_p)
.PROBE TRAN V(xmemd_network_A-B_P1-1_neg.c2-2_n)
.PROBE TRAN V(xmemd_network_A-B_P1-1_neg.xmemdr2-3c3-2.h)
.PROBE TRAN I(xmemd_network_A-B_P1-1_neg.Rcs_r2-3c3-2_1)
.PROBE TRAN V(xmemd_network_A-B_P1-1_neg.r2-3_p)
.PROBE TRAN V(xmemd_network_A-B_P1-1_neg.c3-2_n)
.PROBE TRAN V(xmemd_network_A-B_P1-1_neg.xmemdr2-4c4-2.h)
.PROBE TRAN I(xmemd_network_A-B_P1-1_neg.Rcs_r2-4c4-2_1)
.PROBE TRAN V(xmemd_network_A-B_P1-1_neg.r2-4_p)
.PROBE TRAN V(xmemd_network_A-B_P1-1_neg.c4-2_n)
.PROBE TRAN i__RneuronB1_P1-1_neg__=PAR('1*ISUB(Xselector_RW_BA1-1-P1-1_neg.Neuron)')
.PROBE TRAN i__RneuronB2_P1-1_neg__=PAR('1*ISUB(Xselector_RW_BA2-1-P1-1_neg.Neuron)')
.PROBE TRAN i__RneuronB3_P1-1_neg__=PAR('1*ISUB(Xselector_RW_BA3-1-P1-1_neg.Neuron)')
.PROBE TRAN i__RneuronB4_P1-1_neg__=PAR('1*ISUB(Xselector_RW_BA4-1-P1-1_neg.Neuron)')
.PROBE TRAN V(enable_write_A-B_P1-1_neg)
.PROBE TRAN V(comparator_A-B_P1-1_neg)
.PROBE TRAN V(comparator_A-B_P1-1_neg-1)
.PROBE TRAN V(EVENT_A-B_P1-1_neg!)
.PROBE TRAN V(xmemd_network_A-B_P2-1_neg.xmemdr1-1c1-1.h)
.PROBE TRAN I(xmemd_network_A-B_P2-1_neg.Rcs_r1-1c1-1_1)
.PROBE TRAN V(xmemd_network_A-B_P2-1_neg.r1-1_p)
.PROBE TRAN V(xmemd_network_A-B_P2-1_neg.c1-1_n)
.PROBE TRAN V(xmemd_network_A-B_P2-1_neg.xmemdr1-2c2-1.h)
.PROBE TRAN I(xmemd_network_A-B_P2-1_neg.Rcs_r1-2c2-1_1)
.PROBE TRAN V(xmemd_network_A-B_P2-1_neg.r1-2_p)
.PROBE TRAN V(xmemd_network_A-B_P2-1_neg.c2-1_n)
.PROBE TRAN V(xmemd_network_A-B_P2-1_neg.xmemdr1-3c3-1.h)
.PROBE TRAN I(xmemd_network_A-B_P2-1_neg.Rcs_r1-3c3-1_1)
.PROBE TRAN V(xmemd_network_A-B_P2-1_neg.r1-3_p)
.PROBE TRAN V(xmemd_network_A-B_P2-1_neg.c3-1_n)
.PROBE TRAN V(xmemd_network_A-B_P2-1_neg.xmemdr1-4c4-1.h)
.PROBE TRAN I(xmemd_network_A-B_P2-1_neg.Rcs_r1-4c4-1_1)
```

```
.PROBE TRAN V(xmemd_network_A-B_P2-1_neg.r1-4_p)
.PROBE TRAN V(xmemd_network_A-B_P2-1_neg.c4-1_n)
.PROBE TRAN V(xmemd_network_A-B_P2-1_neg.xmemdr2-1c1-2.h)
.PROBE TRAN I(xmemd_network_A-B_P2-1_neg.Rcs_r2-1c1-2_1)
.PROBE TRAN V(xmemd_network_A-B_P2-1_neg.r2-1_p)
.PROBE TRAN V(xmemd_network_A-B_P2-1_neg.c1-2_n)
.PROBE TRAN V(xmemd_network_A-B_P2-1_neg.xmemdr2-2c2-2.h)
.PROBE TRAN I(xmemd_network_A-B_P2-1_neg.Rcs_r2-2c2-2_1)
.PROBE TRAN V(xmemd_network_A-B_P2-1_neg.r2-2_p)
.PROBE TRAN V(xmemd_network_A-B_P2-1_neg.c2-2_n)
.PROBE TRAN V(xmemd_network_A-B_P2-1_neg.xmemdr2-3c3-2.h)
.PROBE TRAN I(xmemd_network_A-B_P2-1_neg.Rcs_r2-3c3-2_1)
.PROBE TRAN V(xmemd_network_A-B_P2-1_neg.r2-3_p)
.PROBE TRAN V(xmemd_network_A-B_P2-1_neg.c3-2_n)
.PROBE TRAN V(xmemd_network_A-B_P2-1_neg.xmemdr2-4c4-2.h)
.PROBE TRAN I(xmemd_network_A-B_P2-1_neg.Rcs_r2-4c4-2_1)
.PROBE TRAN V(xmemd_network_A-B_P2-1_neg.r2-4_p)
.PROBE TRAN V(xmemd_network_A-B_P2-1_neg.c4-2_n)
.PROBE TRAN i__RneuronB1_P2-1_neg__=PAR('1*ISUB(Xselector_RW_BA1-1-P2-1_neg.Neuron)')

.PROBE TRAN i__RneuronB2_P2-1_neg__=PAR('1*ISUB(Xselector_RW_BA2-1-P2-1_neg.Neuron)')

.PROBE TRAN i__RneuronB3_P2-1_neg__=PAR('1*ISUB(Xselector_RW_BA3-1-P2-1_neg.Neuron)')

.PROBE TRAN i__RneuronB4_P2-1_neg__=PAR('1*ISUB(Xselector_RW_BA4-1-P2-1_neg.Neuron)')

.PROBE TRAN V(NeuronB1_P1-1)

.PROBE TRAN V(NeuronB2_P1-1)

.PROBE TRAN V(NeuronB3_P1-1)

.PROBE TRAN V(NeuronB4_P1-1)

.PROBE TRAN V(enable_write_A-B_P2-1_neg)
.PROBE TRAN V(comparator_A-B_P2-1_neg)
.PROBE TRAN V(comparator_A-B_P2-1_neg-1)
.PROBE TRAN V(EVENT_A-B_P2-1_neg!)
.PROBE TRAN V(EVENT_A-B_neg!)

.PROBE TRAN V(write_ok!)
.PROBE TRAN V(sys_clk)
.PROBE TRAN V(RW)
.PROBE TRAN V(eventsA)
.PROBE TRAN V(Vpulsedsignal)
.PROBE TRAN V(enable_read_pulse)
.MEAS TRAN V(xmemd_network_A-B_P1-1_pos.xmemdr1-1c1-1.h) FIND V(xmemd_network_A-B_P1-1_pos.xmemdr1-1c1-1.h) AT=5.200420000000e-05
.MEAS TRAN V(xmemd_network_A-B_P1-1_pos.xmemdr1-2c2-1.h) FIND V(xmemd_network_A-B_P1-1_pos.xmemdr1-2c2-1.h) AT=5.200420000000e-05
.MEAS TRAN V(xmemd_network_A-B_P1-1_pos.xmemdr1-3c3-1.h) FIND V(xmemd_network_A-B_P1-1_pos.xmemdr1-3c3-1.h) AT=5.200420000000e-05
.MEAS TRAN V(xmemd_network_A-B_P1-1_pos.xmemdr1-4c4-1.h) FIND V(xmemd_network_A-B_P1-1_pos.xmemdr1-4c4-1.h) AT=5.200420000000e-05
.MEAS TRAN V(xmemd_network_A-B_P1-1_pos.xmemdr2-1c1-2.h) FIND V(xmemd_network_A-B_P1-1_pos.xmemdr2-1c1-2.h) AT=5.200420000000e-05
.MEAS TRAN V(xmemd_network_A-B_P1-1_pos.xmemdr2-2c2-2.h) FIND V(xmemd_network_A-B_P1-1_pos.xmemdr2-2c2-2.h) AT=5.200420000000e-05
.MEAS TRAN V(xmemd_network_A-B_P1-1_pos.xmemdr2-3c3-2.h) FIND V(xmemd_network_A-B_P1-1_pos.xmemdr2-3c3-2.h) AT=5.200420000000e-05
.MEAS TRAN V(xmemd_network_A-B_P1-1_pos.xmemdr2-4c4-2.h) FIND V(xmemd_network_A-B_P1-1_pos.xmemdr2-4c4-2.h) AT=5.200420000000e-05
.MEAS TRAN V(xmemd_network_A-B_P2-1_pos.xmemdr1-1c1-1.h) FIND V(xmemd_network_A-B_P2-1_pos.xmemdr1-1c1-1.h) AT=5.200420000000e-05
.MEAS TRAN V(xmemd_network_A-B_P2-1_pos.xmemdr1-2c2-1.h) FIND V(xmemd_network_A-B_P2-1_pos.xmemdr1-2c2-1.h) AT=5.200420000000e-05
.MEAS TRAN V(xmemd_network_A-B_P2-1_pos.xmemdr1-3c3-1.h) FIND V(xmemd_network_A-B_P2-1_pos.xmemdr1-3c3-1.h) AT=5.200420000000e-05
.MEAS TRAN V(xmemd_network_A-B_P2-1_pos.xmemdr1-4c4-1.h) FIND V(xmemd_network_A-B_P2-1_pos.xmemdr1-4c4-1.h) AT=5.200420000000e-05
.MEAS TRAN V(xmemd_network_A-B_P2-1_pos.xmemdr2-1c1-2.h) FIND V(xmemd_network_A-B_P2-1_pos.xmemdr2-1c1-2.h) AT=5.200420000000e-05
.MEAS TRAN V(xmemd_network_A-B_P2-1_pos.xmemdr2-2c2-2.h) FIND V(xmemd_network_A-B_P2-1_pos.xmemdr2-2c2-2.h) AT=5.200420000000e-05
.MEAS TRAN V(xmemd_network_A-B_P2-1_pos.xmemdr2-3c3-2.h) FIND V(xmemd_network_A-B_P2-1_pos.xmemdr2-3c3-2.h) AT=5.200420000000e-05
.MEAS TRAN V(xmemd_network_A-B_P2-1_pos.xmemdr2-4c4-2.h) FIND V(xmemd_network_A-B_P2-1_pos.xmemdr2-4c4-2.h) AT=5.200420000000e-05
.MEAS TRAN V(xmemd_network_A-B_P1-1_neg.xmemdr1-1c1-1.h) FIND V(xmemd_network_A-B_P1-1_neg.xmemdr1-1c1-1.h) AT=5.200420000000e-05
```

```
.MEAS TRAN V(xmemd_network_A-B_P1-1_neg.xmemdr1-2c2-1.h) FIND V(xmemd_network_A-B_P1-1_neg.xmemdr1-2c2-1.h) AT=5.200420000000e-05
.MEAS TRAN V(xmemd_network_A-B_P1-1_neg.xmemdr1-3c3-1.h) FIND V(xmemd_network_A-B_P1-1_neg.xmemdr1-3c3-1.h) AT=5.200420000000e-05
.MEAS TRAN V(xmemd_network_A-B_P1-1_neg.xmemdr1-4c4-1.h) FIND V(xmemd_network_A-B_P1-1_neg.xmemdr1-4c4-1.h) AT=5.200420000000e-05
.MEAS TRAN V(xmemd_network_A-B_P1-1_neg.xmemdr2-1c1-2.h) FIND V(xmemd_network_A-B_P1-1_neg.xmemdr2-1c1-2.h) AT=5.200420000000e-05
.MEAS TRAN V(xmemd_network_A-B_P1-1_neg.xmemdr2-2c2-2.h) FIND V(xmemd_network_A-B_P1-1_neg.xmemdr2-2c2-2.h) AT=5.200420000000e-05
.MEAS TRAN V(xmemd_network_A-B_P1-1_neg.xmemdr2-3c3-2.h) FIND V(xmemd_network_A-B_P1-1_neg.xmemdr2-3c3-2.h) AT=5.200420000000e-05
.MEAS TRAN V(xmemd_network_A-B_P1-1_neg.xmemdr2-4c4-2.h) FIND V(xmemd_network_A-B_P1-1_neg.xmemdr2-4c4-2.h) AT=5.200420000000e-05
.MEAS TRAN V(xmemd_network_A-B_P2-1_neg.xmemdr1-1c1-1.h) FIND V(xmemd_network_A-B_P2-1_neg.xmemdr1-1c1-1.h) AT=5.200420000000e-05
.MEAS TRAN V(xmemd_network_A-B_P2-1_neg.xmemdr1-2c2-1.h) FIND V(xmemd_network_A-B_P2-1_neg.xmemdr1-2c2-1.h) AT=5.200420000000e-05
.MEAS TRAN V(xmemd_network_A-B_P2-1_neg.xmemdr1-3c3-1.h) FIND V(xmemd_network_A-B_P2-1_neg.xmemdr1-3c3-1.h) AT=5.200420000000e-05
.MEAS TRAN V(xmemd_network_A-B_P2-1_neg.xmemdr1-4c4-1.h) FIND V(xmemd_network_A-B_P2-1_neg.xmemdr1-4c4-1.h) AT=5.200420000000e-05
.MEAS TRAN V(xmemd_network_A-B_P2-1_neg.xmemdr2-1c1-2.h) FIND V(xmemd_network_A-B_P2-1_neg.xmemdr2-1c1-2.h) AT=5.200420000000e-05
.MEAS TRAN V(xmemd_network_A-B_P2-1_neg.xmemdr2-2c2-2.h) FIND V(xmemd_network_A-B_P2-1_neg.xmemdr2-2c2-2.h) AT=5.200420000000e-05
.MEAS TRAN V(xmemd_network_A-B_P2-1_neg.xmemdr2-3c3-2.h) FIND V(xmemd_network_A-B_P2-1_neg.xmemdr2-3c3-2.h) AT=5.200420000000e-05
.MEAS TRAN V(xmemd_network_A-B_P2-1_neg.xmemdr2-4c4-2.h) FIND V(xmemd_network_A-B_P2-1_neg.xmemdr2-4c4-2.h) AT=5.200420000000e-05


*########################################################################

.end
```

Supplementary references

[1] I. Chakraborty *et al.*, "Resistive Crossbars as Approximate Hardware Building Blocks for Machine Learning: Opportunities and Challenges," *Proc. IEEE*, vol. 108, no. 12, pp. 2276–2310, 2020, doi: 10.1109/JPROC.2020.3003007.

[2] M. Le Gallo *et al.*, "Precision of bit slicing with in-memory computing based on analog phase-change memory crossbars," *Neuromorphic Comput. Eng.*, vol. 2, no. 1, p. 014009, Mar. 2022, doi: 10.1088/2634-4386/ac4fb7.

[3] J. M. Hung *et al.*, "A four-megabit compute-in-memory macro with eight-bit precision based on CMOS and resistive random-access memory for AI edge devices," *Nat. Electron. 2021 412*, vol. 4, no. 12, pp. 921–930, Dec. 2021, doi: 10.1038/s41928-021-00676-9.

[4] C. X. Xue *et al.*, "A CMOS-integrated compute-in-memory macro based on resistive random-access memory for AI edge devices," *Nat. Electron.*, vol. 4, no. 1, pp. 81–90, 2021, doi: 10.1038/s41928-020-00505-5.

[5] J.-M. Hung *et al.*, "An 8-Mb DC-Current-Free Binary-to-8b Precision ReRAM Nonvolatile Computing-in-Memory Macro using Time-Space-Readout with 1286.4-21.6TOPS/W for Edge-AI Devices," in *2022 IEEE International Solid- State Circuits Conference (ISSCC)*, Feb. 2022, pp. 1–3, doi: 10.1109/ISSCC42614.2022.9731715.

[6] W.-H. Huang *et al.*, "A Nonvolatile Al-Edge Processor with 4MB SLC-MLC Hybrid-Mode ReRAM Compute-in-Memory Macro and 51.4-251TOPS/W," *2023 IEEE Int. Solid- State Circuits Conf.*, pp. 15–17, Feb. 2023, doi: 10.1109/ISSCC42615.2023.10067610.

[7] Y.-C. Chiu *et al.*, "A 22nm 8Mb STT-MRAM Near-Memory-Computing Macro with 8b-Precision and 46.4-160.1TOPS/W for Edge-AI Devices," in *2023 IEEE International Solid-State Circuits Conference (ISSCC)*, Feb. 2023, pp. 496–498, doi: 10.1109/ISSCC42615.2023.10067563.

[8] C. X. Xue *et al.*, "A 1Mb Multibit ReRAM Computing-In-Memory Macro with 14.6ns Parallel

MAC Computing Time for CNN Based AI Edge Processors," *Dig. Tech. Pap. - IEEE Int. Solid-State Circuits Conf.*, vol. 2019-February, pp. 388–390, Mar. 2019, doi: 10.1109/ISSCC.2019.8662395.

[9] D. Joksas *et al.*, "Nonideality-Aware Training for Accurate and Robust Low-Power Memristive Neural Networks," *Adv. Sci.*, vol. 9, no. 17, p. 2105784, Jun. 2022, doi: 10.1002/ADVS.202105784.

[10] I. Chakraborty, D. Roy, and K. Roy, "Technology Aware Training in Memristive Neuromorphic Systems for Nonideal Synaptic Crossbars," *IEEE Trans. Emerg. Top. Comput. Intell.*, vol. 2, no. 5, pp. 335–344, Oct. 2018, doi: 10.1109/TETCI.2018.2829919.

[11] L. Chen *et al.*, "Accelerator-friendly neural-network training: Learning variations and defects in RRAM crossbar," in *Proceedings of the 2017 Design, Automation and Test in Europe, DATE 2017*, May 2017, pp. 19–24, doi: 10.23919/DATE.2017.7926952.

[12] "Design Guidelines of RRAM based Neural-Processing-Unit: A Joint Device-Circuit-Algorithm Analysis | IEEE Conference Publication | IEEE Xplore." https://ieeexplore.ieee.org/document/8807058 (accessed Apr. 03, 2023).

[13] X. Peng, R. Liu, and S. Yu, "Optimizing Weight Mapping and Data Flow for Convolutional Neural Networks on RRAM Based Processing-In-Memory Architecture," in *2019 IEEE International Symposium on Circuits and Systems (ISCAS)*, May 2019, pp. 1–5, doi: 10.1109/ISCAS.2019.8702715.

[14] M. V. Beigi, "Thermal-Aware Optimizations for Emerging Technologies in 3D-Stacked Chips," Northwestern University, 2019.

[15] P. Sun *et al.*, "Thermal crosstalk in 3-dimensional RRAM crossbar array," *Sci. Rep.*, vol. 5, no. 1, p. 13504, Aug. 2015, doi: 10.1038/srep13504.

[16] C. Walczyk *et al.*, "Impact of temperature on the resistive switching behavior of embedded HfO2-based RRAM devices," *IEEE Trans. Electron Devices*, vol. 58, no. 9, pp. 3124–3131, Sep. 2011, doi: 10.1109/TED.2011.2160265.

[17] K. Smagulova, M. E. Fouda, and A. Eltawil, "Thermal Heating in ReRAM Crossbar Arrays: Challenges and Solutions," Dec. 2022, doi: 10.48550/arXiv.2212.13707.

[18] K. Smagulova, M. E. Fouda, F. Kurdahi, K. N. Salama, and A. Eltawil, "Resistive Neural Hardware Accelerators," *Proc. IEEE*, vol. 111, no. 5, pp. 500–527, Sep. 2021, doi: 10.1109/JPROC.2023.3268092.

[19] X. Liu, M. Zhou, T. S. Rosing, and J. Zhao, "HR 3 AM: A Heat Resilient Design for RRAM-based Neuromorphic Computing," in *2019 IEEE/ACM International Symposium on Low Power Electronics and Design (ISLPED)*, Jul. 2019, pp. 1–6, doi: 10.1109/ISLPED.2019.8824926.

[20] M. Zhou, M. Imani, S. Gupta, and T. Rosing, "Thermal-aware design and management for search-based in-memory acceleration," Jun. 2019, doi: 10.1145/3316781.3317923.

[21] P.-Y. Chen, F.-Y. Gu, Y.-H. Huang, and I.-C. Lin, "WRAP: Weight RemApping and Processing in RRAM-based Neural Network Accelerators Considering Thermal Effect," in *2022 Design, Automation & Test in Europe Conference & Exhibition (DATE)*, Mar. 2022, pp. 1245–1250, doi: 10.23919/DATE54114.2022.9774678.

[22] S. Han, J. Pool, J. Tran, and W. J. Dally, "Learning both Weights and Connections for Efficient Neural Networks," Jun. 2015, [Online]. Available: http://arxiv.org/abs/1506.02626.

[23] M. V. Beigi and G. Memik, "Thermal-aware Optimizations of ReRAM-based Neuromorphic Computing Systems," in *2018 55th ACM/ESDA/IEEE Design Automation Conference (DAC)*, Jun. 2018, pp. 1–6, doi: 10.1109/DAC.2018.8465880.

[24] J. Meng *et al.*, "Temperature-Resilient RRAM-Based In-Memory Computing for DNN

Inference," *IEEE Micro*, vol. 42, no. 1, pp. 89–98, Jan. 2022, doi: 10.1109/MM.2021.3131114.
